# Computational redesign and directed evolution of a lanthanide-dependent photoredox enzyme for enantioselective diol cleavage

**DOI:** 10.1039/d5sc08010j

**Published:** 2026-03-17

**Authors:** Florian Leiss-Maier, Joshua Behringer, Ghulam Mustafa, Anna Heider, Rahel Mühlhofer, Andreas S. Klein, Michael Groll, Cathleen Zeymer

**Affiliations:** a Center for Functional Protein Assemblies & Department of Bioscience, TUM School of Natural Sciences, Technical University of Munich (TUM) 85748 Garching Germany cathleen.zeymer@tum.de

## Abstract

*De novo* designed metalloenzymes and photoenzymes are a valuable addition to the biocatalytic toolbox. We previously introduced PhotoLanZymes (PLZ), a family of lanthanide-dependent photoredox enzymes that enable radical carbon–carbon bond cleavages of diol substrates upon Ce(iii/iv) binding and visible-light irradiation. While rational optimization increased their catalytic activity and photostability, the first generation of PLZ variants was limited by slow lanthanide binding and a lack of enantioselectivity. Here, we demonstrate that coupling computational redesign with directed evolution provides an effective strategy to overcome these limitations. First, we reduced the cavity size to enhance substrate interactions with the protein's active site, which facilitated initial enantiocontrol. Simultaneously, the AI-guided redesign approach improved the lanthanide binding kinetics. We then performed directed evolution to selectively accelerate the photocatalytic turnover for one of the substrate enantiomers, yielding a PLZ variant with markedly improved enantioselectivity. These results underscore the value of integrating AI-guided protein design with laboratory evolution to obtain stereoselective *de novo* metalloenzymes and photoenzymes.

## Abstract 2

A combined approach of AI-guided protein redesign and directed evolution yields enantioselective PhotoLanZymes for cerium-dependent photocatalytic C–C bond cleavage.

## Graphical abstract

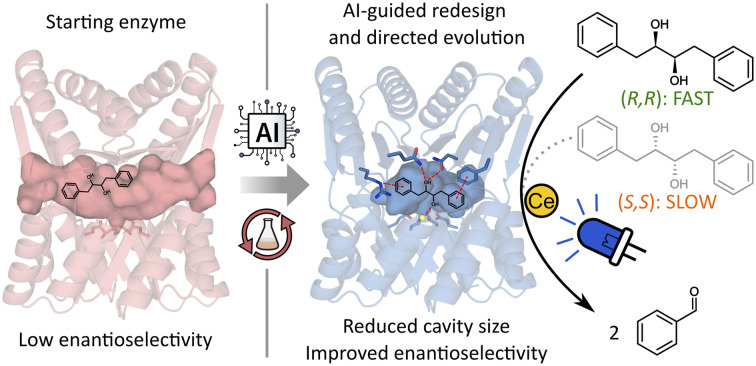

## Introduction

The computational design of proteins has seen remarkable improvements in recent years, in particular after the implementation of deep learning-based methods.^1–3^ This increase in design accuracy has yielded high affinity binders^4–6^ and tailor-made protein scaffolds.^7,8^ However, the computational design of catalytically active enzymes remains challenging, as it necessitates the precise arrangement of catalytic residues in three-dimensional space with sufficient conformational flexibility.^9–11^

In an alternative approach to generate artificial enzymes with new-to-nature activity, protein scaffolds can be equipped with cofactors that catalyze challenging reactions inside of the chiral protein environment, thereby enabling enantioselective transformations. Options to endow protein scaffolds with catalytic function comprise photosensitizers, to produce artificial photoenzymes,^12–15^ and metal cofactors, to generate artificial metalloenzymes. The latter make up a big portion of the *de novo* enzymes known to date and include a range of potent hybrid catalysts for a variety of applications.^16^ Frequently used metal cofactors encompass transition metals customary to both nature and synthetic chemistry, such as copper^17,18^ and iron,^19–21^ but also abiological metals like titanium^22^ have been used in the past. Here, we focus on lanthanide ions, a currently underexplored class of enzyme cofactors known to catalyze a broad range of reactions, including photoredox transformations.^23–26^

Our group has recently reported a *de novo* designed photoenzyme that leverages lanthanide ions as photoredox cofactors to catalyze radical reactions upon visible-light irradiation.^27^ The enzyme called PhotoLanZyme (PLZ) uses a datively coordinated Ce(iii/iv) ion to catalyze the photoinduced C–C bond cleavage of diol substrates to yield carbonyl products. The protein scaffold consists of a *de novo* TIM barrel domain coordinating the lanthanide ion with four glutamate residues and a dimeric ferredoxin domain that acts as a lid above the active site.^28^ Induced by photoexcitation, the central Ce(iv) is proposed to engage in ligand-to-metal charge transfer (LMCT) with its diol substrates to catalyze radical diol cleavages, as described previously.^29–31^ Rational engineering of the initial design PLZ1.0 generated PLZ1.4, a variant with optimized photostability and reduced off-target metal binding. This resulted in improved yields for a variety of diol substrates.^27^ However, a major limitation was the enzyme's lack of enantioselectivity, which we set out to tackle in this work. Our goal was thus to redesign and evolve PLZ to enable the kinetic resolution of racemic mixtures of diol substrates.

Here we describe a computational redesign strategy that yielded a PLZ variant with an adjusted cavity size, resulting in a 100-fold higher *k*_cat_/*K*_M_ for the model substrate hydrobenzoin and initial enantioselectivity for a slightly larger diol substrate. Directed evolution of this improved photoenzyme could further increase the enantioselectivity. A crystal structure of the final variant validated the success of our redesign strategy. These results highlight that the combination of computational and experimental optimization is a promising strategy to improve activity and selectivity of *de novo* enzymes.

## Results and discussion

### Computational redesign to adjust the size of the substrate binding cavity

**Fig. 1:**
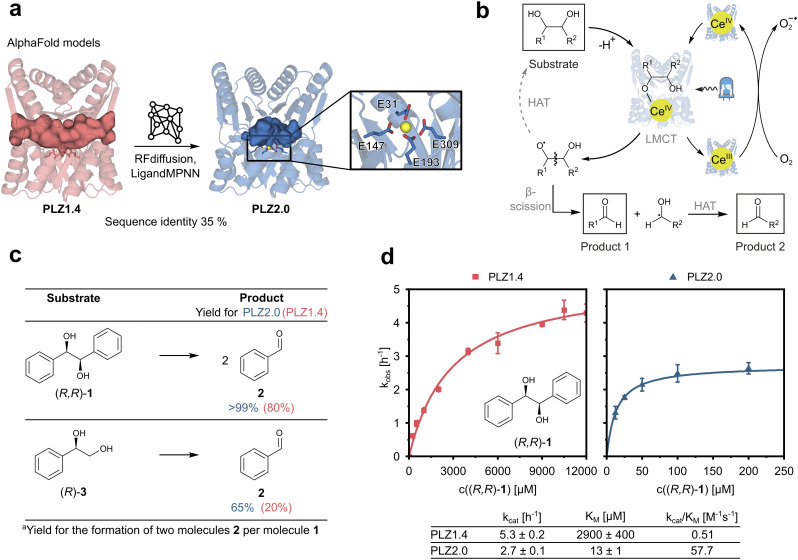
PLZ redesign and catalytic efficiency. (a) Structural models of PLZ1.4 (red, left) and PLZ2.0 (blue, right), showing the reduction in cavity size after computational redesign (depicted as internal protein surface). Right: the cerium ion is shown in yellow, coordinating glutamate residues are shown as sticks. (b) Proposed mechanism of the photoinduced cerium-dependent radical diol cleavage as reported previously in organic solvent.^30^ LMCT: ligand-to-metal charge transfer. HAT: hydrogen atom transfer. (c) Comparison of yields obtained for model substrates 1 and 3 using PLZ2.0 and PLZ1.4 with 5 mol% catalyst loading after 24 h irradiation at 410–420 nm. (d) Michaelis–Menten kinetics for substrate (*R*,*R*)-1 using the photoenzymes PLZ1.4 and PLZ2.0, in red and blue, respectively.

To improve activity and selectivity of PLZ1.4, the previously reported and rationally engineered cerium-dependent photoenzyme,^27^ we first analyzed its structural features. We hypothesized that the active site cavity was too large to efficiently pre-orient diol substrates near the catalytically active cerium ion and thus to discriminate between stereoisomers. Therefore, we set out to computationally redesign and optimize PLZ1.4 in order to reduce the size of the binding pocket. We first deleted the four flexible linker regions originally connecting the ferredoxin domain and the TIM barrel and then manually lowered the lid domain by 3 Å to generate a smaller cavity. This adjusted scaffold structure was used as an input for protein redesign using AI-guided tools. The connecting linkers were re-introduced by RFdiffusion and the newly generated domain–domain interface was optimized using partial diffusion.^32^ Following this step, we generated sequences for the obtained fold using LigandMPNN,^33^ taking into account the desired position of the diol substrate. Here, the four coordinating glutamate residues remained fixed in their position to assure high affinity for lanthanide cofactors (Fig. 1a and S1). After structure prediction, first using ESMfold^34^ and then Alphafold2 (ref. 35) in Colabfold,^36^ the designs with the highest confidence levels (predicted local distance difference test, pLDDT) were selected. These structures were manually evaluated by comparing cavity size, shape, and domain-specific prediction confidence. Off-target metal binding sites were identified using BioMetAll^37^ and removed by mutagenesis (E to Q, D to N, and C to S mutations). Finally, two loops with low pLDDT scores in the ferredoxin domains were shortened to improve domain rigidity and model confidence. The resulting protein, called PLZ2.0, has 35% sequence identity to PLZ1.4 and is shortened by 14 residues in total (12 residues less due to linker remodeling and 2 residues less in the ferredoxin domains). It retains the overall fold, but with a significantly reduced cavity volume (Fig. 1a and S1).

### PLZ2.0 shows improved catalytic efficiency

Even though the scaffold redesign process only intended to decrease the cavity size, AI-guided sequence redesign produced additional benefits: first, PLZ2.0 showed significantly faster lanthanide binding kinetics than PLZ1.4. When following the specific tryptophan-enhanced Tb(iii) luminescence as a binding readout, PLZ1.4 needed more than two hours at room temperature to reach the maximum signal,^27^ while for PLZ2.0 complete metal binding was observed within 5 minutes (Fig. S2). This may be due to an improved conformational equilibrium. In a previous study, we found that MPNN-based sequence redesign stabilized the binding-competent conformation of the original PLZ1.0 scaffold by introducing additional molecular interactions in the dimer interface.^38^ We hypothesize that a similar effect is also responsible for the accelerated lanthanide binding here, when redesigning for a smaller cavity. Importantly, nanomolar lanthanide affinity was retained in the redesign process, as shown by titration experiments (Fig. S3–S6). A knockout variant (PLZ2.0_KO), in which the coordinating glutamate residues (E31, E147, E193, E309) were replaced by glutamines, did not show any lanthanide binding in the active site (Fig. S2).

Next, we tested the catalytic activity of PLZ2.0 in cerium-dependent photoinduced diol cleavage reactions. The proposed mechanism of the equivalent reaction in organic solvent proceeds *via* the coordination of the diol to Ce(iv), followed by visible light-induced LMCT (Fig. 1b).^30^ The resulting alkoxy radical can subsequently undergo β-scission, producing a carbonyl product and a carbon-centered radical. This radical can further undergo hydrogen atom transfer (HAT) to produce the second carbonyl product. Finally, Ce(iii) is proposed to be re-oxidized to Ce(iv) by atmospheric oxygen, as recently observed in organic solvents.^39^ While mechanistic details of the photoenzymatic transformation remain unknown, UV/Vis absorption spectra support that the Ce(iv)-bound protein can be excited at around 400 nm (Fig. S7).

Indeed, reducing the size of the active site cavity showed a beneficial outcome in our test reactions. Using our previously reported standard reaction conditions with 5 mol% catalyst loading and irradiation for 24 h at 410–420 nm, we observed quantitative diol cleavage for the model substrate hydrobenzoin (*R*,*R*)-1 to benzaldehyde 2, compared to 80% yield for PLZ1.4. Even the smaller and less reactive diol (*R*)-3 yielded 65% of product 2, which marked a significant increase compared to the 20% yield observed with PLZ1.4 (Fig. 1c). The knock-out variant PLZ2.0_KO showed very low background activity, attributed to only trace amounts of lanthanides binding to the protein surface (Fig. S8). This may also be a beneficial side effect of the redesign process, in which unspecific metal binding was further reduced.

To assess whether the reduced cavity size restricted access for larger substrates, we performed docking with a range of diols of increasing size using Glide.^40^ Applying a blind docking mode, where no binding cavity was pre-defined, we observed that the experimentally confirmed substrates 1 and 4 as well as di-methylated variants can enter the active site (Fig. S9). Larger diols were repeatedly excluded, indicating a stringent size selectivity.

We then measured Michaelis–Menten kinetics of PLZ1.4 and PLZ2.0 using substrate (*R*,*R*)-1. Strikingly, the *K*_M_ value was lowered more than 200 fold, from 2900 ± 400 µM for PLZ1.4 to 13 ± 1 µM for PLZ2.0, while *k*_cat_ slightly decreased by a factor of two (Fig. 1d). The resulting 100-fold improvement in catalytic efficiency can thus be attributed exclusively to an optimized substrate binding behavior, which is consistent with the hypothesis that the smaller cavity would allow a better preorganization of the diol for the reaction. Additionally, a change in conformational flexibility of the inter-domain linkers may improve substrate positioning in the active site.^41–43^ Replacing the four highly flexible tri-glycine linkers connecting the TIM barrel to the ferredoxin lid by shorter and more rigid sequences (DTD, QG, DQD, and LG, respectively) could have further enhanced substrate binding by reducing twisting motions previously observed for similar scaffolds.^38^ Encouraged by these results, we decided to investigate whether the newly designed active site could also discriminate between individual diol substrate enantiomers.

### The smaller cavity facilitates initial enantiocontrol

**Fig. 2:**
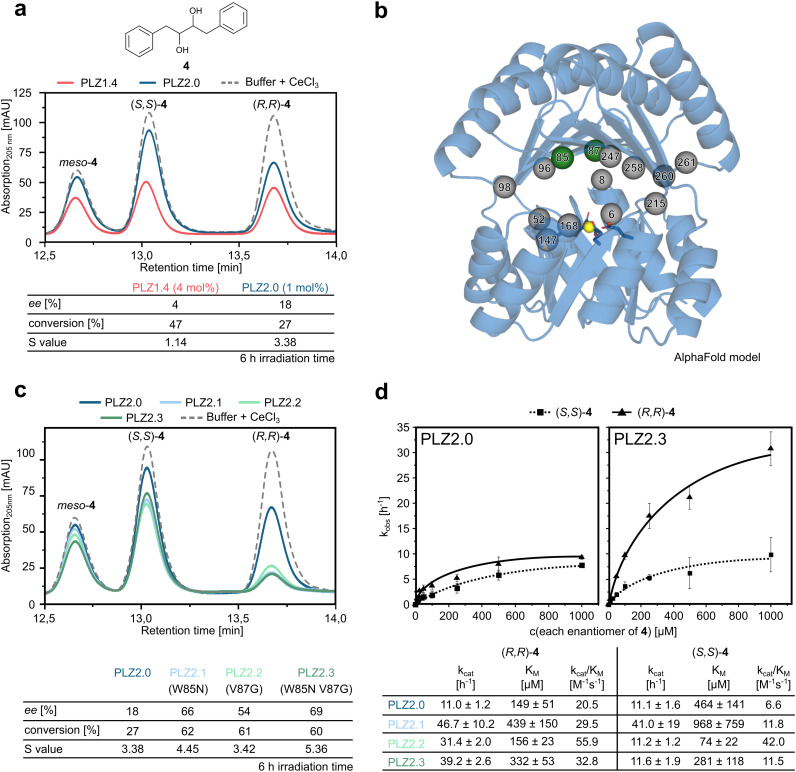
Directed evolution to improve the enantioselectivity towards substrate 4. (a) Chiral HPLC of the unreacted substrate after 6 hours of irradiation with PLZ1.4 (4 mol% catalyst loading) and PLZ2.0 (1 mol% catalyst loading). The selectivity value *S* was calculated from measured conversion and ee. (b) Positions that were targeted for directed evolution. W85 and V87 are depicted as green spheres, other positions are depicted as grey spheres. The cerium ion is shown as a yellow sphere, coordinating residues are shown as sticks. (c) Chiral HPLC of the unreacted substrate after 6 h irradiation with PLZ2.0, PLZ2.1, PLZ2.2, and PLZ2.3 (1 mol% catalyst loading). (d) Michaelis–Menten kinetics analyzed for the individual enantiomers of substrate 4 comparing the starting point PLZ2.0 (left) and the evolved variant PLZ2.3 (right). Kinetic parameters for the single-mutation variants PLZ2.1 and PLZ2.2 are listed in the table below. The corresponding curves are shown in Fig. S14.

We next compared the enantioselectivity of the redesigned photoenzyme PLZ2.0 with that of the parent variant PLZ1.4, using both hydrobenzoin (1) and the slightly bulkier diol substrate 4. Because of the higher catalytic activity of PLZ2.0, catalyst loading was reduced to 1 mol% in these experiments. While no pronounced selectivity was observed for 1 (Fig. S10), promising initial stereocontrol was achieved for 4, where the redesigned PLZ2.0 could discriminate between the (*R*,*R*) and (*S*,*S*) enantiomer (Fig. 2a). When analyzing the unreacted substrate 4 by chiral HPLC after 6 h irradiation time, we found an enantiomeric excess (ee) of 18% at 27% conversion, which translates into a selectivity factor (*S*) of 3.4—substantially higher than the value of 1.1 observed for PLZ1.4. This advancement in selectivity demonstrated that PLZ2.0 could potentially be optimized for the kinetic resolution of racemic diol substrates. These results established PLZ2.0 as an improved catalyst and thus a promising starting point for directed evolution.

### Directed evolution improves the enantioselectivity

We screened site-saturation libraries of residues lining the active site of PLZ2.0 (Fig. 2b). In a first round we randomized positions L52, W85, V87, A98, Q168, and A247 using a reduced amino acid alphabet encoded by NDT codons (including amino acids D, R, Y, F, I, S, C, H, N, G, L, V). After expression in 96-well plate format, cells were lysed and the highly thermostable *de novo* protein was purified by a heat step (1 h at 80 °C), while remaining DNA was removed by precipitation with polythylenimine (Fig. S11). The clean, cerium(iii)-loaded enzymes were irradiated in an LED photoreactor equipped with a custom cooling system for microtiter plates. All reactions were then analyzed by chiral HPLC to detect variants with improved enantioselectivity. We were able to identify two hits: W85N (=PLZ2.1) and V87G (=PLZ2.2). After large-scale purification of these enzymes we confirmed that the mutations increased both enantioselectivity and activity of the photoenzyme (Fig. 2c). Combining the two point mutations yielded PLZ2.3 (=PLZ2.0 W85N V87G). The double mutant showed further enhanced selectivity with an *S* value of 5.4. This improvement demonstrates that the PLZ family can be optimized both in selectivity and catalytic efficiency and therefore is a promising class of catalysts for kinetic resolution experiments. Screening of ten further single site-saturation libraries (see Fig. 2b) based on PLZ2.3 unfortunately did not yield any additional hits. We therefore decided to determine the kinetic parameters of all available enzyme variants towards the enantiomers of 4 to improve our understanding of the underlying mechanism of selectivity.

For the Michaelis–Menten experiments, we also removed *meso*-4 from the substrate mixture (Fig. S13) to be able to better assess the individual kinetics for the (*R*,*R*) and (*S*,*S*) enantiomer. Compared to PLZ2.0, *k*_cat_ and *K*_M_ for (*S*,*S*)-4 remained in similar regimes for PLZ2.3, while for (*R*,*R*)-4 the *k*_cat_ increased almost 4-fold (Fig. 2d). Despite being accompanied by a slight increase in *K*_M_ for the (*R*,*R*)-enantiomer, this resulted in the improved selectivity observed for the double mutant.

We can therefore conclude that the two mutations selectively increased the reaction rate for only (*R*,*R*)-4, while hardly affecting the behavior of the enzyme towards (*S*,*S*)-4. When comparing the kinetic parameters to the single-mutant variants PLZ2.1 and PLZ2.2 it becomes apparent that PLZ2.1 exhibits a higher *k*_cat_ than PLZ2.3, while PLZ2.2 shows an improved *K*_M_ (Fig. 2d and S14). Although these effects are partially attenuated in the double mutant, PLZ2.3 exhibits the highest enantioselectivity, as judged by the difference in *k*_cat_/*K*_M_ for (*R*,*R*)-4*versus* (*S*,*S*)-4, in line with our evolutionary focus on improved enantioselectivity. Nevertheless, the improvement between the single mutants and PLZ2.3 is modest and less than additive, indicating an interdependence between the individual point mutants. To quantify this effect, we performed a double-mutant cycle analysis using *k*_cat_/*K*_M_ for (*R*,*R*)-4. The resulting interaction free energy (ΔΔ*G*^‡^_int_ ≈ +0.5 kcal mol^−1^) suggests antagonistic epistasis, consistent with the double mutant performing worse than predicted from the individual substitutions. Interestingly, when assessing the enantioselectivity of PLZ2.3 towards the original substrate 1, we observed a slight selectivity for the opposite (*S*,*S*)-enantiomer, although less pronounced (Fig. S15).

Next, we determined if the lanthanide binding affinity was impacted by the mutations in the active site of PLZ2.3. We transitioned to isothermal titration calorimetry (ITC), as the tryptophan antenna (W85) required for the sensitized terbium luminescence readout was no longer present. At pH 8.5, lanthanide hydrolysis^44^ impaired the measurements and a shift to lower pH was required. Slightly lower affinities due to partial protonation of the coordinating residues^45^ were thus expected. We determined cerium and terbium binding affinities between 50 nM and 350 nM (Fig. S4–S6) and thus concluded that the affinity for lanthanides did not drastically suffer from the introduced point mutations.

To assess potential photodamage during extended irradiation, protein samples containing CeCl_3_ with or without substrate were analyzed by SDS-PAGE and LC-MS after 6 hours of irradiation. SDS-PAGE revealed no evidence of protein crosslinking or degradation (Fig. S16). LC-MS showed a pattern of several +16 Da mass shifts for PLZ2.0 and PLZ2.3 (Fig. S17), consistent with oxidative modifications of residues such as Trp or Met, as also observed for PLZ1.4.^27^ In the presence of substrate 4, additional adduct peaks were observed. These effects were more pronounced for PLZ2.3 than for PLZ2.0, consistent with its higher turnover. Importantly, the unmodified protein remained the dominant species, and simple oxidation events are not expected to inactivate the enzyme significantly.

Finally, we performed a buffer screen to investigate the potential influence of buffer components on the photoreaction. Simple inorganic buffers such as phosphate and carbonate were unsuitable, as they form insoluble lanthanide salts. We thus tested a set of common Good's buffers and found that HEPES and MOPS performed equally well, while all others significantly decreased the activity (Fig. S12). Due to their various functional groups, including alcohol, amine, carboxylic acid, sulfonic acid, Good's buffers may interfere with redox chemistry,^46^ potentially serving as sacrificial reductants, HAT agents, or radical scavangers.^47^ Particularly relevant for our reaction is the reported formation of HEPES radicals in the presence of superoxide,^48^ which is generated in the proposed regeneration of Ce(iv) by molecular oxygen. The buffer may thus mediate the level of oxidative damage to the protein, but could also actively participate in the radical reaction mechanism. Furthermore, buffers containing diols or triols, such as Bicine, Tris, Tricine, may compete with the substrate, which is in agreement with the observed activity loss. Overall, it can be concluded that a fully innocent buffer system is not available, positive and negative effects are conceivable, and the choice of buffer was thus guided by optimal enzyme stability, activity, and stereoselectivity.

### Structural analysis by X-ray crystallography and MD simulations

**Fig. 3:**
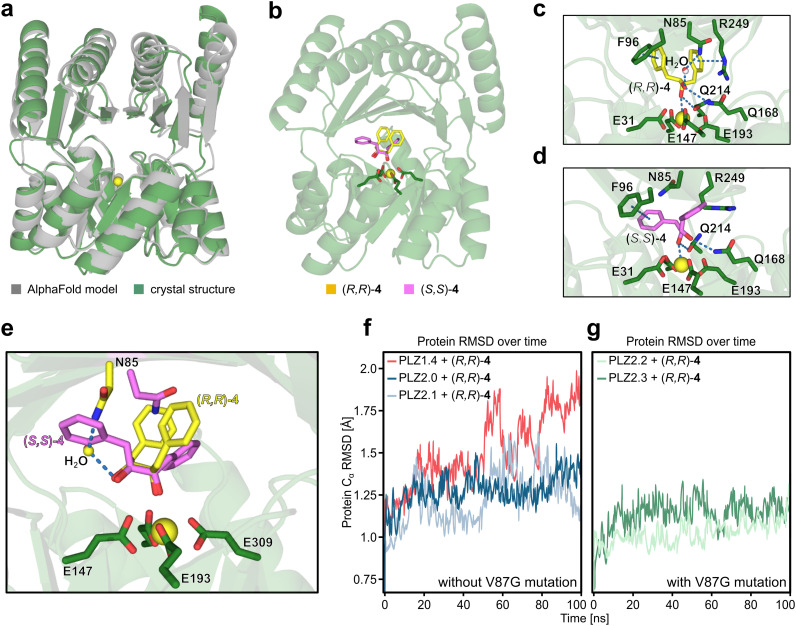
Structure and dynamics of PLZ variants. (a) Overlay of the crystal structure of PLZ2.3 (green) and the AlphaFold3 model of the final design of PLZ2.0 (grey). The bound cerium ion is shown as a yellow sphere. (b) Docking of the substrate enantiomers into the crystal structure. (*R*,*R*)-4 is shown as yellow sticks, (*S*,*S*)-4 is shown as violet sticks. (c) Binding pose of (*R*,*R*)-4 in PLZ2.3 extracted from the MD simulation. Notable interactions with nearby residues are indicated as blue dashed lines. (d) Binding pose of (*S*,*S*)-4 in PLZ2.3 extracted from the MD simulation. Notable interactions with nearby residues are indicated as blue dashed lines. (e) Potential explanation for the enantioselectivity in PLZ2.3: the hydrogen bonding network between the substrate, a transiently observed water molecule, and N85 can only be formed with (*R*,*R*)-4. For (*S*,*S*)-4, this interaction is sterically hindered. (f) C_α_ RMSD of PLZ1.4, PLZ2.0 and PLZ2.1 with bound (*R*,*R*)-4 over the course of the MD simulations shows higher variability in RMSD for variants without the V87G mutation. (g) C_α_ RMSD of PLZ2.2 and PLZ2.3 with bound (*R*,*R*)-4 over the course of the MD simulations shows reduced variability of RMSD for variants carrying the V87G mutation.

To further improve our understanding of the enzyme scaffold, we determined the crystal structure of PLZ2.3 bound to cerium(iii) at a resolution of 2.1 Å (Fig. 3a). The experimental structure is in very good agreement with the AlphaFold3 model of our design output for PLZ2.0. The backbone RMSD of 1.59 Å originates from a slightly different orientation of both domains to each other. Also a template modeling (TM) score of 0.94 indicates a successful design.^49^ This is further supported by highly similar circular dichroism spectra of all PLZ variants (Fig. S18). As evident from the crystal structure, the size of the active site cavity is reduced compared to PLZ1.4 and the coordinated cerium(iii) ion is located at the desired position.

We next modeled PLZ1.4 using Boltz-2 (ref. 50) and generated models of PLZ2.0, PLZ2.1, and PLZ2.2 from the crystal structure of PLZ2.3. We docked the individual enantiomers of substrate 4 using Glide.^40^ We then performed molecular dynamics (MD) simulations to examine differences between the variants and rationalize the observed selectivity and epistasis (Fig. 3b–g). The cavity volume of the redesign was reduced as intended. Averaging the calculated pocket size over a 100 ns MD trajectory of the proteins with docked (*R*,*R*)-4 gave a volume of *ca.* 730 Å^3^ for PLZ2.3, compared to *ca.* 1640 Å^3^ for PLZ1.4, as calculated by MDpocket (Fig. S19).^51^ As a consequence, this enabled a more stable binding mode and distinct molecular interactions between protein and substrate. Comparison of (*R*,*R*)-4 and (*S*,*S*)-4 binding modes in PLZ2.3 indicates that both enantiomers position their hydroxyl groups similarly but differ in their aromatic ring orientation (Fig. 3b and e). While both retain hydrogen bonding to Q168 and Q214, as well as π–π stacking with F96, only the (*R*,*R*) enantiomer forms a N85-mediated hydrogen-bond network and a cation–π interaction with R249 during the simulation (Fig. 3c and d). These interactions are sterically inaccessible for the (*S*,*S*) enantiomer (Fig. 3e). The MD results thus indicate a full fixation of both hydroxyl groups and aromatic rings only for (*R*,*R*)-4. We therefore assume that N85 and R249 are crucial for the enantioselectivity of PLZ2.3. Additionally, distinct second-sphere interactions could influence the LMCT step, giving rise to slightly different activation barriers for the two enantiomers.

Furthermore, our MD simulations suggest that the stabilization of (*R*,*R*)-4 in the individual PLZ variants arises from distinct and partially mutually exclusive interaction networks, providing a structural rationale for the antagonistic epistasis observed in the double-mutant cycle analysis. PLZ2.0 shows a hydrogen bond between the substrate and Q214. Additionally, both phenyl rings of 4 are stabilized by cation–π interactions with R249 or π–π-interactions with W85 (Fig. S20). In PLZ2.1, (*R*,*R*)-4 is positioned through a transient water-mediated hydrogen-bond network involving N85, accompanied by a cation–π interaction between R249 and the substrate (Fig. S21). In PLZ2.2, the V87G mutation removes a steric restriction of L8, allowing it to shift inward and enabling a concerted loop rearrangement that positions Q6 for hydrogen bonding to the substrate (Fig. S24). This reorganization coincides with a pronounced stabilization of global RMSD over the MD trajectory, indicating increased scaffold stability, which is observed for both variants carrying the V87G mutation (Fig. 3f and g). While aromatic interactions with W85 are observed, PLZ2.2 lacks the R249 cation–π interaction (Fig. S22). In the double mutant PLZ2.3, W85 is mutated, and instead F96 engages in T-shaped π–π stacking with the substrate.^52,53^ In addition, (*R*,*R*)-4 forms hydrogen bonds to Q169 and Q214, a transient water-mediated interaction with N85, and a cation–π interaction with R249 (Fig. 3c and S23).

We attribute the non-additivity of the individual point mutations to the fact that the decisive hydrogen bond interactions for PLZ2.1 and PLZ2.2 are mutually exclusive: Q6 and the N85/Q169 network reside on opposite sides of the active site and cannot be engaged simultaneously (Fig. S25). Consequently, PLZ2.3 adopts the N85/Q169-based interaction pattern, explaining the lack of additive improvement in catalytic efficiency and the antagonistic epistasis observed in the double-mutant cycle analysis.

Overall, our results underline the successful protein redesign using AI-guided tools, in particular when comparing to PLZ1.4, which was based on a scaffold that originated from physics-based *de novo* design.^28^

### Kinetic resolution of *rac*-4 using PLZ2.3

**Fig. 4:**
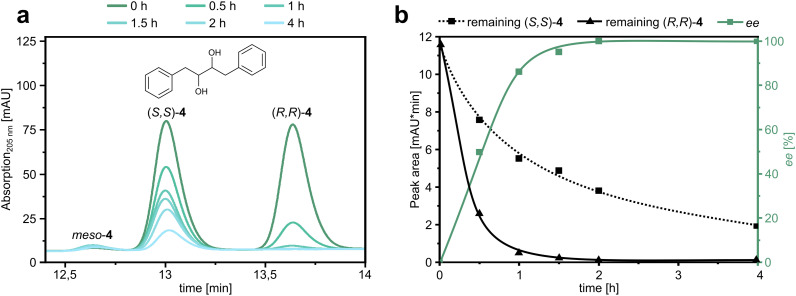
Kinetic resolution of diol substrate 4 using PLZ2.3. (a) HPLC chromatograms of the unreacted substrate enantiomers at different time points of irradiation in the presence of PLZ2.3. (b) Kinetic analysis of the peak areas shown in Fig. 4a and resulting ee over the course of the experiment.

As a final experiment, we asked whether PLZ2.3 was selective enough to catalyze the kinetic resolution of *rac*-4. We irradiated a mixture of (*R*,*R*)-4 and (*S*,*S*)-4 in the presence of PLZ2.3 at a catalyst loading of 5 mol% and took samples at several time points to follow the consumption of the individual enantiomers (Fig. 4a). As expected, (*R*,*R*)-4 was consumed significantly faster than the (*S*,*S*)-enantiomer. After two hours, the ee of the remaining substrate approached 100%, as the (*R*,*R*)-enantiomer was completely consumed (Fig. 4b). The kinetic resolution experiment therefore yields enantiopure (*S*,*S*)-4 if the reaction is stopped after 2 h. However, due to the remaining enzymatic activity towards this disfavored enantiomer, the yields are much lower than practically desired.^54^ To increase the applicability in a scaled-up reaction, further improvement of the *S* value would be required. Nevertheless, this proof-of-concept experiment showed that the PLZ family of photoenzymes can be used in a stereoselective fashion.

## Conclusions

Photoenzymes are an emerging class of biocatalysts.^55^ Here we show that PhotoLanZymes – a family of lanthanide-dependent, *de novo* designed photoredox enzymes^27^ – can achieve enantioselective catalysis, which is a decisive feature for their future application. We combined AI-guided protein redesign and directed evolution to enhance both catalytic efficiency and enantiocontrol for a cerium-dependent radical C–C bond cleavage reaction that proceeds upon visible-light irradiation. The accessibility of powerful deep learning tools for protein design^1–3^ makes this a general approach to gain conformational stability and favorable dynamics in a *de novo* enzyme of interest, thereby generating viable starting points for directed evolution. Ongoing efforts concerning the PLZ family of photoredox enzymes focus on broadening their reaction scope to other lanthanide-dependent transformations, including radical C–C bond forming reactions.

## Author contributions

C. Z., A. S. K. and F. L. M. conceived the project. F. L. M. produced, evolved, and characterized the photoenzymes. J. B. performed the computational redesign and experiments for the revision. R. M. supported the filtering of initial design outputs. G. M. performed and evaluated the MD simulations. A. S. K. performed initial experiments with PLZ1.4. A. H. and M. G. performed protein crystallization and structure determination. F. L. M. and C. Z. wrote the manuscript. All authors have given approval to the final version of the manuscript.

## Conflicts of interest

There are no conflicts to declare.

## Supplementary Material

SC-OLF-D5SC08010J-s001

## Data Availability

The crystal structure of PLZ2.3 has been deposited in the RCSB Protein Data Bank (PDB entry: 9SZU). All raw data files associated with this study will be openly available in the university's research data repository “mediaTUM” at https://doi.org/10.14459/2026mp1840493. Supplementary information (SI): SI figures, detailed procedures and results for all reported experiments, and data for compound characterization. See DOI: https://doi.org/10.1039/d5sc08010j.
