# Supplementary material for: Computational redesign and directed evolution of a lanthanide-dependent photoredox enzyme for enantioselective diol cleavage
Source: Chem Sci. 2026 Mar 17;17(19):9552–61. doi: 10.1039/d5sc08010j (PMC13010360; doi:10.1039/d5sc08010j)
Supplement: SC-017-D5SC08010J-s001 [file SC-017-D5SC08010J-s001.pdf]

Supplementary Information (SI) for Chemical Science.
This journal is © The Royal Society of Chemistry 2026

Supplementary Information

Computational redesign and directed evolution of a lanthanide-dependent photoredox enzyme for enantioselective diol cleavage

Florian Leiss-Maier, Joshua Behringer, Ghulam Mustafa, Anna Heider, Rahel Mühlhofer, Andreas S. Klein, Michael Groll, and Cathleen Zeymer*

Center for Functional Protein Assemblies & Department of Bioscience, TUM School of Natural Sciences, Technical University of Munich (TUM), 85748 Garching, Germany

* E-mail: cathleen.zeymer@tum.de

Content

1. Supporting figures ... 4
2. General methods ... 29
3. Biochemical methods ... 32
   - 3.1. Molecular cloning ... 32
     - 3.1.1. Generation of site-saturation libraries for directed evolution ... 33
     - 3.1.2. Primers used in this study ... 33
   - 3.2. Expression, purification, and analysis of proteins ... 37
     - 3.2.1. Culture conditions ... 37
     - 3.2.2. Buffers used for protein purification ... 37
     - 3.2.3. Protein expression and purification ... 37
     - 3.2.4. Expression and purification of proteins in a 96-well format ... 38
     - 3.2.5. Circular dichroism (CD) spectroscopy ... 38
     - 3.2.6. Protein mass spectrometry ... 39
     - 3.2.7. Photostability studies ... 39
     - 3.2.8. Tb(III) binding kinetics ... 39
     - 3.2.9. Luminescence-based titrations to determine Tb(III) binding affinities ... 39
     - 3.2.10. Isothermal titration calorimetry (ITC) to determine lanthanide binding affinities ... 41
     - 3.2.11. UV/Vis absorption studies ... 42
     - 3.2.12. DNA and protein sequences of all constructs ... 43
4. Computational methods ... 47
   - 4.1. Design of PLZ2.0 using RFdiffusion and LigandMPNN ... 47
   - 4.2. Docking and molecular dynamics (MD) simulations ... 48
   - 4.3. Blind docking of larger substrates ... 49
   - 4.4. 2D interaction representation ... 49
5. Enzymatic assays ... 50
   - 5.1. Procedures for photoenzymatic reactions ... 50
     - 5.1.1. 6-vial photoreactor setup ... 50
     - 5.1.2. Vial-based photoreaction procedure ... 50
     - 5.1.3. Procedure for plate-based photoreactions for directed evolution ... 50
     - 5.1.4. Determination of yields by quantification of HPLC peaks ... 51
     - 5.1.5. Determination of enantioselectivity from chiral HPLC measurements ... 52
     - 5.1.6. Time course of the cleavage of (*R,R*)-4 and (*S,S*)-4 ... 52
     - 5.1.7. Michaelis-Menten kinetics for (*R*,*R*)-1 ... 55
     - 5.1.8. Michaelis-Menten kinetics for the individual enantiomers of diol 4 ... 55
     - 5.1.9. Double-mutant cycle analysis ... 56

5.1.10. Calibration curves for the quantification of substrates and products from HPLC peaks 57
6. Crystallization and X-ray structure determination of PLZ2.3 59
7. Chemical methods 61
7.1. Separation of (*R,R*)-/(*S,S*)-4 from *meso*-4 61
7.2. Chemical syntheses 61
7.2.1. Synthesis of 1,4-diphenylbutane-2,3-diol (4) 61
8. HPLC standards of used compounds 63
9. HPLC chromatograms of the substrate scope / yield determination 66
10. HPLC chromatograms of the selectivity for substrate 1 70
11. HPLC chromatograms of the selectivity for substrate 4 72
12. References 75

1. Supporting figures

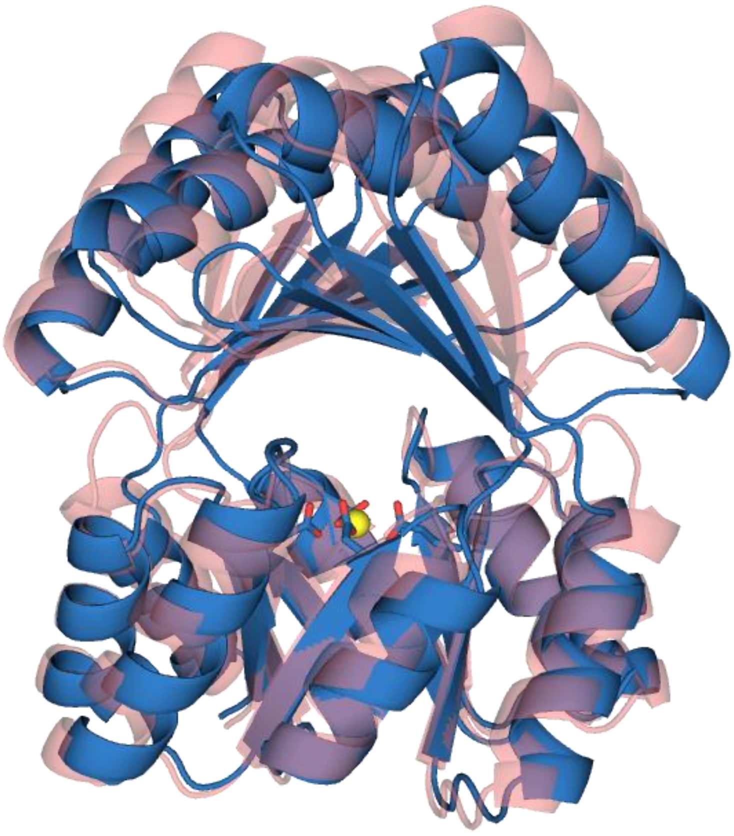

```
          1                  20                  40                  60
PLZ1.4  MGDILIVAAKNVDEMLKQVEILRRLGAKQIAVESSDWRILQEALKKGGDILIVFGGGMTITFRGDDLEALLKAAI
        M DILIV   + D  L QV  LR  GA+ IAVE +D  +L  A+  G DIL+V G  ++I  + D +E LL
PLZ2.0  MADILIVQDLDPDAKLAQVRELRAAGARIIAVEDADADLLIRAMDLGADILVVLGRKVSI--KSDTVEQLLATVR

              80                  100                 120                 140
PLZ1.4  EMIKQALKFGATITLSLDGNDLNINITGVPEQVRKELAKQAERLAKEFGITVTRTGGGNVDEMLKQVEILRRLGA
          +++A + G  I + +  N++ I     PEQV + +A      +KE G+ +         + L+ +  LR  GA
PLZ2.0  FAMERAHELGLDIWIVVKDNNIYIFFASAPEQVAQFVAALTA-FSKEQGLEIKVID----QDPLENIRRLREYGA

                   160                 180                 200                 220
PLZ1.4  KQIAVESNDWRILQEALKKGGDILIVAAKNVDEMLKQVEILRRLGAKQIAVESSDWRILQEALKKGGDILIVAGG
        K IA E ++   L  AL+ G DILIV A +++  ++ + LR  GAK IAVES++   L+ AL+ G DILI+ G
PLZ2.0  KIIAYEDDNADRLIRALEAGADILIVQAADIEATVEAIRRLREAGAKIIAVESANLEQLKAALELGADILIIQGR

                        240                 260                 280
PLZ1.4  GMTITFRGDDLEALLKAAIEMIKQALKFGATITLSLDGNDLNINITGVPEQVRKELAKQAERLAKEFG--ITVTR
         + +  R D  +  ++ A+ ++K+A + G T+ L L  N L +     PEQ+  EL  Q   LA E G  I V
PLZ2.0  EVVV--RSDTFQEAIEVALFVVKKAWEAGVTVALRLRENTLRVIFAMTPEQL-AELIAQLRALAAEKGWEIRVFD

           300                 320
PLZ1.4  TGGGNVDEMLKQVEILRRLGAKQIAVESNDWRILQEALKKG
        T      + L  +  LR LGAK IA+ES D  +L   L+
PLZ2.0  T------DPLAAMRELRELGAKIIALESPDLDVLLAGLRAA
```

Figure S1: Alignment of PLZ1.4 and PLZ2.0. Top: Structural alignment. AlphaFold predictions of PLZ1.4 and PLZ2.0 are shown red in blue, respectively. Lanthanide-coordinating residues are shown as sticks, the cerium ion is shown as a yellow sphere. Bottom: Sequence alignment using the NCBI Blast tool (https://blast.ncbi.nlm.nih.gov/Blast.cgi). Lanthanide-coordinating Glu residues are marked in red, inter-domain linkers in blue. Numbering is according to PLZ1.4.

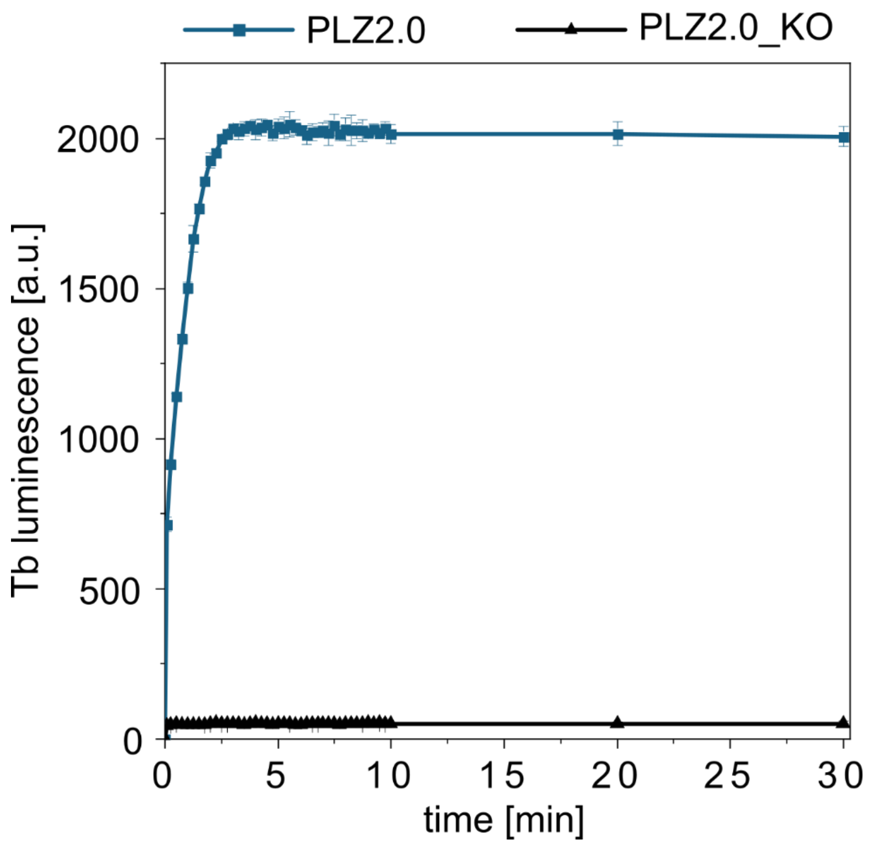

Figure S2: Time course of Tb(III) binding to PLZ2.0. Measurement of tryptophan-enhanced Tb(III) luminescence (excitation: 280 nm, emission: 544 nm) at 21 °C demonstrates complete metal binding after 5 min for PLZ2.0. Final concentrations of proteins and $TbCl_3$: 10 µM.

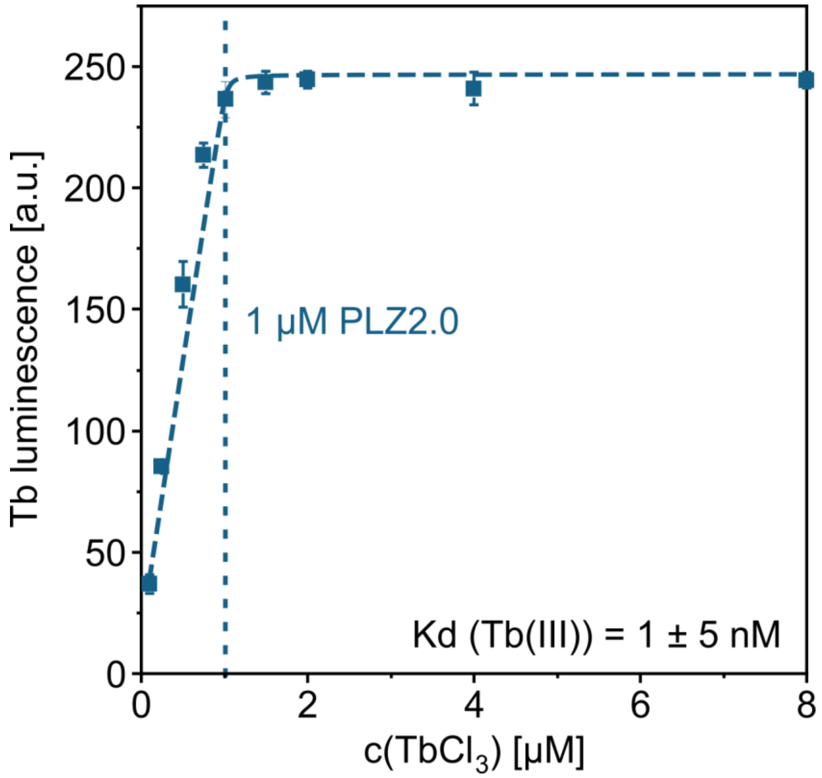

Figure S3: Titration of $TbCl_3$ to PLZ2.0 at pH 8.5. Increasing amounts of Tb(III) were added to 1 µM PLZ2.0. Tryptophan-enhanced Tb(III) luminescence (excitation: 280 nm, emission: 544 nm) was used as the readout. The resulting curve was analyzed using a quadratic fit that returned a K_d of 1 ± 5 nM. The curve shape and the large error indicate that the K_d is too low to be determined precisely in this experiments, but is estimated in the nanomolar range. Furthermore, the experiment confirms the 1:1 stoichiometry.

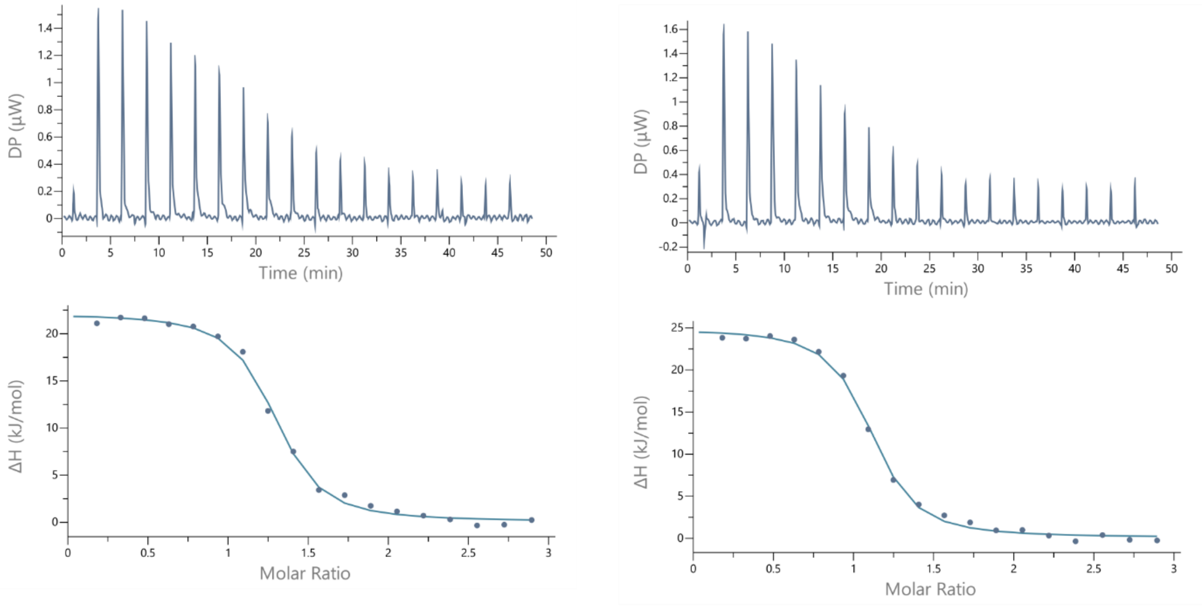

pH = 7.0	1st replicate	2nd replicate	average
N (sites)	1.23 ± 0.01	1.05 ± 0.01	1.14
K_d ($CeCl_3$) [nM]	329 ± 56	357 ± 52	343
ΔH [kJ/mol]	22.0 ± 0.4	24.8 ± 0.4	23.4
ΔG [kJ/mol]	-37.0	-36.8	-36.9
-TΔS [kJ/mol]	-59.1	-61.6	-60.4

Figure S4: ITC measurements of PLZ2.3 and $CeCl_3$ at pH 7.0. 20 µM PLZ2.3 was titrated with freshly prepared 300 µM $CeCl_3$ in identical buffer (25 mM HEPES, 100 mM NaCl, pH 7.0), with injection volumes of 1x 0.4 µL and 18x 2 µL. Baseline and offset of the thermograms were subtracted and differential enthalpy values were fitted. Results are derived from two independent measurements. The determined stoichiometry N, affinity K_d, and thermodynamic values ΔH, ΔG, and -TΔS are shown in the table. The average was calculated from both replicates.

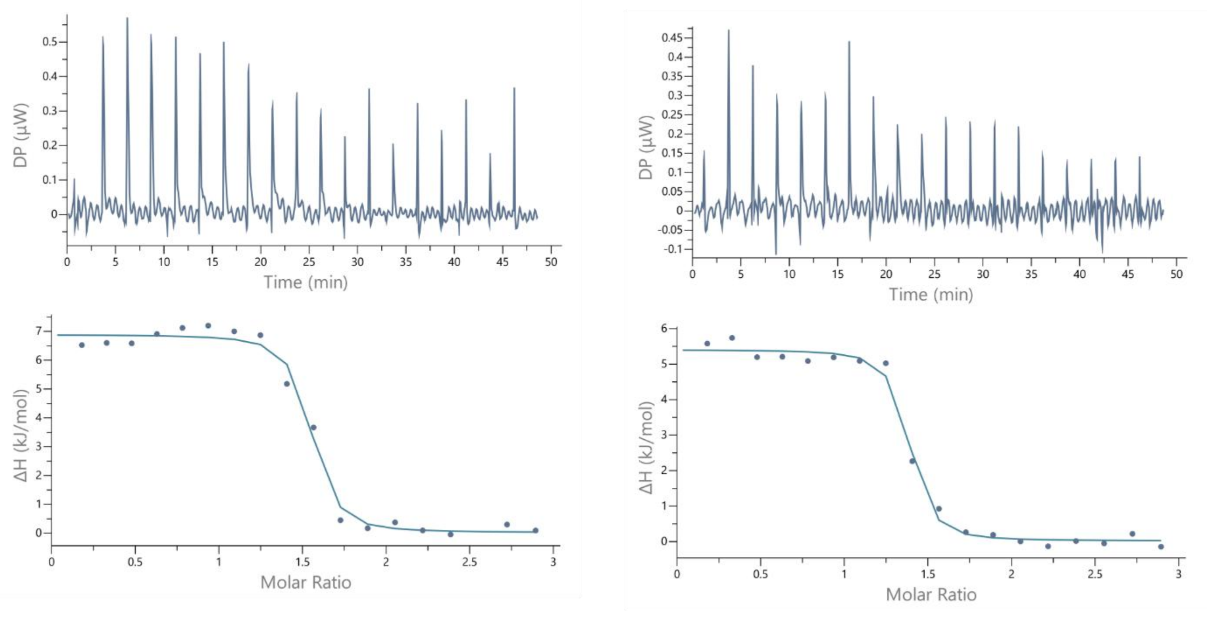

pH = 7.5	1st replicate	2nd replicate	average
N (sites)	1.48 ± 0.02	1.31 ± 0.01	1.40
K_d ($CeCl_3$) [nM]	61 ± 30	56 ± 23	59
ΔH [kJ/mol]	6.9 ± 0.2	5.4 ± 0.1	6.2
ΔG [kJ/mol]	-41.2	-41.4	-41.3
-TΔS [kJ/mol]	-48.1	-46.8	-47.5

Figure S5: ITC measurements of PLZ2.3 and $CeCl_3$ at pH 7.5. 20 µM PLZ2.3 was titrated with freshly prepared 300 µM $CeCl_3$ in identical buffer (25 mM HEPES, 100 mM NaCl, pH 7.5), with injection volumes of 1x 0.4 µL and 18x 2 µL. Baseline and offset of the thermograms were subtracted and differential enthalpy values were fitted. Results are derived from two independent measurements. The determined stoichiometry N, affinity K_d, and thermodynamic values ΔH, ΔG, and -TΔS are shown in the table. The average was calculated from both replicates.

a

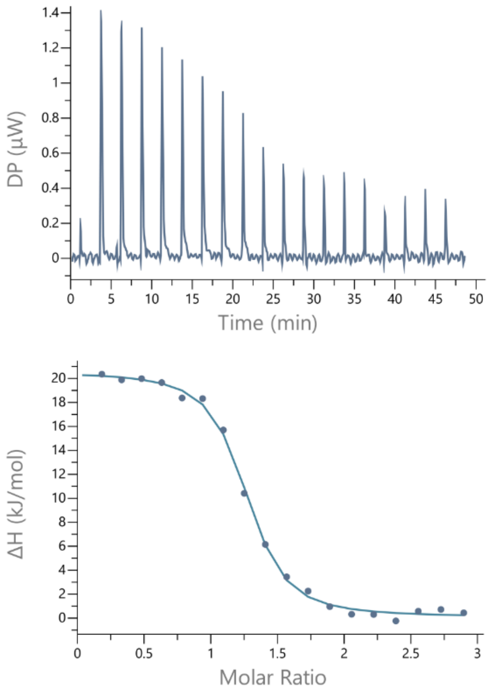

b

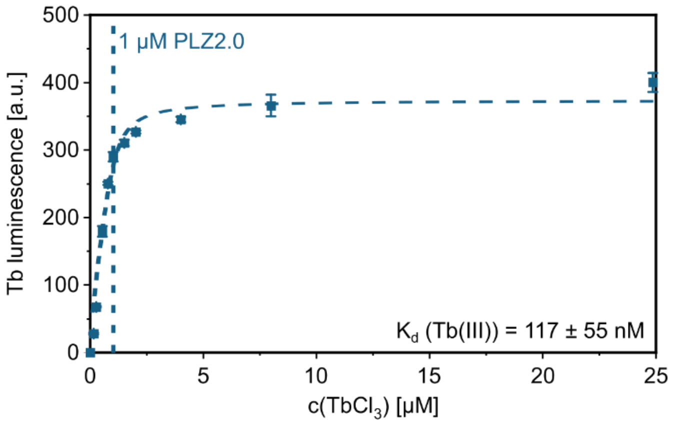

pH = 7.0	ITC	titration
N (sites)	1.20 ± 0.01	-
K_d ($TbCl_3$) [nM]	349 ± 49	117 ± 55
ΔH [kJ/mol]	20.5 ± 0.3	-
ΔG [kJ/mol]	-36.9	-
-TΔS [kJ/mol]	-57.4	-

Figure S6: Tb(III) binding to PLZ2 variants at pH 7.0 using complementary binding assays. (a) ITC of PLZ2.3 and $TbCl_3$ at pH 7.0. 20 µM PLZ2.3 was titrated with freshly prepared 300 µM $TbCl_3$ in identical buffer (25 mM HEPES, 100 mM NaCl, pH 7.0), with injection volumes of 1x 0.4 µL and 18x 2 µL. Baseline and offset of the thermograms were subtracted and differential enthalpy values were fitted. The determined stoichiometry N, affinity K_d, and thermodynamic values ΔH, ΔG, and -TΔS are shown in the table. (b) Titration of $TbCl_3$ to PLZ2.0 at pH 7.0. Tryptophan-enhanced Tb(III) luminescence (excitation: 280 nm, emission: 544 nm) was used as the readout. Increasing amounts of Tb(III) were added to 1 µM PLZ2.0. The resulting curve was analyzed using a quadratic fit that returned a K_d of 117 ± 55 nM.

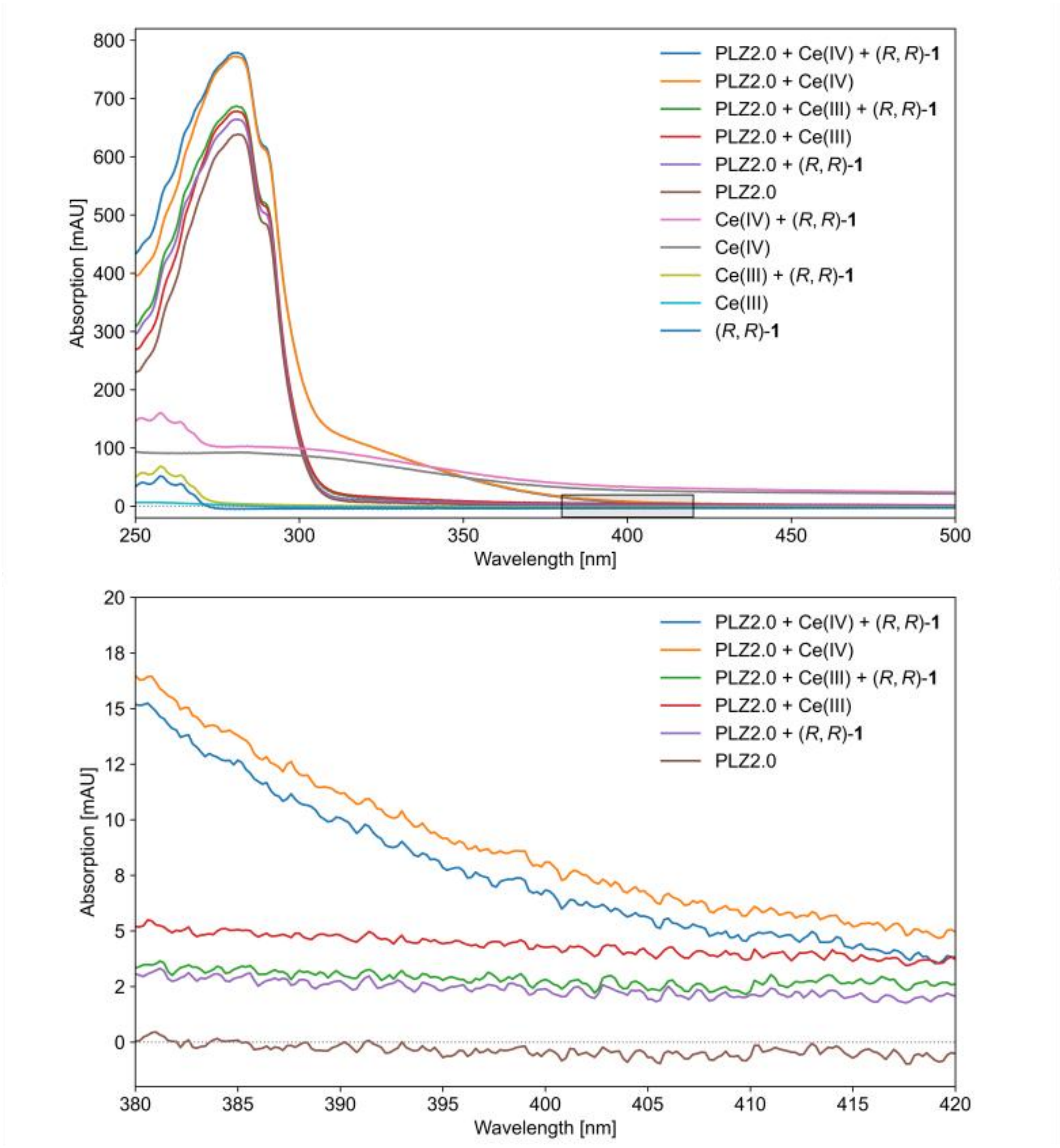

Figure S7: UV/Vis absorption studies. Measurements were performed with 30 µM PLZ2.0 and/or 30 µM cerium salt ($CeCl_3$ and $(NH_4)_2[Ce(NO_3)_6]$, respectively) with 10% acetonitrile as co-solvent. If indicated, substrate (*R*,*R*)-**1** was added at fivefold molar excess. mAU = milli absorbance unit.

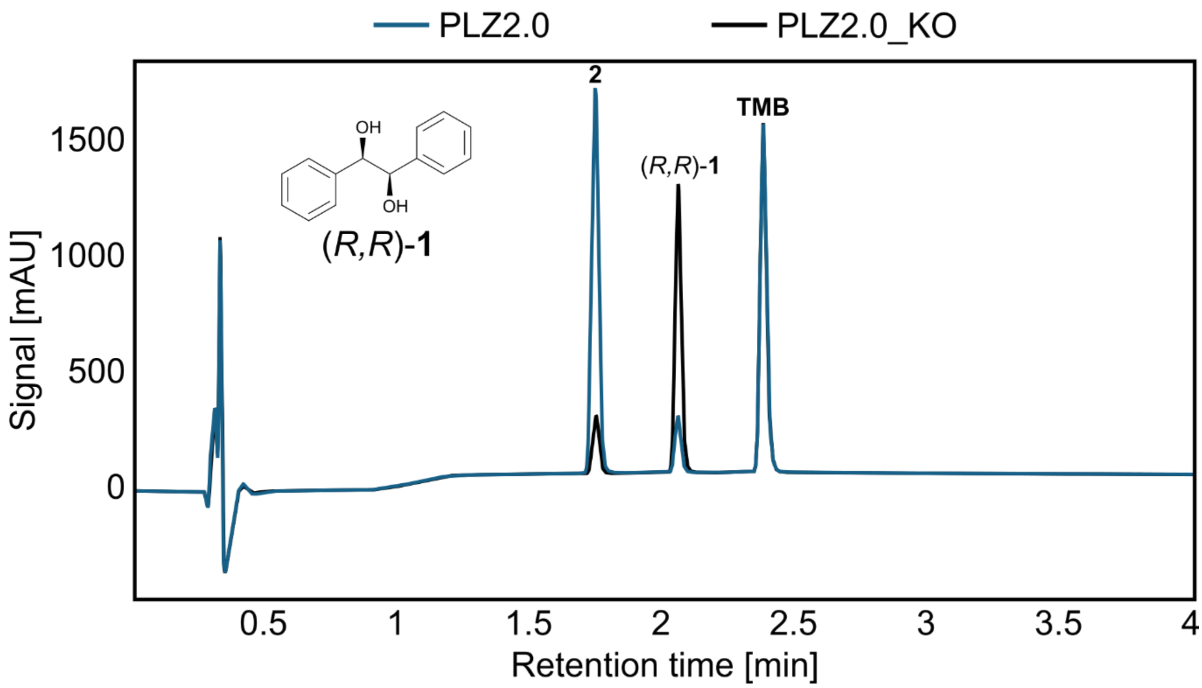

**Enzyme**	**Compound**	**Retention time [min]**	**Wavelength [nm]**	**Area [mAU*min]**	**Calculated yield**
PLZ2.0	Benzaldehyde **2**	1.738	254	52.2519	**76.4%**
	(*R*,*R*)-Hydrobenzoin (*R*,*R*)-**1**	2.050	205	6.5922	
	1,3,5-Trimethoxybenzene (**TMB**)	2.372	205	43.0883	
PLZ2.0_KO	Benzaldehyde **2**	1.741	254	8.1099	**9.9%**
	(*R*,*R*)-Hydrobenzoin (*R*,*R*)-**1**	2.051	205	33.6180	
	1,3,5-Trimethoxybenzene (**TMB**)	2.372	205	43.3073	

Figure S8: Comparison of catalytic activity between PLZ2.0 and PLZ2.0_KO. Irradiation of substrate (*R,R*)-**1** for 24 h with 1 mol% catalyst loading yielded 76.4% product **2** for PLZ2.0 and 9.9% product **2** for PLZ2.0_KO.

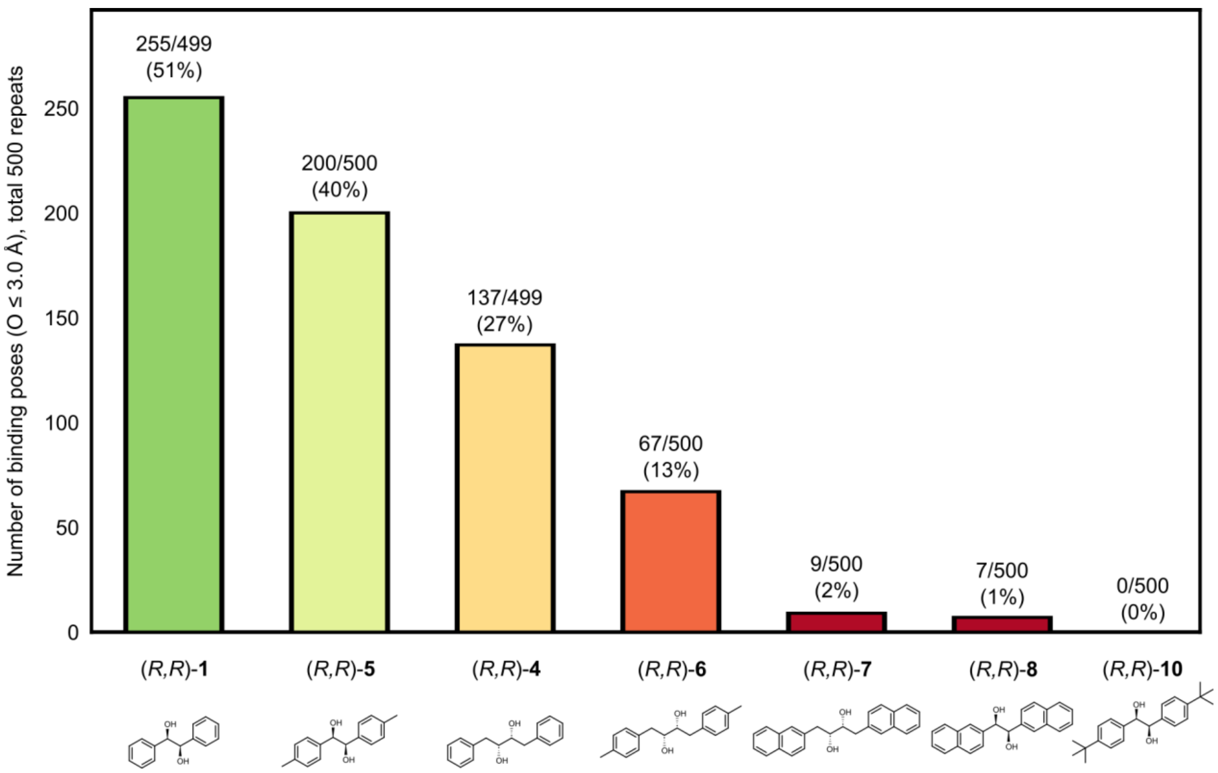

Figure S9: Blind docking of diol substrates with increasing size using Glide. 500 poses of each substrate were blindly docked into PLZ2.3 without defining a binding site. "Binding poses" were defined as poses where the distance between the Ce atom and one of the substrate oxygen atoms was below a threshold of 3 Å. The plot represents the percentage of binding poses out of all poses for each substrate.

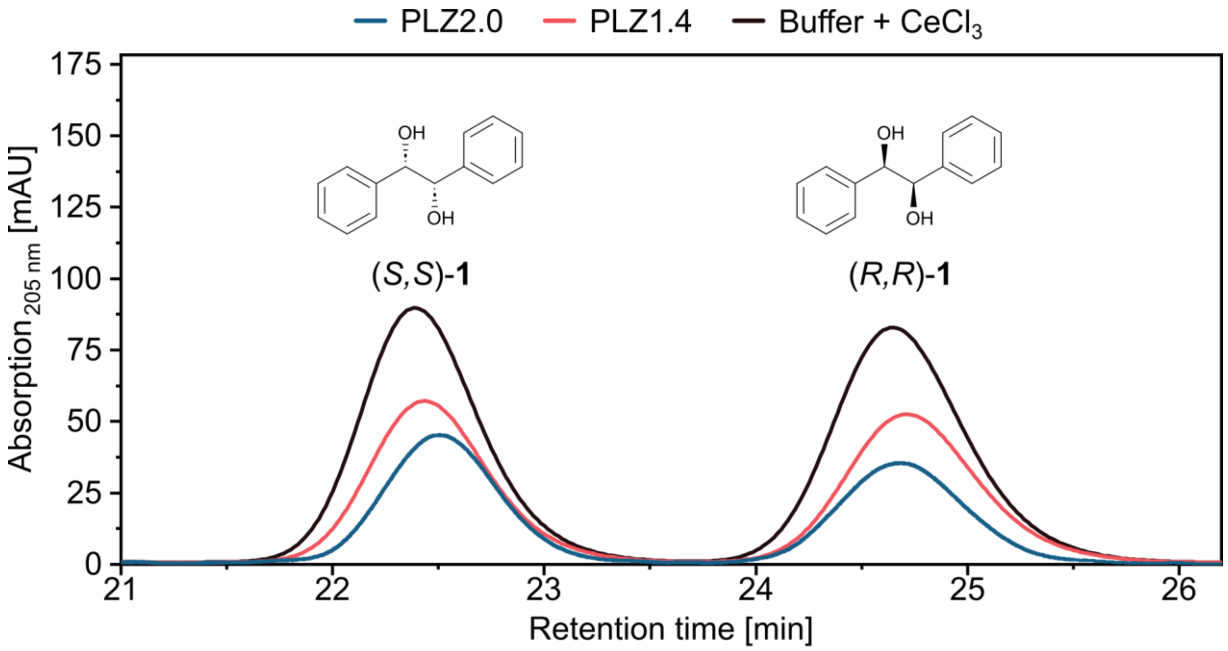

	**PLZ1.4** (5 mol%) 15 h irradiation	**PLZ2.0** (1 mol%) 24 h irradiation
ee [%]	0	4
conversion [%]	39	65
S value	1.00	1.08

Figure S10: Enantioselectivity of PLZ1.4 and PLZ2.0 towards substrate **1**. The selectivity is not as pronounced as for substrate **4**, which was instead used for directed evolution.

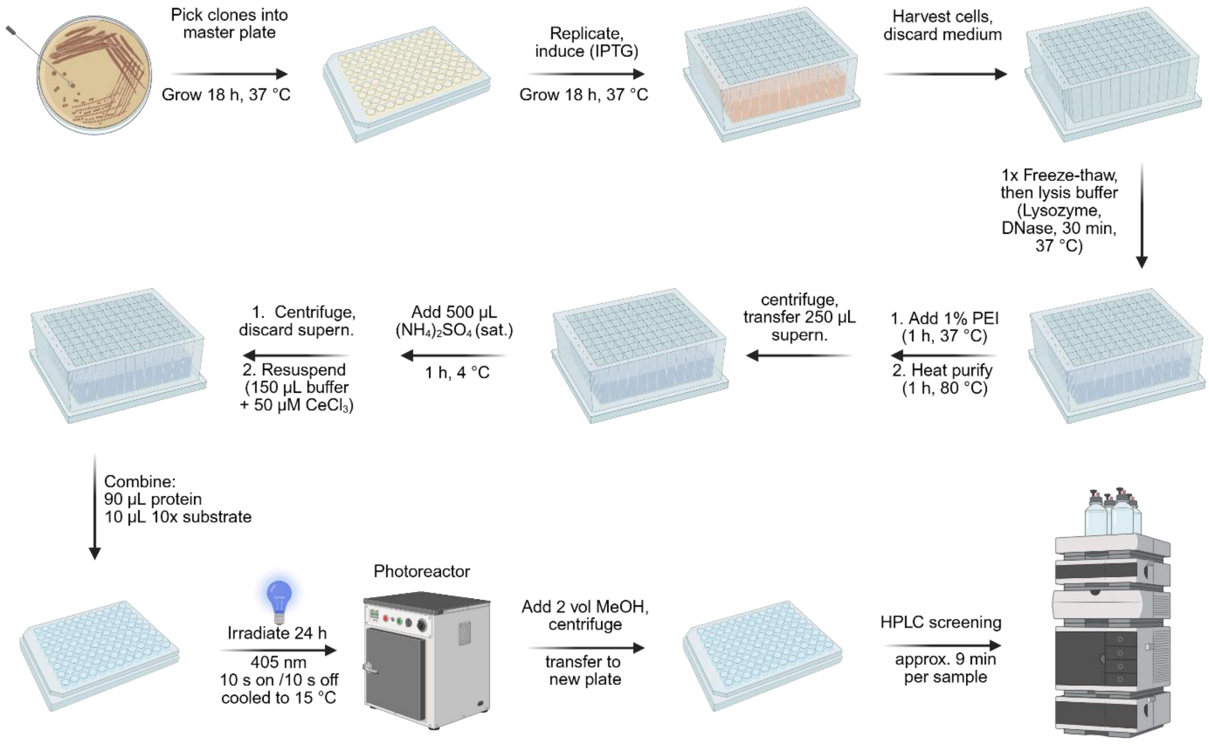

Figure S11: Workflow for the plate-based purification, cerium loading, and photocatalytic activity assay of PLZ proteins. Created in BioRender. Leiß-Maier, F. (2025) https://BioRender.com/q71g642

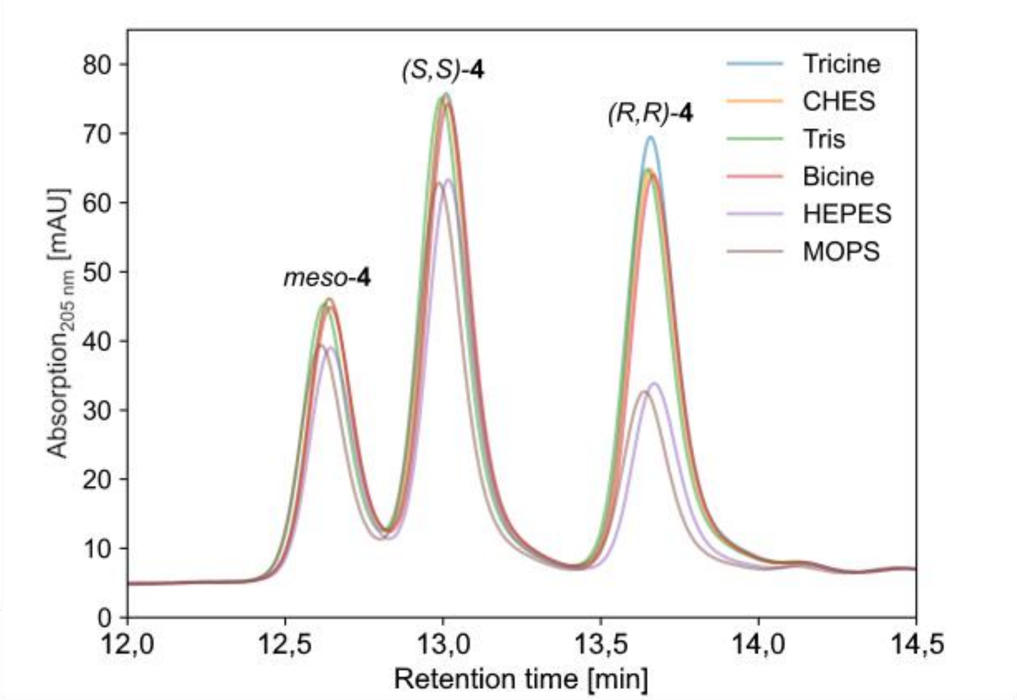

	Tricine	CHES	Tris	Bicine	HEPES	MOPS
ee [%]	3 ± 0	6 ± 0	6 ± 1	6 ± 0	37 ± 4	38 ± 1
conversion [%]	13 ± 1	16 ± 2	20 ± 8	18 ± 0	49 ± 5	48 ± 1

Figure S12: Buffer screen. Enantiomeric excess and conversion of the photocatalytic diol cleavage were measured in different buffers for PLZ2.3 and substrate **4** under standard conditions. The buffer solutions contained 25 mM of the respective buffer component and 100 mM NaCl at pH 8.5. HEPES and MOPS gave similar results, while reactions in all other tested buffers showed significantly reduced activity. As Good's buffers contain different functional groups (alcohol, amine, carboxylic acid, sulfonic acid), they may not be fully innocent in the reaction. However, chemically simpler phosphate or carbonate buffers were also unsuitable, as they form insoluble lanthanide salts, thereby impairing the cerium-dependent reaction.

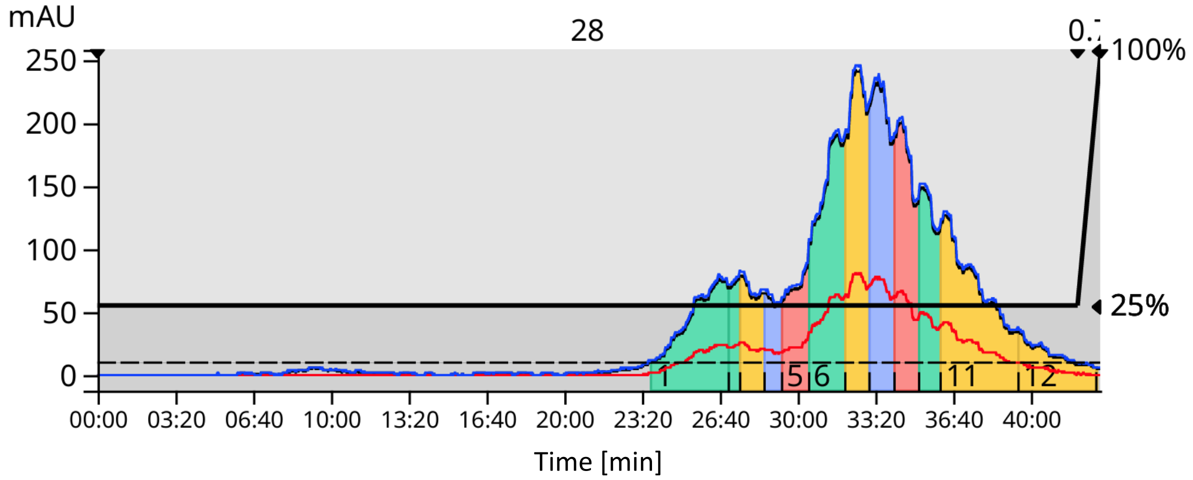

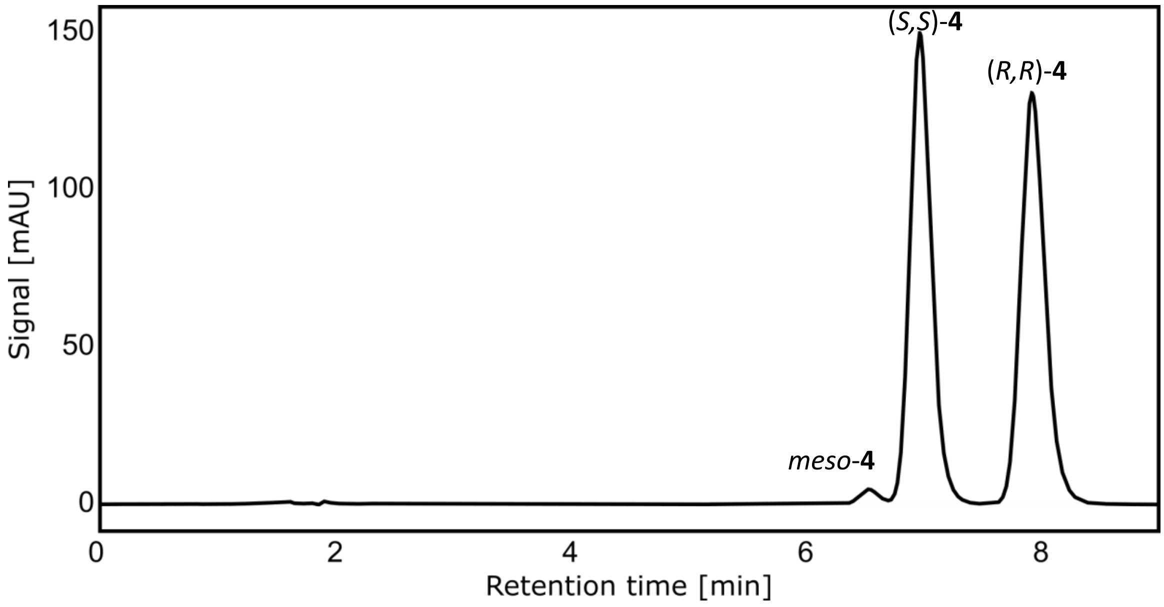

Figure S13: Separation of *meso*-**4** from (*S,S*)-**4** and (*R,R*)-**4**. Top: Biotage® Selekt report of the separation. Bottom: Chiral HPLC analysis of the pooled fractions 6-12.

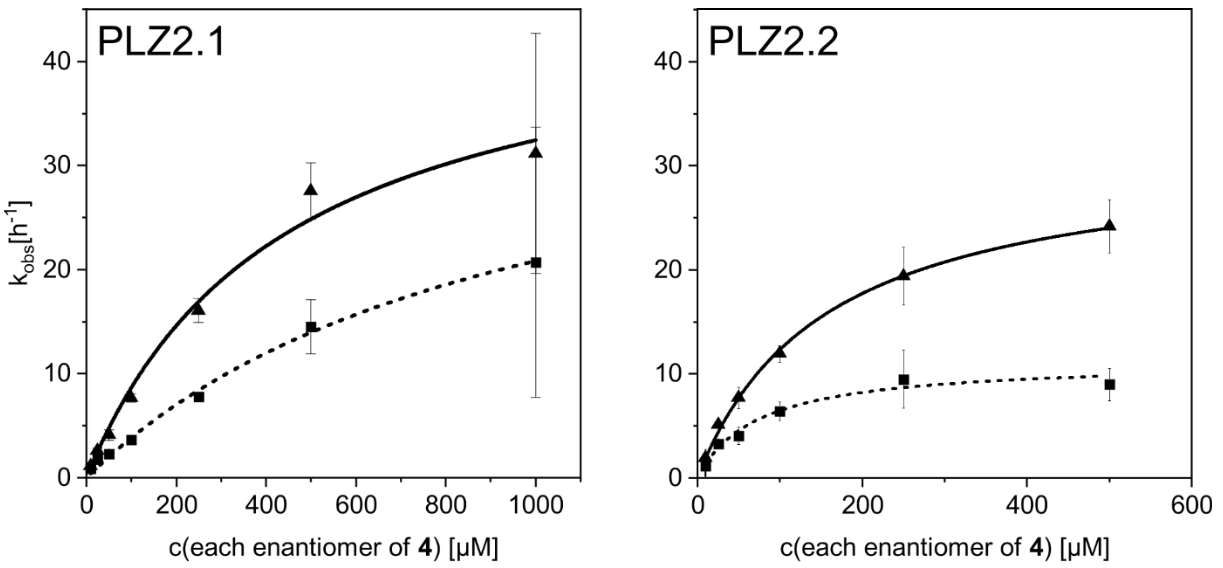

	PLZ2.1			PLZ2.2		
	k_{cat} [h^{-1}]	K_M [µM]	k_{cat}/K_M [M^{-1}s^{-1}]	k_{cat} [h^{-1}]	K_M [µM]	k_{cat}/K_M [M^{-1}s^{-1}]
(*R*,*R*)-**4**	46.7 ± 10.2	439 ± 150	29.5	31.4 ± 2.0	156 ± 23	55.9
(*S*,*S*)-**4**	41.0 ± 19	968 ± 759	11.8	11.2 ± 1.2	74 ± 22	42.0

Figure S14: Michaelis-Menten kinetics of the single mutants PLZ2.1 (left) and PLZ2.2 (right) determined for the individual enantiomers of substrate **4.**

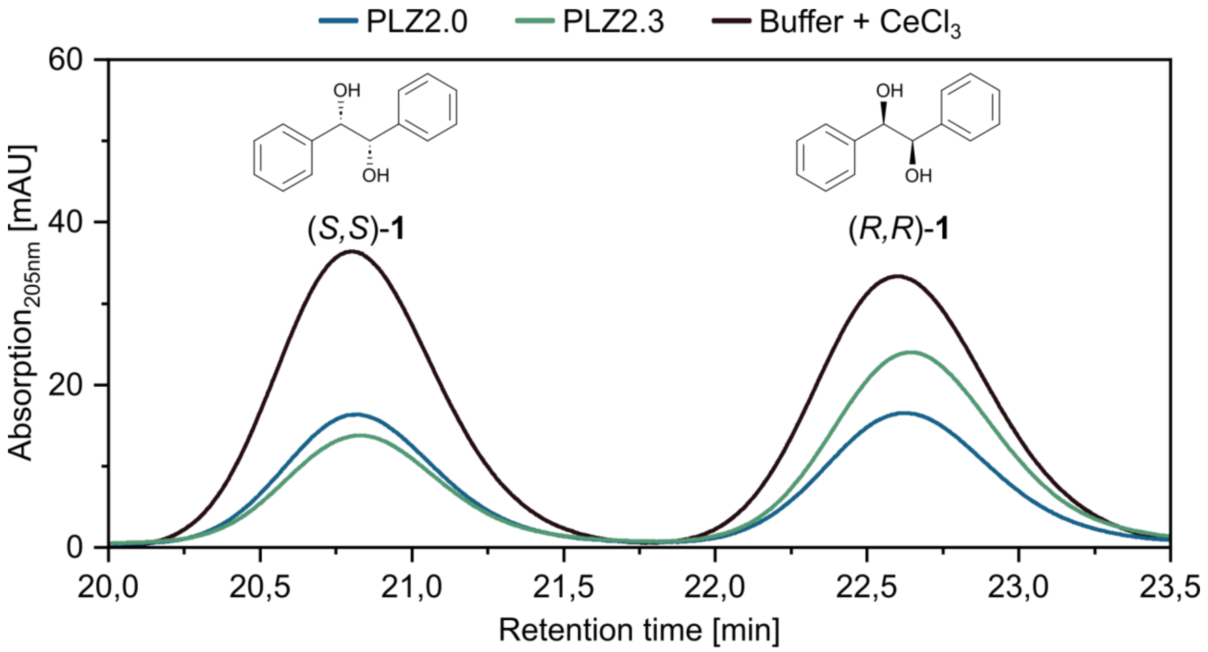

	PLZ2.0	PLZ2.3
ee [%]	5	23
conversion [%]	50	42
S value	1.14	2.39

18 h irradiation time

Figure S15: Enantioselectivity of PLZ2.3 towards substrate **1**. Interestingly, the (*S,S*)-enantiomer is consumed preferably, while for the very similar but slightly larger substrate **4**, the (*R,R*)-enantiomer is preferred.

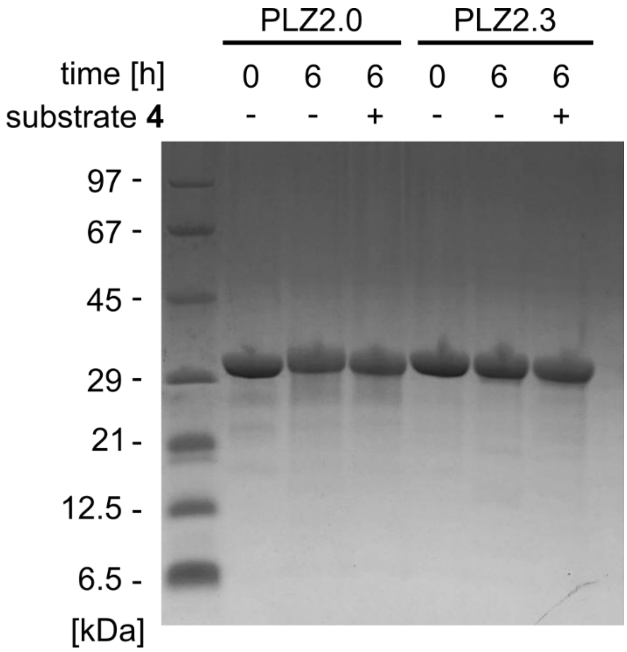

Figure S16: SDS-PAGE analysis of the potential photodamage to PLZ2.0 and PLZ2.3 after 6 h of irradiation, compared to the non-irradiated sample (t = 0). The absence of additional bands indicates no significant protein crosslinking or degradation.

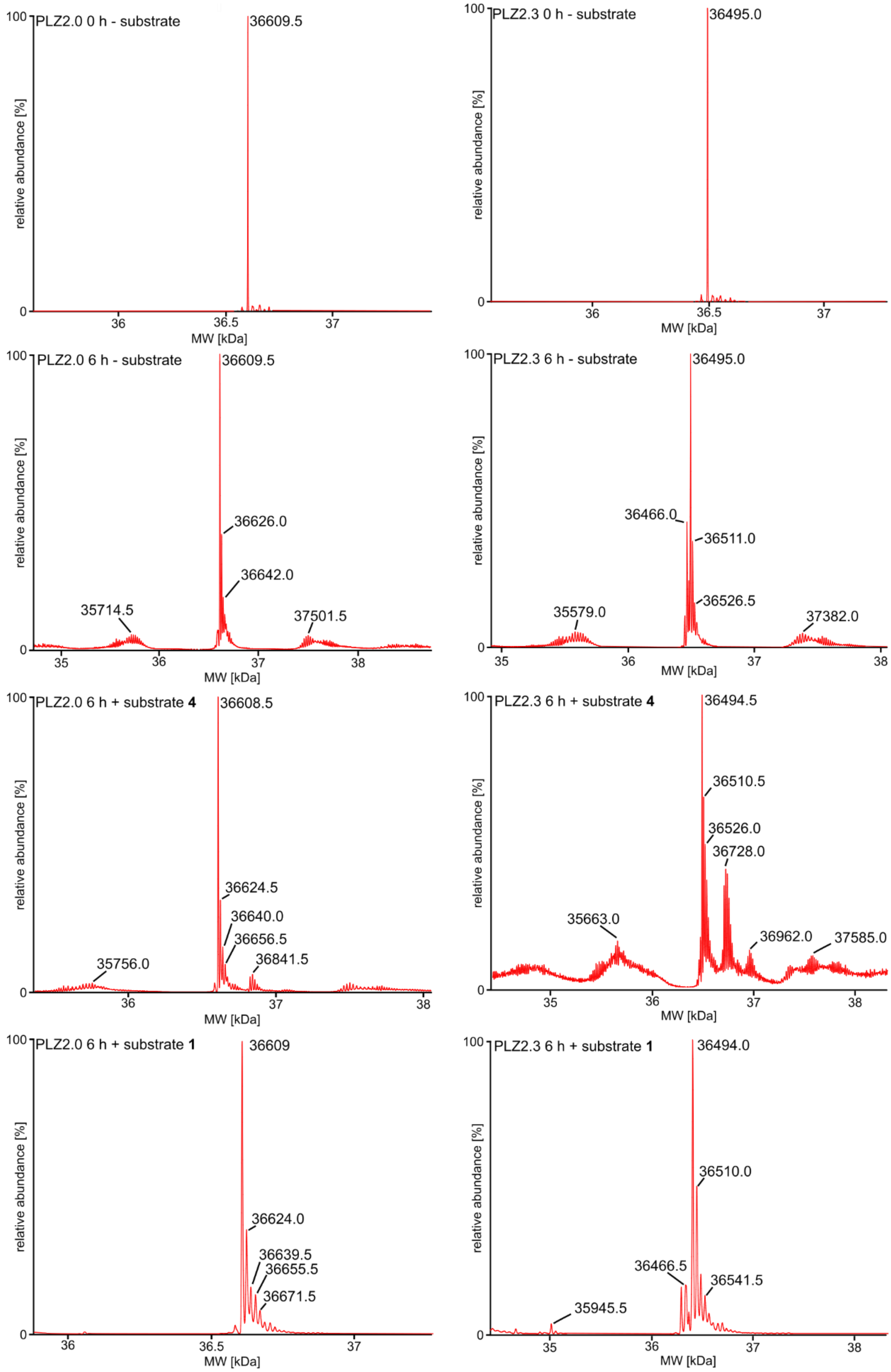

Figure S17: LC-MS analysis PLZ2.0 (left) and PLZ2.3 (right) before irradiation and after 6 h of irradiation in the absence and presence of substrate **4** and substrate **1**. In the irradiated samples, +16 Da, +32 Da,… peaks are visible, indicating single oxidation events. For the irradiated samples in the presence of substrate **4**, additional peaks are visible, potentially arising from adduct formation. The modifications are more pronounced for PLZ2.3, in line with its higher catalytic activity. Still, the non-modified species is the most prominent for all samples.

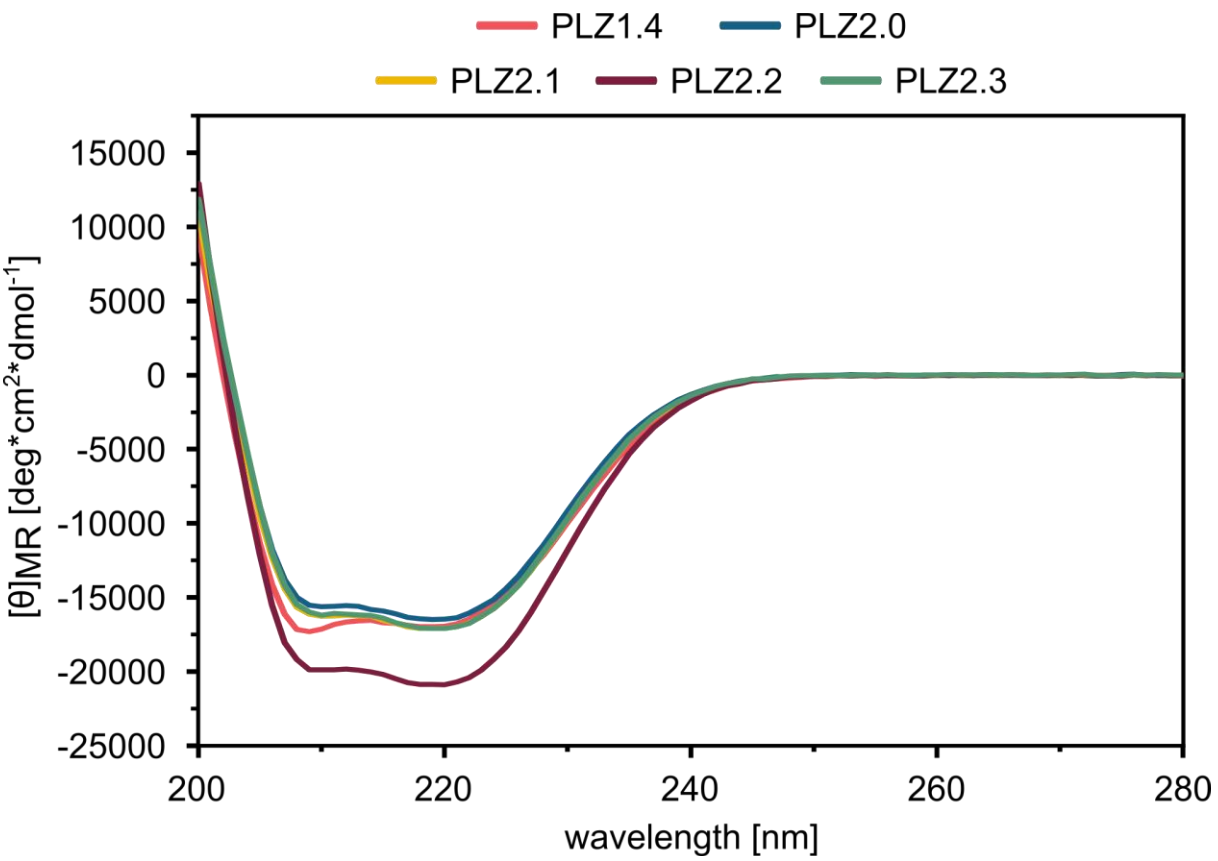

Figure S18: Circular dichroism spectra of all photoenzyme variants. The shape similarity indicates highly similar folds between the structures, as verified by the crystal structure of PLZ2.3. $[\Theta]_{MR}$: Mean residue molar ellipticity.

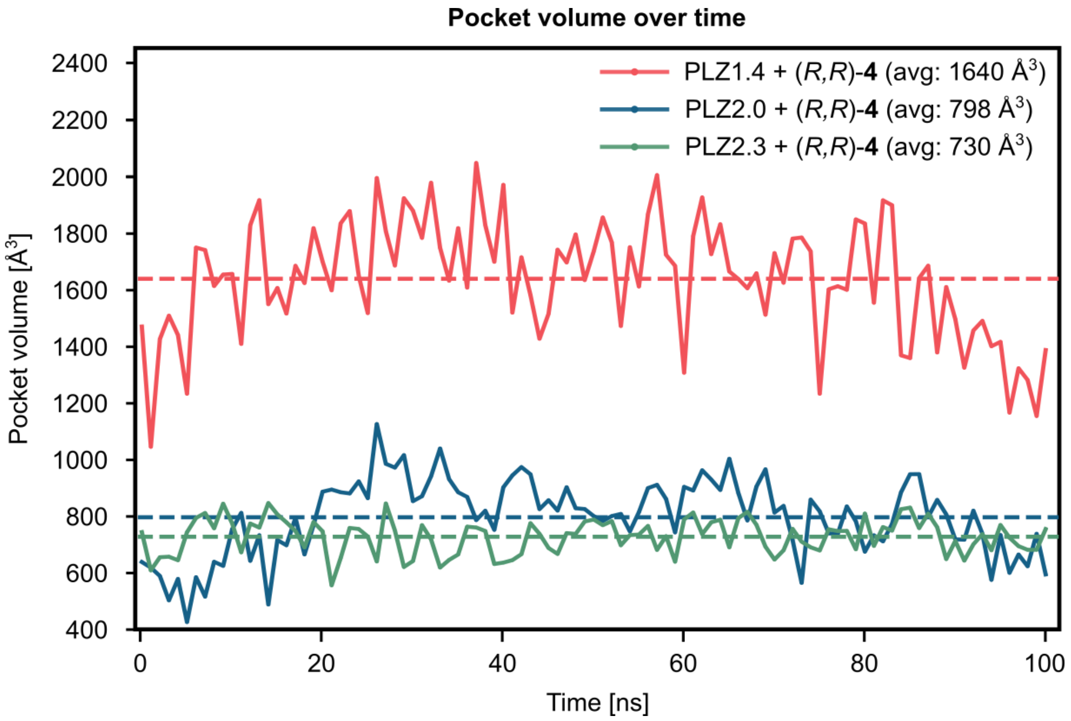

Figure S19: Analysis of the binding pocket volume of PLZ1.4, PLZ2.0, and PLZ2.3 over the course of the MD simulation.

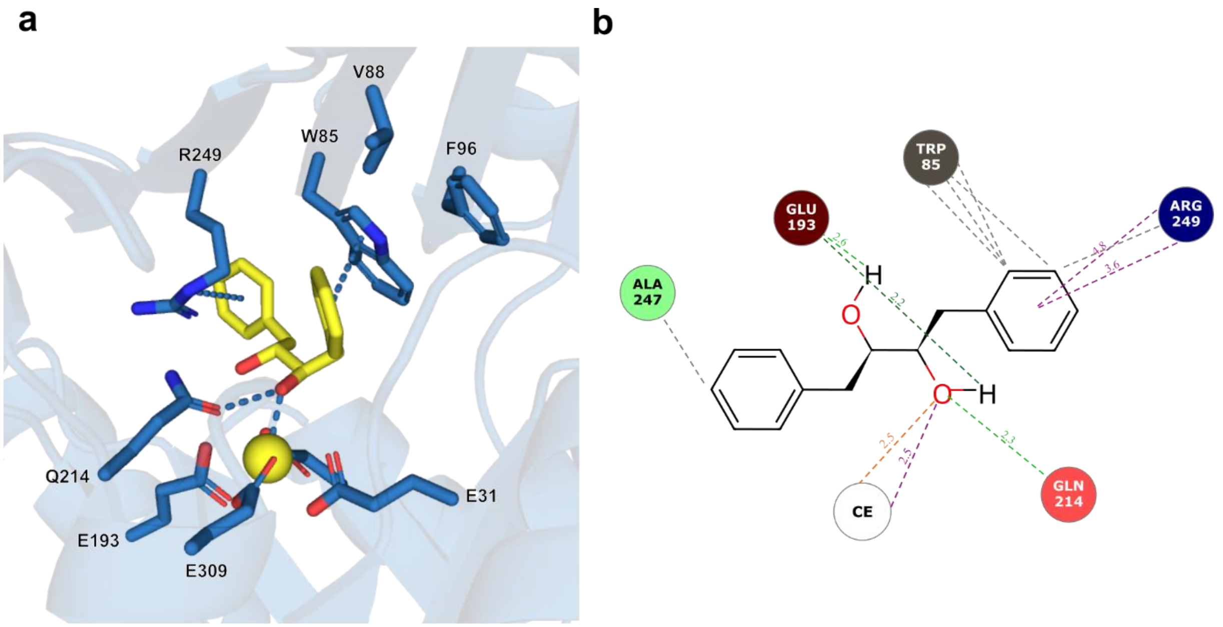

Figure S20: Key interactions of PLZ2.0 with (*R,R*)-**4** in a representative MD frame (frame 969). (a) 3D representation. (b) 2D representation.

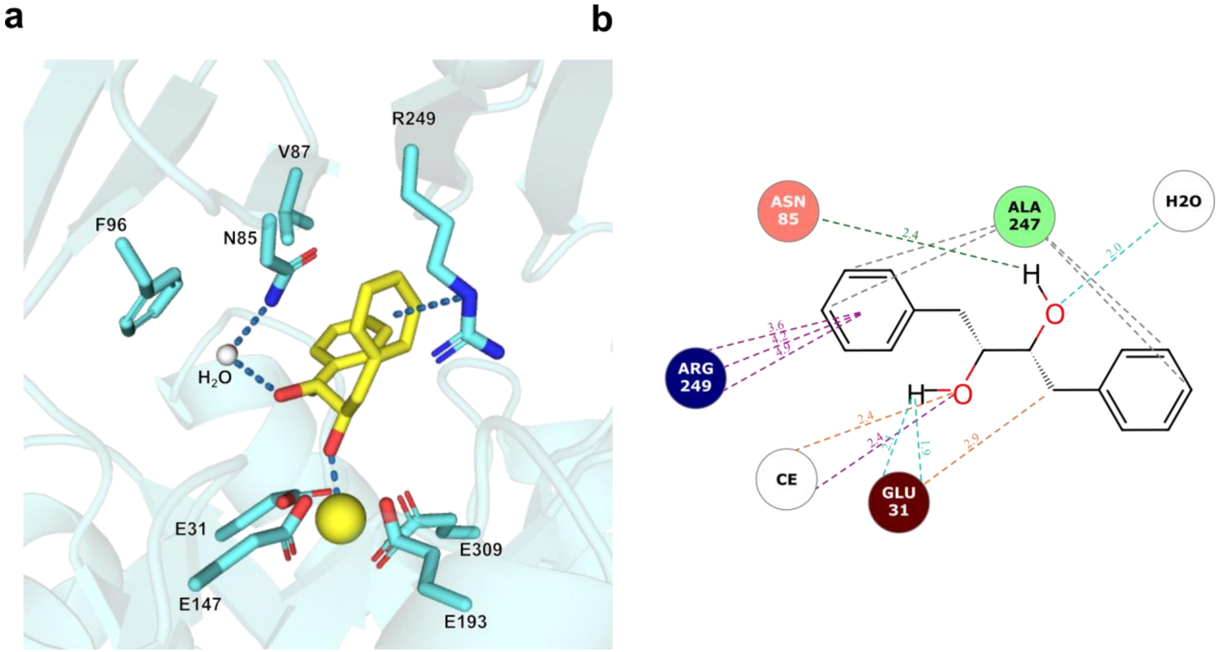

Figure S21: Key interactions of PLZ2.1 with (*R,R*)-**4** in a representative MD frame (frame 232). (a) 3D representation. (b) 2D representation.

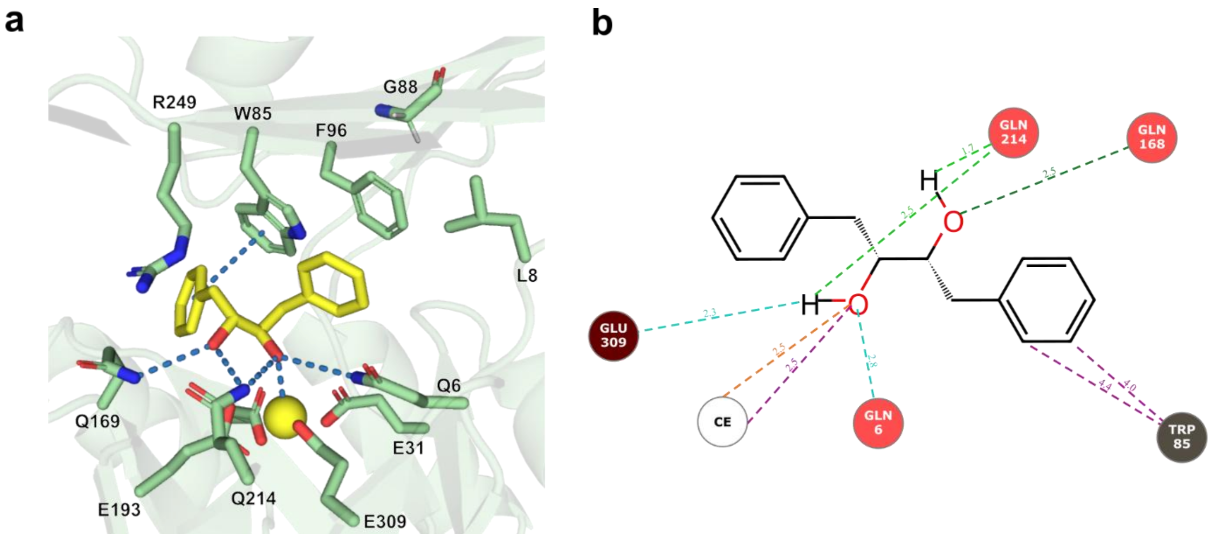

Figure S22: Key interactions of PLZ2.2 with (*R,R*)-**4** in a representative MD frame (frame 775). (a) 3D representation. (b) 2D representation

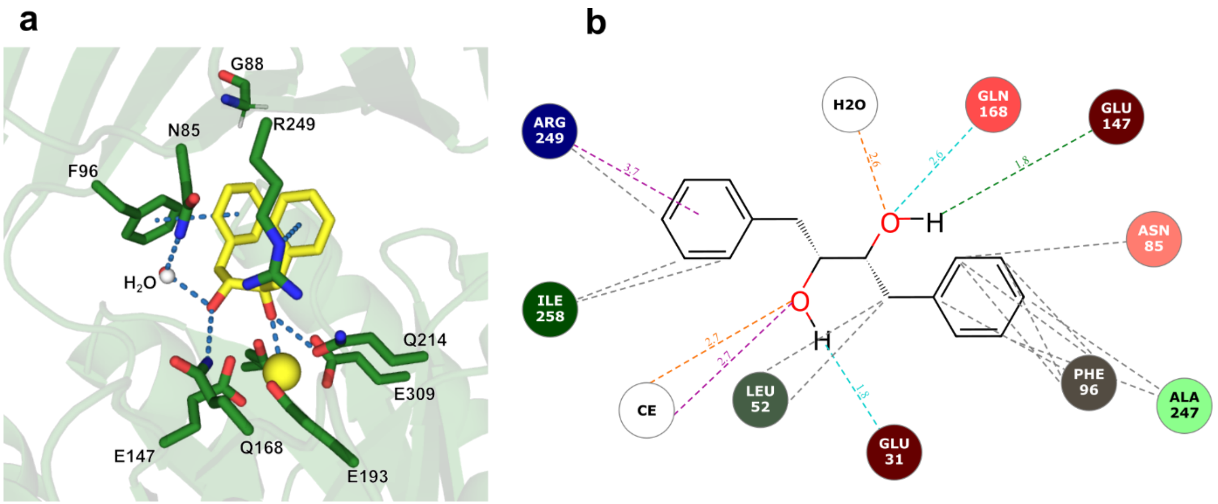

Figure S23: Key interactions of PLZ2.3 with (*R,R*)-**4** in a representative MD frame (frame 334). (a) 3D representation. (b) 2D representation

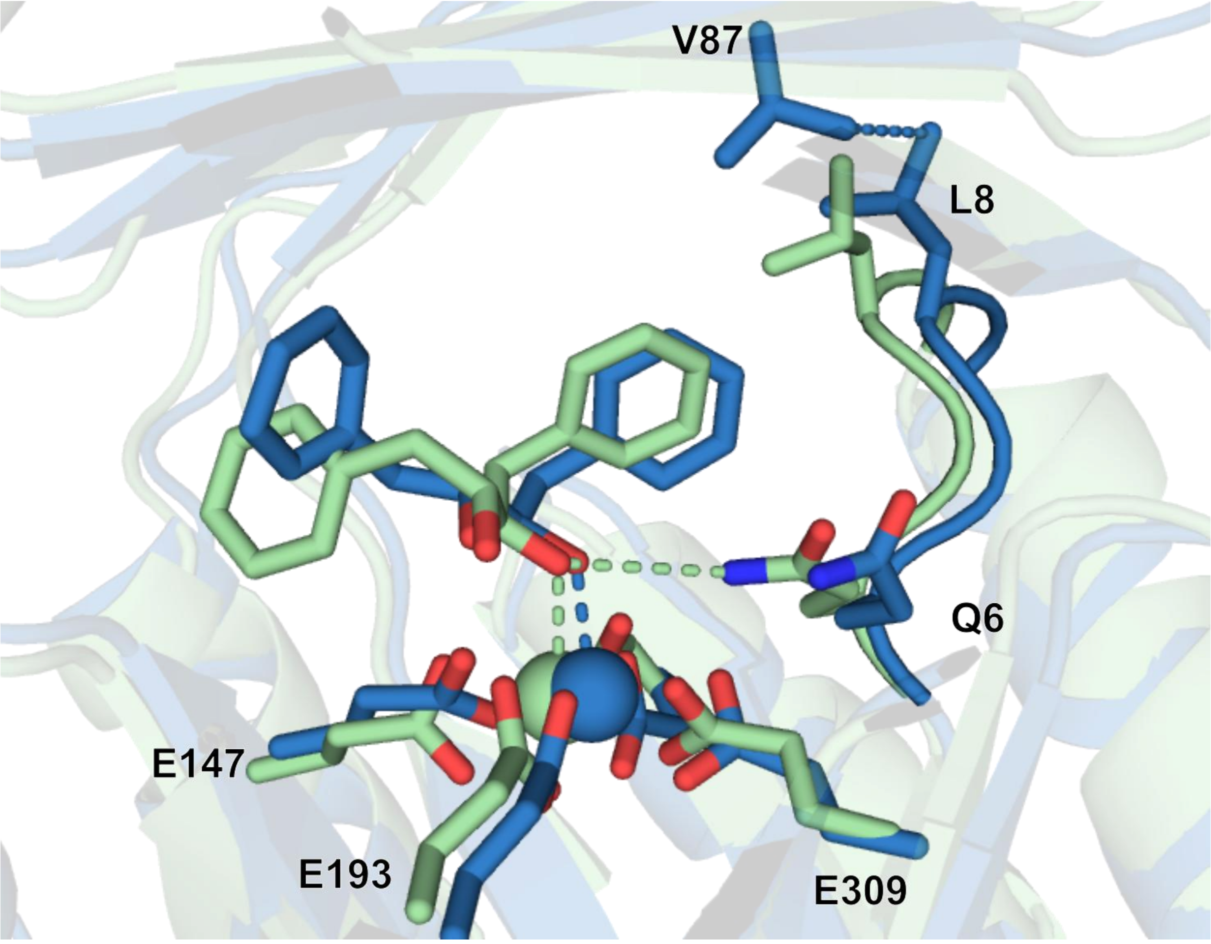

Figure S24: MD-based comparison between PLZ2.0 (blue) and PLZ2.2 (pale green), suggesting that the V87G mutation alters a loop conformation. This may facilitate the formation of a stabilizing hydrogen bond between Q6 and (*R,R*)-**4**.

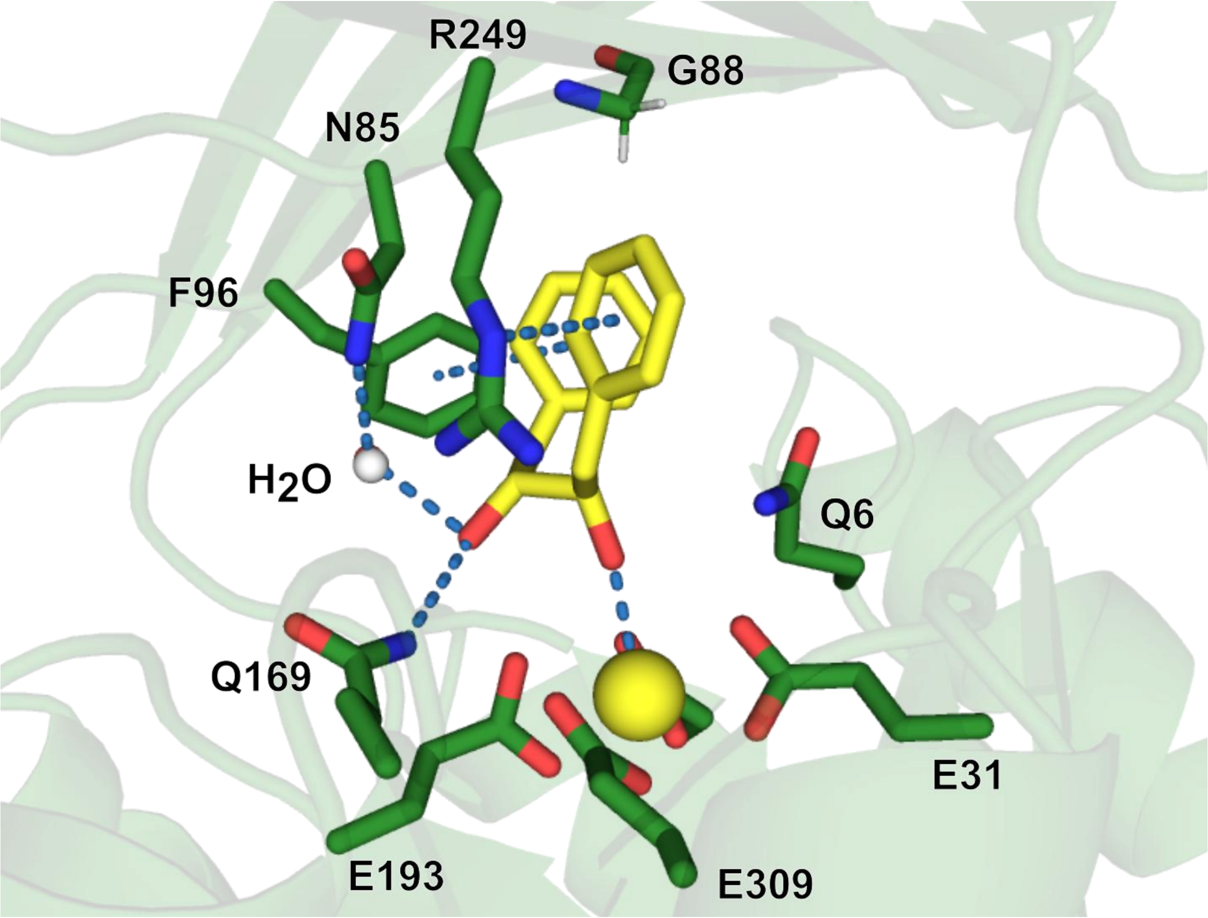

Figure S25: The MD-based analysis of PLZ2.3 bound to (*R,R*)-**4** provides a potential explanation for antagonistic epistasis. Q6 and Q169/N85 reside on opposite sides of the cavity, therefore being mutually exclusive for substrate stabilization. The substrate can only form hydrogen bonds with either side, indicating why the beneficial effects of PLZ2.1 and PLZ2.2 are not additive.

2. General methods

Chemical synthesis: Unless stated otherwise, commercially available substances were used without further purification. (*S,S*)-**1** was purified by sublimation. Reactions requiring an inert atmosphere were performed under Schlenk conditions in dry nitrogen. Solvents were removed at 40 °C *in vacuo*. Reactions were analyzed *via* thin-layer chromatography (TLC) on pre-coated plastic sheets (Polygram SIL G/UV254, Macherey–Nagel, Düren, Germany), and the compounds were visualized by irradiation with an UV lamp. Products were purified using silica gel 60 (particle size 0.040–0.063 mm, 230–240 mesh, Macherey–Nagel, Düren, Germany).

NMR spectroscopy: ^{1}H and ^{13}C NMR spectra were recorded at room temperature on a Bruker Avance III HD 400 or a Bruker Avance III HD 500 nuclear magnetic resonance spectrometer (Bruker, Billerica, USA) in $CDCl_3$ or DMSO-d_6. Splitting patterns are given as singlet (s), doublet (d), triplet (t), quartett (q), doublet of doublet (dd), and multiplet (m). Coupling constants (J) are reported in Hz.

HPLC analysis: Reactions were analyzed on a Vanquish System (Thermo Fisher Scientific, Waltham, MA, USA) equipped with an achiral Hypersil Gold C18 column (100 x 2.1 mm, 3 µm particle size, Thermo Fisher Scientific, Waltham, MA, USA) at 45 °C and a chiral Chiralcel OJ-RH column (150 x 4.6 mm, 5 µm particle size, Daicel Corporation, Osaka, Japan) at room temperature. Molecules were detected at 205 nm and 254 nm. For achiral measurements, water + 0.1% (v/v) trifluoroacetic acid (TFA) and acetonitrile + 0.1% (v/v) TFA were used as solvents. For chiral measurements, pure water and acetonitrile were used as solvents.

<u>Method 1 (achiral Hypersil Gold C18 column):</u> Water + 0.1% (*v*/*v*) trifluoroacetic acid (TFA) as solvent "A" and acetonitrile + 0.1% (*v*/*v*) TFA as solvent "B" were used as eluents for the following gradient program:

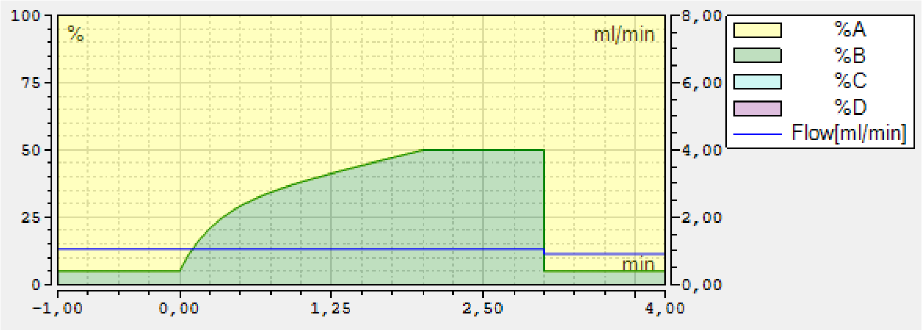

No	Time	Flow [ml/min]	%B	%C	%D	Curve
1	-1,000	Equilibration				
2	-1,000	1,050	5,0	0,0	0,0	5
3	New Row					
4	0,000	Run				
5	0,000	1,050	5,0	0,0	0,0	5
6	2,000	1,050	50,0	0,0	0,0	3
7	3,000	1,050	50,0	0,0	0,0	5
8	3,000	0,900	5,0	0,0	0,0	5
9	4,000	0,900	5,0	0,0	0,0	5
10	New Row					
11	4,000	Stop Run				

Method 2 (chiral Chiralcel OJ-RH column): Water as solvent “C” and acetonitrile as solvent “D” were used as eluents for the following gradient program:

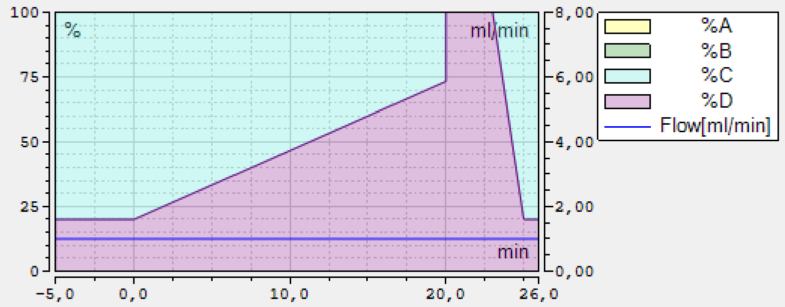

No	Time	Flow [ml/min]	%B	%C	%D	Curve
1	-5,000	Equilibration				
2	-5,000	1,000	0,0	80,0	20,0	5
3	New Row					
4	0,000	Run				
5	0,000	1,000	0,0	80,0	20,0	5
6	20,000	1,000	0,0	26,7	73,3	5
7	20,000	1,000	0,0	0,0	100,0	5
8	23,000	1,000	0,0	0,0	100,0	5
9	25,000	1,000	0,0	80,0	20,0	5
10	26,000	1,000	0,0	80,0	20,0	5
11	New Row					
12	26,000	Stop Run				

Method 3 (chiral Chiralcel OJ-RH column): Water as solvent “C” and acetonitrile as solvent “D” were used as eluents for the following program:

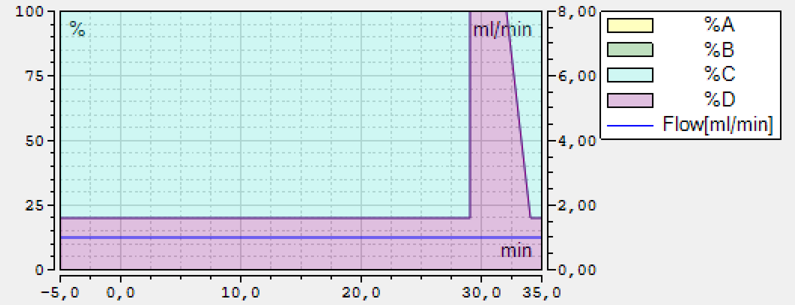

No	Time	Flow [ml/min]	%B	%C	%D	Curve
1	-5,000	Equilibration				
2	-5,000	1,000	0,0	80,0	20,0	5
3	New Row					
4	0,000	Run				
5	0,000	1,000	0,0	80,0	20,0	5
6	29,000	1,000	0,0	80,0	20,0	5
7	29,000	1,000	0,0	0,0	100,0	5
8	32,000	1,000	0,0	0,0	100,0	5
9	34,000	1,000	0,0	80,0	20,0	5
10	35,000	1,000	0,0	80,0	20,0	5
11	New Row					
12	35,000	Stop Run				

Method 4 (chiral Chiralcel OJ-RH column): Water as solvent "C" and acetonitrile as solvent "D" were used as eluents for the following program:

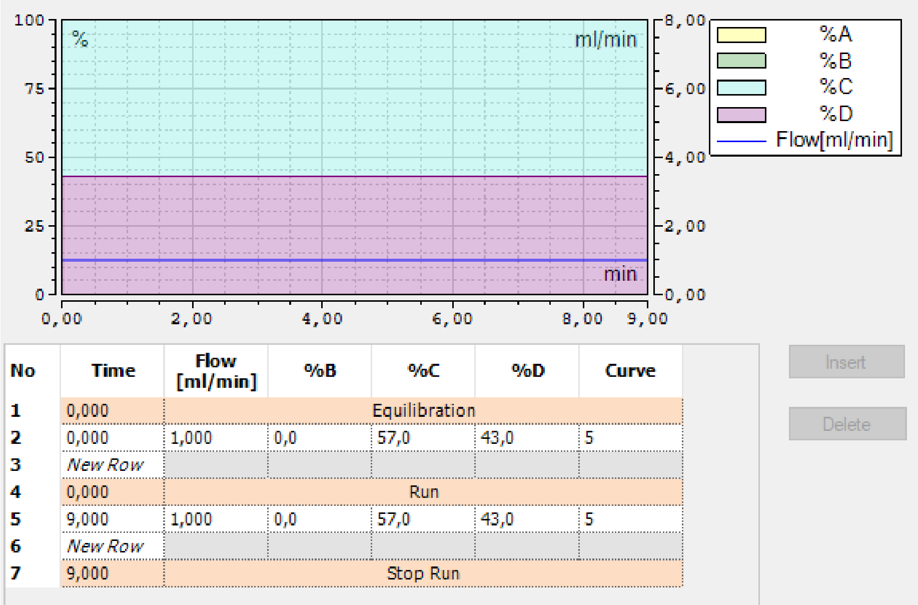

No	Time	Flow [ml/min]	%B	%C	%D	Curve
1	0,000	Equilibration				
2	0,000	1,000	0,0	57,0	43,0	5
3	*New Row*					
4	0,000	Run				
5	9,000	1,000	0,0	57,0	43,0	5
6	*New Row*					
7	9,000	Stop Run				

3. Biochemical methods

3.1. Molecular cloning

Synthetic genes were ordered from *Twist Bioscience* (South San Francisco, CA, USA). All PCR reactions were performed as described in Table S1 and Table S2. Vectors were assembled using Gibson assembly (1 h, 50 °C)[1] or site-directed, ligase-independent mutagenesis (SLIM; 2 cycles, 3 min 95 °C, 5 min 65 °C, 40 min 30 °C).[2] Chemically competent *E. coli* DH10B cells were transformed with the assembled DNA by heat shock (42 °C, 45 s) and rescued by adding 750 µL SOC medium (20 g/L tryptone, 5 g/L yeast extract, 584 mg/L sodium chloride, 186 mg/L potassium chloride, 2 g/L magnesium chloride hexahydrate, 1204 mg/L magnesium sulfate, 3.6 g/L glucose). After outgrowth for 1 h at 37 °C, cells were plated on agar plates or cultivated in LB media supplied with the respective antibiotics and grown at 37 °C. Sequences were verified by *Sanger* sequencing (*GENEWIZ Germany* GmbH, Leipzig, Germany).

Table S1: Composition of PCR reactions

Component	Amount
dNTPs (10 mM)	1 µL
Template DNA (3–50 ng/µL)	1 µL
Primer fw (10 mM)	2.5 µL
Primer rv (10 mM)	2.5 µL
Q5 buffer (5x)	10 µL
Q5 High-Fidelity polymerase	1 µL
ad aqua dest.	to 50 µL

Table S2: General PCR program

Step	Temp. [°C]	Duration	Cycle
Initial denaturation	98	1 min	
Denaturation	98	10 s	
Annealing	Primer dep.	30 s	
Elongation	72	20–30 s/kbp	35x
Final elongation	72	10 min	

3.1.1. Generation of site-saturation libraries for directed evolution

Site-saturation libraries were generated using Gibson assembly with overlapping primers. To introduce the desired mutations, a short primer was designed to bind directly upstream of the target codon, facing outward (with its 5’ end adjacent to the codon to be modified). A second, longer primer was designed in the opposite orientation to enable amplification of the entire vector. This longer primer spanned the target codon and replaced it with a degenerate codon, while also including at least 20 base pairs (bp) of sequence complementary to the region amplified by the short primer. During PCR, this design resulted in the production of a linear, double-stranded DNA fragment containing the degenerate codon and homologous regions on both ends. Gibson assembly then facilitated the seamless re-circularization of this fragment, generating a plasmid library with the desired site-saturation mutations. NDT codons were used as degenerate codons to limit the size of the produced library while also covering at least one representative of each class of amino acids.

3.1.2. Primers used in this study

Primer name	sequence (5' - 3')	comments
T7	TAATACGACTCACTATAGGG	sequencing
T7term	GCTAGTTATTGCTCAGCGG	sequencing
SL137 E31Q short for	CTGATGCCGACCTGCTG	Knock out binding site in PLZ2.0
SL138 E31Q short rev	CAATAATGCGAGCACCAGC	Knock out binding site in PLZ2.0
SL139 E31Q tailed for	CGGTTCAGGACGCTGATGCCGACCTGCTG	Knock out binding site in PLZ2.0
SL140 E31Q tailed rev	CGTCCTGAACCGCAATAATGCGAGCACCAGC	Knock out binding site in PLZ2.0
SL141 E147Q short for	TAACGCTGACCGTCTTATTC	Knock out binding site in PLZ2.0
SL142 E147Q short rev	CAATAATTTTTGCGCCATATTCTC	Knock out binding site in PLZ2.0
SL143 E147Q tailed for	CCTATCAGGACGATAACGCTGACCGTCTTATTC	Knock out binding site in PLZ2.0
SL144 E147Q tailed rev	TCGTCCTGATAGGCAATAATTTTTGCGCCATATTCTC	Knock out binding site in PLZ2.0

SL145 E193Q short for	TAACTTAGAACAACTGAAAGCTG	Knock out binding site in PLZ2.0
SL146 E193Q short rev	GCTATGATCTTAGCCCCTG	Knock out binding site in PLZ2.0
SL147 E193Q tailed for	CGTTCAGAGCGCTAACTTAGAACAACTGAAAGCTG	Knock out binding site in PLZ2.0
SL148 E193Q tailed rev	GCGCTCTGAACGGCTATGATCTTAGCCCCTG	Knock out binding site in PLZ2.0
SL149 E309Q short for	CGGATTTGGATGTATTACTTGC	Knock out binding site in PLZ2.0
SL150 E309Q short rev	CAATAATTTTTGCCCCCAGT	Knock out binding site in PLZ2.0
SL151 E309Q tailed for	CGTTACAGTCCCCGGATTTGGATGTATTACTTGC	Knock out binding site in PLZ2.0
SL152 E309Q tailed rev	GGGACTGTAACGCAATAATTTTTGCCCCCAGT	Knock out binding site in PLZ2.0
G1 pEt29 for	CCACCACCACCACTGAGATCCGGC	open pET29 vector for Gibson assembly
G2 pET29 rev	GAGGATGAGACCATGCACTAGCC	open pET29 vector for Gibson assembly
Lib9 L53 rev	TACTACAAGAATATCTGCGCCAA	NDT L53 in PLZ2.0
Lib10 L53 for	GGCGCAGATATTCTTGTAGTANDTGGCCGGAAAGTCTCC	NDT L53 in PLZ2.0
Lib11 W86 rev	GATATCCAGACCAAGCTCG	NDT W86 in PLZ2.0
Lib12 W86 for	CACGAGCTTGGTCTGGATATCNDTATCGTTGTAAAAGATAACAATATTTACATTTTC	NDT W86 in PLZ2.0
Lib13 V88 rev	GATCCAGATATCCAGACCAAG	NDT V88 in PLZ2.0

Lib14 V88 for	CTTGGTCTGGATATCTGGATCNDTGTAAAAGATAACAATATTTACATTTTCTTTGC	NDT V88 in PLZ2.0
Lib15 A99 rev	AAAGAAAATGTAAATATTGTTATCTTTTACAAC	NDT A99 in PLZ2.0
Lib16 A99 for	GTTGTAAAAGATAACAATATTTACATTTTCTTTNDTTCTGCTCCGGAGCAAG	NDT A99 in PLZ2.0
Lib17 Q169 rev	AACAATCAGAATGTCGGC	NDT Q169 in PLZ2.0
Lib18 Q169 for	GCGCCGACATTCTGATTGTTNDTGCAGCCGACATTGAGG	NDT Q169 in PLZ2.0
Lib19 A249 rev	AACGGTAACCCCCG	NDT A249 in PLZ2.0
Lib20 A249 for	GGGAAGCGGGGGTTACCGTTNDTCTGCGTTTACGAGAAAACAC	NDT A249 in PLZ2.0
Lib21 Q169 rev	GCCTCAATGTCGGCTGCTHNAACAATCAGAATGTCGGCGC	NDT Q169 in PLZ2.0
Lib22 Q169 for	GCAGCCGACATTGAGG	NDT Q169 in PLZ2.0
Lib23 Q169 rev	GCCTCAATGTCGGCTGCAHNAACAATCAGAATGTCGGCGC	NDT Q169 in PLZ2.0
Lib26 PLZ2.0 F97NDT rev	AATGTAAATATTGTTATCTTTTACAACGATCC	NDT F97 in PLZ2.0
Lib27 PLZ2.0 F97NDT for	CGTTGTAAAAGATAACAATATTTACATTNDTTTTGCATCTGCTCCGG	NDT F97 in PLZ2.0
Lib28 PLZ2.0 Q7NDT rev	CACGATCAATATGTCGGCC	NDT Q7 in PLZ2.0
Lib29 PLZ2.0 Q7NDT for	TGGCCGACATATTGATCGTGNDTGATCTTGACCCGGATGC	NDT Q7 in PLZ2.0
Lib30 PLZ2.0 L9NDT rev	ATCTTGCACGATCAATATGTCG	NDT L9 in PLZ2.0
Lib31 PLZ2.0 L9NDT for	ACATATTGATCGTGCAAGATNDTGACCCGGATGCTAAGC	NDT L9 in PLZ2.0
Lib32 PLZ2.0 I259NDT rev	AACGCGCAGCGTGTTTTC	NDT I259 in PLZ2.0
Lib33 PLZ2.0 I259NDT for	GAGAAAACACGCTGCGCGTTNDTTTTGCAATGACCCCGG	NDT I259 in PLZ2.0
Lib34 F97NDT for	CGGCGTAAAAGATAACAATATTTACATTNDTTTTGCGTCTGCTCCGG	NDT F97 in PLZ2.3
Lib35 F97NDT rev	AATGTAAATATTGTTATCTTTTACGCCG	NDT F97 in PLZ2.3

Lib36 A260NDT rev	AAATATAACGCGCAGCG	NDT A260 in PLZ2.3
Lib37 A260NDT for	ACACGCTGCGCGTTATATTTNDTATGACCCCGGAACAAC	NDT A260 in PLZ2.3
Lib38 M261NDT rev	TGCAAATATAACGCGCAG	NDT M261 in PLZ2.3
Lib39 M261NDT for	CGCTGCGCGTTATATTTGCANDTACCCCGGAACAACTG	NDT M261 in PLZ2.3
Lib40 G215NDT rev	CTGTATGATCAGTATATCCGCTC	NDT G215 in PLZ2.3
Lib41 G215NDT for	GGGAGCGGATATACTGATCATACAGNDTCGCGAAGTTGTCGTTC	NDT G215 in PLZ2.3
Lib42 E147NDT rev	ATAGGCAATAATTTTTGCGC	NDT E147 in PLZ2.3
Lib43 E147NDT for	GGCGCAAAAATTATTGCCTATNDTGACGATAACGCTGACC	NDT E147 in PLZ2.3
Lib44 A247NDT for	CTGCGTTTACGAGAAAACAC	NDT A247 in PLZ2.3
Lib45 A247NDT rev	GCGTGTTTTCTCGTAAACGCAGAHNAACGGTAACCCCCG	NDT A247 in PLZ2.3
Lib46 Q168NDT for	GCAGCCGACATTGAGG	NDT Q168 in PLZ2.3
Lib47 Q168NDT rev	GTGGCCTCAATGTCGGCTGCAHNAACAATCAGAATGTCGGCG	NDT Q168 in PLZ2.3

3.2. Expression, purification, and analysis of proteins

3.2.1. Culture conditions

Escherichia coli (*E. coli*) BL21-Gold(DE3) cells were grown in LB medium (*Carl Roth*, Karlsruhe, Germany: 10 g/L tryptone, 5 g/L yeast extract, 10 g/L sodium chloride) or on LB agar plates [1.5% (*w*/*v*) agar] at 37 °C. Ampicillin (final concentration of 100 µg/mL) or kanamycin (final concentration of 30 µg/mL) were added as antibiotics to the culture medium.

3.2.2. Buffers used for protein purification

Lysis buffer

25 mM HEPES, pH 8.5
100 mM NaCl
0.5 mg/mL Lysozyme
1 µg/mL DNaseI

Ni-NTA wash buffer

25 mM HEPES, pH 7.5
300 mM NaCl
30 mM Imidazole

Ni-NTA elution buffer

25 mM HEPES, pH 7.5
300 mM NaCl
300 mM Imidazole

FPLC buffer

25 mM HEPES, pH 8.5
100 mM NaCl

3.2.3. Protein expression and purification

An overnight pre-culture of *E. coli* BL21 DE3 supplied with the respective plasmid (pET29) was grown in 50 mL LB medium supplied with the respective antibiotics at 37 °C while shaking. The cells were diluted to an OD_{600} of 0.05 in 1.5 L LB + antibiotics and grown at 37 °C until an OD_{600} of 0.4 was reached. Expression was then induced by adding isopropyl-β-D-thiogalactopyranosid (IPTG) to a final concentration of 500 µM. After growing the cells for additional 16 h to allow for gene expression, they were harvested by centrifugation (25 min, 4,000 rcf). The pellet was then resuspended in 40 mL lysis buffer and subsequently lysed by sonication in a *Branson* SFX 500 sonifier (*Emerson Electric* Co., St. Louis, MO, USA; power-on time: 5 min, pulsed time: 5 s on, 5 s off, 35% power). The lysate was cleared by centrifugation (25 min, 11,000 rcf, 4 °C) and used for affinity chromatography.

The cleared lysate was loaded onto a column packed with 3 mL Ni-NTA agarose resin (*Qiagen* N.V., Venlo, Netherlands) equilibrated with 3–5 column volumes of Ni-NTA wash buffer. The resin was

washed with 5-10 CV Ni-NTA wash buffer before the protein was eluted with 3 CV Ni-NTA elution buffer. The elution fraction was concentrated using *Merck Millipore* Amicon Ultra centrifugal filter devices (*Merck* KGaA, Darmstadt, Germany; 10 kDa cut-off, 4000 rcf, 4 °C).

The protein was further purified by size-exclusion chromatography (SEC) using a NGC Quest 10 Plus Chromatography System (*Bio-Rad Laboratories* GmbH, Feldkirchen, Germany), equipped with a Superdex 75 Increase 10/300 GL column (*Cytiva*, Marlborough, MA, USA). Proteins were purified at room temperature using FPLC buffer. Fractions were analyzed by SDS-PAGE and fractions containing the protein of interest were pooled and further concentrated using Amicon centrifugal filter devices. Proteins were snap frozen in liquid nitrogen and stored in FPLC buffer at -70 °C. Protein concentrations were determined by UV absorption at 280 nm using calculated ε_{280} values.

3.2.4. Expression and purification of proteins in a 96-well format

E. coli BL21 cells were transformed with the library of choice and plated on LB agar plates with the appropriate antibiotic. After growing the colonies at 37 °C, individual clones were picked into a sterile 96-well microtiter plate filled with 120 µL LB medium with the respective antibiotic. This master plate was covered with Breathe-Easy membrane (Diversified Biotech, Dedham, MA, USA) and grown in a plate shaker (Ohaus Orbital Shaker, Ohaus, Parsippany, NJ, USA) at 37 °C, shaking at 900 rpm for 18 h. 10 µL of the grown culture were used to inoculate the main culture in 1 mL LB with the appropriate antibiotic in a 96 deep-well microtiter plate. The pre-cultures in the master plate were mixed with 100 µL sterile 50% glycerol solution, snap frozen in liquid nitrogen and stored at -70 °C. The plate containing the main culture was sealed with Breathe-Easier membrane (Diversified Biotech, Dedham, MA, USA) and incubated in a plate shaker at 37 °C, shaking at 900 rpm for 3 h. After this time, gene expression was induced by adding 10 µL 50 mM IPTG in LB and the cells were grown for 18 h at 37 °C, shaking at 900 rpm. The cells were then harvested by centrifuging for 20 min at 4,000 rcf and the supernatant was discarded. The pellets were snap frozen in liquid nitrogen and stored at -70 °C until further usage.

After thawing the cell pellets, they were lysed by adding 325 µL lysis buffer and mixing at 1200 rpm for 2 min, followed by incubation at 37 °C at 300 rpm for 1 h. Next, 32 µL 11% PEI (linked, average molecular weight 25000 Da, Sigma Aldrich, USA) were added to obtain a final concentration of 1% PEI. The plates were incubated at 37 °C, 1200 rpm for 30 min. Other proteins were then precipitated by transferring the plate to a water bath heated to 80 °C and incubating it for 1 h. Precipitate was removed by centrifugation of the plate for 1 h at 4000 rcf at 4 °C. 250 µL of the supernatant were then transferred to a fresh 96 deep-well microtiter plate, and 500 µL of a saturated aqueous solution of $(NH_4)_2SO_4$ were added before the plate was incubated at 4 °C for 1 h to selectively precipitate PLZ proteins. The plates were then centrifuged again for 1 h at 4,000 rcf at 4 °C, the supernatant was tipped off, and all remaining liquid was removed by tapping the plate on a paper towel upside-down. The protein pellets were resuspended in 150 µL FPLC buffer supplied with 50 µM $CeCl_3$ and shaken at 1000 rpm for 10 min. This protein solution was used for downstream photoreactions. The workflow is also depicted in Figure S11.

3.2.5. Circular dichroism (CD) spectroscopy

CD spectra were recorded on a Chirascan-plus CD spectrometer (*Applied Photophysics*, Leatherhead, England) using a quartz cuvette with 1 mm path length. Spectra between 200 nm and 280 nm were recorded with a bandwidth of 1.0 nm and 1.0 nm steps at 0.5 s per point. The concentration of the

proteins was adjusted to 10 µM by diluting them in H_2O. Three spectra were recorded and averaged before converting the signal from millidegrees to mean residue molar ellipticity.

3.2.6. Protein mass spectrometry

Experiments were performed using electrospray ionization on a SYNAPT XS High Resolution Mass Spectrometer (*Waters*, Milford, MA, USA). 2 µL of a 0.1 mg/mL protein sample were injected and separated on an ACQUITY UPLC Protein BEH C4 column (*Waters*, Milford, MA, USA) using a linear gradient of 5% (*v*/*v*) to 85% (*v*/*v*) acetonitrile in water with a flow rate of 0.4 mL/min at 65 °C. Acetonitrile and water were supplied with 0.1% (*v*/*v*) formic acid to acidify the samples. Results were analyzed using MassLynx v4.2 (*Waters*, Milford, MA, USA).

3.2.7. Photostability studies

To assess potential photodamage after irradiation of the enzyme, we analyzed the protein using SDS-PAGE and full-length protein mass spectrometry (LC-MS). PLZ2.0 and PLZ2.3 were compared and samples were prepared as follows: For the t_0 sample, freshly thawed protein was diluted in reaction buffer (25 mM Hepes, pH 8.5, 100 mM NaCl) and submitted for analysis. For the irradiated samples, the enzymes were diluted to 22.2 µM in reaction buffer and 22.2 µM $CeCl_3$ were added. Next, either 10 µM acetonitrile (for the -substrate sample) or 10 µM 20 mM (*R,R*)-**4** in acetonitrile (for the +substrate sample) were added and the mixture was irradiated for 6 h at 410-420 nm, as described below. After the reaction, the samples were diluted for SDS-PAGE or LC-MS analysis (see 3.2.6.).

3.2.8. Tb(III) binding kinetics

As previously described[3], tryptophan-sensitized Tb(III) luminescence can be used as a signal for specific metal binding. A nearby tryptophan residue is excited by irradiating the protein at 280 nm and subsequently excite the Tb(III) ion *via* energy transfer. This results in a long-lived luminescence signal with a specific spectrum and a maximum emission at 544 nm.

To follow the Tb(III) binding ove time, protein and $TbCl_3$ were diluted to 10 µM in FPLC buffer. The luminescence was measured in a microplate reader (Varioskan LUX, *Thermo Fisher Scientific*) in black 96-well microtiter plates in time-resolved fluorescence mode (TRF; 50 µs delay time, 1 ms integration time, 100 ms measurement time) using 100 µL of the metalloenzyme solution (λ_{ex} = 280 nm, λ_{em} = 544 nm).

3.2.9. Luminescence-based titrations to determine Tb(III) binding affinities

To assess the lanthanide binding affinity of PLZ2.0, titration experiments were performed in triplicates. 1.11 µM PLZ2.0 in FPLC buffer (25 mM HEPES, pH 8.5 or pH 7.0, 100 mM NaCl) were prepared. 90 µL of protein were combined with 10 µL 10-fold concentrated $TbCl_3$ stock in H_2O to reach a final protein concentration of 1 µM and $TbCl_3$ concentrations of 0.1 µM to 8 µM. The mixture was incubated in a black 96-well microtiter plate at room temperature for 1 h to ensure complete equilibration. The terbium luminescence was then measured in a microplate reader (Varioskan LUX, *Thermo Fisher Scientific*) in time-resolved fluorescence mode (TRF; 50 µs delay time, 1 ms integration time, 100 ms measurement time; λ_{ex} = 280 nm, λ_{em} = 544 nm). The signal of the triplicates was averaged and fitted using the following code.

```python
import numpy as np
import pandas as pd
import matplotlib.pyplot as plt
from scipy.optimize import curve_fit
import matplotlib as mpl
import matplotlib.ticker as ticker
import os

# Load the Excel file
file_path = 'Your/Path/Here.xlsx'  # Replace with your file path
sheet_name = 'Tabelle1'
df = pd.read_excel(file_path, sheet_name=sheet_name)

# Extract x values from the first column
data_x = df.iloc[:, 0].tolist()
X_plot = np.linspace(data_x[0], data_x[-1], 300)

# Extract y values from the remaining columns (one column per measurement)
data_y = [df.iloc[:, i].tolist() for i in range(1, df.shape[1])]

# Get the column names as measurement labels
measurement_labels = df.columns[1:].tolist()

# Extract x values
data_x = df['c(TbCl3) [µM]']

# Calculate average and standard deviation across replicates for each concentration level
data_y = df.drop(columns='c(TbCl3) [µM]').mean(axis=1)
data_std = df.drop(columns='c(TbCl3) [µM]').std(axis=1)

# Define the quadratic fit function
def quadratic_func_2var(x, Kd, dymax):
   x=np.array(x)
   return y0+dymax*(((A0+x+Kd)/2)-np.sqrt(((A0+x+Kd)/2)**2-A0*x))/A0

# Initialize A0 as it is provided by the user
A0 = 1

# Calculate initial guess for other parameters
y0 = min(data_y)
dymax = max(data_y) - min(data_y)

#set initial values for variables to be fitted
#p0= [KdAB, Ampl]
p0 = [0.001, dymax]

popt,pcov = curve_fit(quadratic_func_2var, data_x, data_y, p0 = p0)
perr=np.sqrt(np.diag(pcov))
```

```
plt.plot(X_plot, quadratic_func_2var(X_plot, *popt), color = "blue", linestyle="dashed")
plt.errorbar(data_x, data_y, yerr=data_std, fmt = "ks", capsize=3)
plt.ylabel("Tb$^4$ Luminescence [A.U.]")
plt.xlabel("Conc. $TbCl_3$ [µM]")
plt.grid()
text = "$K_d$= "+ str(int(1000*round(popt[0],3)))+ " $\pm$ "+ str(int(1000*round(perr[0],3)))+" nM\
n"
text += "Ampl= " + str(round(popt[1]))+ " $\pm$ "+ str(round(perr[1]))

plt.text(8, 30, text,
         horizontalalignment='left',
         verticalalignment='center',
         fontsize=12)

print("Fitted for 2 variables")
print("KdAB:", str(round(popt[0],3)), "µM; error:", str(round(perr[0],3)), "with p0 = ", str(p0[0]))
print("Ampl:", str(round(popt[1],3)), "; error:", str(round(perr[1],3)), "with p0 = ", str(round(p0[1],3)))

# Set formatting options
plt.rcParams.update({
   'mathtext.default': 'regular',
   'svg.fonttype': 'none',
    'xtick.major.size': 4,      # Major tick length
   'xtick.major.width': 1,   # Major tick width
   'ytick.major.size': 4,
   'ytick.major.width': 1,   # Major tick width
})

# Save plot
plt.tight_layout()
plt.savefig(file_path.replace('.xlsx', '.svg'), format='svg', dpi=300)
plt.savefig(file_path.replace('.xlsx', '.png'), format='png', dpi=300)
plt.show()
```

3.2.10. Isothermal titration calorimetry (ITC) to determine lanthanide binding affinities

ITC was measured on a MicroCal PEAQ-ITC® (Malvern Pananalytical, Worcestershire, UK). Samples were prepared by dialyzing the protein against measurement buffer (25 mM HEPES pH 7.0 or pH 7.5, 100 mM NaCl) over night. The concentration was then adjusted to 20 µM by diluting the samples in dialysis buffer. $CeCl_3$ or $TbCl_3$ solutions were prepared by diluting a 200 mM stock solution of lanthanide in dialysis buffer to a final concentration of 300 µM. The reference cell was filled with MilliQ water. The experimental settings were as follows: Reference power 10 µcal/s, 750 rpm stir speed, 0.4 µL initial injection volume after 60 s delay time, followed by 18x 2 µL injection volume with 150 s spacing time. The background signal was determined by control titrations (metal into buffer) with the same settings. The recorded data were plotted as differential power (DP) against time. The data were analyzed using the manufacturer's analysis software. The background signal was subtracted and the baseline adjusted, and the thermodynamic parameters were determined by fitting the ΔH curve against molar ratio.

3.2.11. UV/Vis absorption studies

For UV/Vis absorption spectroscopy, purified protein was buffer exchanged (2.5 mM HEPES pH 7.5, 10 mM NaCl) using a desalting column (PD MiniTrap™ G-25, *Cytiva*, Marlborough, MA, USA or Zeba™ Spin Desalting Columns 7K MWCO, *Thermo Fisher Scientific*, Waltham, MA, USA). Prepared protein was diluted to 66.6 µM, mixed 1:1 an equally concentrated solution of cerium salt ($CeCl_3$ or $(NH_4)_2[Ce(NO_3)_6]$), and left to incubate at room temperature for 10 minutes. After incubation, the samples were filtered, before 360 µL of metal-bound protein was supplemented with 40 µL of a 1.5 mM stock of substrate **1** in acetonitrile. This led to a final sample concentration of 30 µM protein, 30 µM Ce(III) or Ce(IV) and a fivefold excess of substrate at 150 µM. Control samples were prepared accordingly, leaving out components as indicated. Solutions containing $(NH_4)_2[Ce(NO_3)_6$ were prepared without HEPES, in just 10 mM NaCl.

UV/Vis spectra were recorded in triplicates on a *Jasco* V 750 photometer (*JASCO Deutschland* GmbH, Pfungstadt, Germany) at room temperature (data interval: 0.2 nm; bandwidth: 1 nm; response: 0.06 s, path length: 1 cm). Background absorption was corrected by recording a blank spectrum in advance and subtracting.

3.2.12. DNA and protein sequences of all constructs

Molecular weights (MW) and molar extinction coefficients (ε_{280}) were calculated using the ProtParam tool on the Expasy Server. Genes were expressed from a pET29 plasmid with a C-terminal His_6-tag. Protein is marked in blue, key mutations in green, coordinating Glu / knockout Gln residues in red, and tags in yellow.

PLZ1.4

MW = 38365.7 Da ε_{280} [$M^{-1}cm^{-1}$] = 27500

ATGGGTGACATTCTGATAGTGGCCGCCAAAAACGTTGATGAGATGCTCAAGCAGGTCGAAATTTTGCGTCGCT
TGGGCGCGAAACAGATTGCCGTTGAGAGTAGTGACTGGCGCATACTGCAGGAAGCGCTTAAGAAGGGTGGT
GATATTCTTATTGTATTTGGTGGCGGGATGACCATTACCTTCCGCGGGGATGACCTGGAAGCCTTGCTCAAAGC
AGCTATAGAAATGATAAAGCAGGCACTTAAGTTCGGGGCCACTATAACATTGAGCCTTGACGGAAACGACCTG
AATATTAACATTACCGGCGTCCCAGAGCAGGTCCGCAAGGAGTTGGCTAAACAGGCCGAGCGGCTGGCTAAG
GAATTCGGGATCACAGTGACTCGAACCGGTGGCGGAAACGTGGACGAGATGCTCAAACAGGTGGAGATACTT
AGGAGACTCGGGGCCAAGCAGATCGCCGTGGAGTCTAACGACTGGCGAATACTGCAGGAAGCGTTGAAGAA
AGGTGGCGACATTCTGATTGTCGCGGCTAAGAACGTGGATGAAATGCTCAAGCAGGTCGAAATCCTTAGGCG
TCTCGGCGCCAAACAGATTGCTGTCGAATCCAGTGACTGGAGAATTCTCCAGGAAGCGCTCAAGAAGGGCGG
TGATATCCTTATCGTTGCGGGCGGCGGCATGACAATTACTTTTCGGGGCGACGACCTTGAAGCCCTGCTGAAG
GCCGCCATTGAGATGATTAAACAAGCGCTGAAATTCGGCGCAACCATCACACTCTCCTTGGACGGCAACGATC
TGAACATCAACATCACAGGAGTGCCAGAACAGGTTCGTAAAGAACTGGCGAAACAGGCCGAAAGGCTGGCCA
AGGAATTTGGTATCACAGTTACACGTACGGGTGGAGGCAACGTTGATGAGATGCTGAAACAAGTCGAGATCC
TCCGCCGTCTTGGTGCAAAACAAATCGCAGTGGAGTCCAACGACTGGCGTATTCTTCAAGAGGCGCTTAAGAA
AGGTGGATCCGCTTGGAGCCACCCGCAGTTCGAAAAATAA

MGDILIVAAKNVDEMLKQVEILRRLGAKQIAVESSDWRILQEALKKGGDILIVFGGGMTITFRGDDLEALLKAAIEMI
KQALKFGATITLSLDGNDLNINITGVPEQVRKELAKQAERLAKEFGITVTRTGGGNVDEMLKQVEILRRLGAKQIAVE
SNDWRILQEALKKGGDILIVAAKNVDEMLKQVEILRRLGAKQIAVESSDWRILQEALKKGGDILIVAGGGMTITFRG
DDLEALLKAAIEMIKQALKFGATITLSLDGNDLNINITGVPEQVRKELAKQAERLAKEFGITVTRTGGGNVDEMLKQV
EILRRLGAKQIAVESNDWRILQEALKKGGSAWSHPQFEK*

PLZ1.4_KO

MW = 38361.8 Da ε_{280} [$M^{-1}cm^{-1}$] = 27500

ATGGGTGACATTCTGATAGTGGCCGCCAAAAACGTTGATGAGATGCTCAAGCAGGTCGAAATTTTGCGTCGCT
TGGGCGCGAAACAGATTGCCGTTCAGAGTAGTGACTGGCGCATACTGCAGGAAGCGCTTAAGAAGGGTGGTG
ATATTCTTATTGTATTTGGTGGCGGGATGACCATTACCTTCCGCGGGGATGACCTGGAAGCCTTGCTCAAAGCA
GCTATAGAAATGATAAAGCAGGCACTTAAGTTCGGGGCCACTATAACATTGAGCCTTGACGGAAACGACCTGA
ATATTAACATTACCGGCGTCCCAGAGCAGGTCCGCAAGGAGTTGGCTAAACAGGCCGAGCGGCTGGCTAAGG
AATTCGGGATCACAGTGACTCGAACCGGTGGCGGAAACGTGGACGAGATGCTCAAACAGGTGGAGATACTTA
GGAGACTCGGGGCCAAGCAGATCGCCGTGCAGTCTAACGACTGGCGAATACTGCAGGAAGCGTTGAAGAAA
GGTGGCGACATTCTGATTGTCGCGGCTAAGAACGTGGATGAAATGCTCAAGCAGGTCGAAATCCTTAGGCGTC
TCGGCGCCAAACAGATTGCTGTCCAATCCAGTGACTGGAGAATTCTCCAGGAAGCGCTCAAGAAGGGCGGTG
ATATCCTTATCGTTGCGGGCGGCGGCATGACAATTACTTTTCGGGGCGACGACCTTGAAGCCCTGCTGAAGGC

CGCCATTGAGATGATTAAACAAGCGCTGAAATTCGGCGCAACCATCACACTCTCCTTGGACGGCAACGATCTG
AACATCAACATCACAGGAGTGCCAGAACAGGTTCGTAAAGAACTGGCGAAACAGGCCGAAAGGCTGGCCAAG
GAATTTGGTATCACAGTTACACGTACGGGTGGAGGCAACGTTGATGAGATGCTGAAACAAGTCGAGATCCTCC
GCCGTCTTGGTGCAAAACAAATCGCAGTGCAATCCAACGACTGGCGTATTCTTCAAGAGGCGCTTAAGAAAGG
TGGATCCGCTTGGAGCCACCCGCAGTTCGAAAAATAA

MGDILIVAAKNVDEMLKQVEILRRLGAKQIAVQSSDWRILQEALKKGGDILIVFGGGMTITFRGDDLEALLKAAIEMI
KQALKFGATITLSLDGNDLNINITGVPEQVRKELAKQAERLAKEFGITVTRTGGGNVDEMLKQVEILRRLGAKQIAVQ
SNDWRILQEALKKGGDILIVAAKNVDEMLKQVEILRRLGAKQIAVQSSDWRILQEALKKGGDILIVAGGGMTITFRG
DDLEALLKAAIEMIKQALKFGATITLSLDGNDLNINITGVPEQVRKELAKQAERLAKEFGITVTRTGGGNVDEMLKQV
EILRRLGAKQIAVQSNDWRILQEALKKGGSAWSHPQFEK*

PLZ2.0

MW = 36740.5 Da ε_{280} $[M^{-1}cm^{-1}]$ = 20970

ATGGCCGACATATTGATCGTGCAAGATCTTGACCCGGATGCTAAGCTGGCTCAAGTCCGAGAACTGCGTGCGG
CTGGTGCTCGCATTATTGCGGTTGAAGACGCTGATGCCGACCTGCTGATTCGAGCAATGGATCTTGGCGCAGA
TATTCTTGTAGTACTCGGCCGGAAAGTCTCCATCAAATCCGACACGGTGGAACAGTTGTTAGCTACTGTCCGGT
TCGCCATGGAAAGAGCTCACGAGCTTGGTCTGGATATCTGGATCGTTGTAAAAGATAACAATATTTACATTTTC
TTTGCATCTGCTCCGGAGCAAGTTGCCCAATTTGTCGCGGCTCTGACGGCATTCTCGAAGGAGCAGGGATTAG
AAATCAAAGTAATTGACCAGGATCCCCTGGAAAATATCCGCCGCCTGCGAGAATATGGCGCAAAAATTATTGC
CTATGAAGACGATAACGCTGACCGTCTTATTCGCGCTCTGGAGGCAGGCGCCGACATTCTGATTGTTCAGGCA
GCCGACATTGAGGCCACCGTTGAAGCCATTAGACGTTTACGAGAAGCAGGGGCTAAGATCATAGCCGTTGAA
AGCGCTAACTTAGAACAACTGAAAGCTGCACTCGAACTGGGAGCGGATATACTGATCATACAGGGTCGCGAA
GTTGTCGTTCGTAGTGATACCTTTCAAGAAGCAATCGAAGTTGCCCTGTTCGTGGTTAAGAAAGCTTGGGAAG
CGGGGGTTACCGTTGCGCTGCGTTTACGAGAAAACACGCTGCGCGTTATATTTGCAATGACCCCGGAACAACT
GGCCGAATTAATCGCACAACTTCGCGCCCTGGCAGCTGAAAAGGGCTGGGAAATTCGGGTTTTTGACACGGAT
CCGCTTGCGGCAATGCGTGAATTACGCGAACTGGGGGCAAAAATTATTGCGTTAGAATCCCCGGATTTGGATG
TATTACTTGCTGGTTTGCGAGCAGCAGGCGGTTCACATCATCATCACCACCATTGA

MADILIVQDLDPDAKLAQVRELRAAGARIIAVEDADADLLIRAMDLGADILVVLGRKVSIKSDTVEQLLATVRFAME
RAHELGLDIWIVVKDNNIYIFFASAPEQVAQFVAALTAFSKEQGLEIKVIDQDPLENIRRLREYGAKIIAYEDDNADRLI
RALEAGADILIVQAADIEATVEAIRRLREAGAKIIAVESANLEQLKAALELGADILIIQGREVVVRSDTFQEAIEVALFVV
KKAWEAGVTVALRLRENTLRVIFAMTPEQLAELIAQLRALAAEKGWEIRVFDTDPLAAMRELRELGAKIIALESPDLD
VLLAGLRAAGGSHHHHHH*

PLZ2.0_KO

MW = 36736.6 Da ε_{280} $[M^{-1}cm^{-1}]$ = 20970

ATGGCCGACATATTGATCGTGCAAGATCTTGACCCGGATGCTAAGCTGGCTCAAGTCCGAGAACTGCGTGCGG
CTGGTGCTCGCATTATTGCGGTTCAGGACGCTGATGCCGACCTGCTGATTCGAGCAATGGATCTTGGCGCAGA
TATTCTTGTAGTACTCGGCCGGAAAGTCTCCATCAAATCCGACACGGTGGAACAGTTGTTAGCTACTGTCCGGT
TCGCCATGGAAAGAGCTCACGAGCTTGGTCTGGATATCTGGATCGTTGTAAAAGATAACAATATTTACATTTTC
TTTGCATCTGCTCCGGAGCAAGTTGCCCAATTTGTCGCGGCTCTGACGGCATTCTCGAAGGAGCAGGGATTAG

AAATCAAAGTAATTGACCAGGATCCCCTGGAAAATATCCGCCGCCTGCGAGAATATGGCGCAAAAATTATTGC
CTATCAGGACGATAACGCTGACCGTCTTATTCGCGCTCTGGAGGCAGGCGCCGACATTCTGATTGTTCAGGCA
GCCGACATTGAGGCCACCGTTGAAGCCATTAGACGTTTACGAGAAGCAGGGGCTAAGATCATAGCCGTTCAG
AGCGCTAACTTAGAACAACTGAAAGCTGCACTCGAACTGGGAGCGGATATACTGATCATACAGGGTCGCGAA
GTTGTCGTTCGTAGTGATACCTTTCAAGAAGCAATCGAAGTTGCCCTGTTCGTGGTTAAGAAAGCTTGGGAAG
CGGGGGTTACCGTTGCGCTGCGTTTACGAGAAAACACGCTGCGCGTTATATTTGCAATGACCCCGGAACAACT
GGCCGAATTAATCGCACAACTTCGCGCCCTGGCAGCTGAAAAGGGCTGGGAAATTCGGGTTTTTGACACGGAT
CCGCTTGCGGCAATGCGTGAATTACGCGAACTGGGGGCAAAAATTATTGCGTTACAGTCCCCGGATTTGGATG
TATTACTTGCTGGTTTGCGAGCAGCAGGCGGTTCACATCATCATCACCACCATTGA

MADILIVQDLDPDAKLAQVRELRAAGARIIAVQDADADLLIRAMDLGADILVVLGRKVSIKSDTVEQLLATVRFAME
RAHELGLDIWIVVKDNNIYIFFASAPEQVAQFVAALTAFSKEQGLEIKVIDQDPLENIRRLREYGAKIIAYQDDNADRLI
RALEAGADILIVQAADIEATVEAIRRLREAGAKIIAVQSANLEQLKAALELGADILIIQGREVVVRSDTFQEAIEVALFVV
KKAWEAGVTVALRLRENTLRVIFAMTPEQLAELIAQLRALAAEKGWEIRVFDTDPLAAMRELRELGAKIIALQSPDL
DVLLAGLRAAGGSHHHHHH*

PLZ2.1

MW = 36668.4 Da ε_{280} [$M^{-1}cm^{-1}$] = 15470

ATGGCCGACATATTGATCGTGCAAGATCTTGACCCGGATGCTAAGCTGGCTCAAGTCCGAGAACTGCGTGCGG
CTGGTGCTCGCATTATTGCGGTTGAAGACGCTGATGCCGACCTGCTGATTCGAGCAATGGATCTTGGCGCAGA
TATTCTTGTAGTACTCGGCCGGAAAGTCTCCATCAAATCCGACACGGTGGAACAGTTGTTAGCTACTGTCCGGT
TCGCCATGGAAAGAGCTCACGAGCTTGGTCTGGATATCAATATCGTTGTAAAAGATAACAATATTTACATTTTC
TTTGCATCTGCTCCGGAGCAAGTTGCCCAATTTGTCGCGGCTCTGACGGCATTCTCGAAGGAGCAGGGATTAG
AAATCAAAGTAATTGACCAGGATCCCCTGGAAAATATCCGCCGCCTGCGAGAATATGGCGCAAAAATTATTGC
CTATGAAGACGATAACGCTGACCGTCTTATTCGCGCTCTGGAGGCAGGCGCCGACATTCTGATTGTTCAGGCA
GCCGACATTGAGGCCACCGTTGAAGCCATTAGACGTTTACGAGAAGCAGGGGCTAAGATCATAGCCGTTGAA
AGCGCTAACTTAGAACAACTGAAAGCTGCACTCGAACTGGGAGCGGATATACTGATCATACAGGGTCGCGAA
GTTGTCGTTCGTAGTGATACCTTTCAAGAAGCAATCGAAGTTGCCCTGTTCGTGGTTAAGAAAGCTTGGGAAG
CGGGGGTTACCGTTGCGCTGCGTTTACGAGAAAACACGCTGCGCGTTATATTTGCAATGACCCCGGAACAACT
GGCCGAATTAATCGCACAACTTCGCGCCCTGGCAGCTGAAAAGGGCTGGGAAATTCGGGTTTTTGACACGGAT
CCGCTTGCGGCAATGCGTGAATTACGCGAACTGGGGGCAAAAATTATTGCGTTAGAATCCCCGGATTTGGATG
TATTACTTGCTGGTTTGCGAGCAGCAGGCGGTTCACATCATCATCACCACCATTGA

MADILIVQDLDPDAKLAQVRELRAAGARIIAVEDADADLLIRAMDLGADILVVLGRKVSIKSDTVEQLLATVRFAME
RAHELGLDINIVVKDNNIYIFFASAPEQVAQFVAALTAFSKEQGLEIKVIDQDPLENIRRLREYGAKIIAYEDDNADRLI
RALEAGADILIVQAADIEATVEAIRRLREAGAKIIAVESANLEQLKAALELGADILIIQGREVVVRSDTFQEAIEVALFVV
KKAWEAGVTVALRLRENTLRVIFAMTPEQLAELIAQLRALAAEKGWEIRVFDTDPLAAMRELRELGAKIIALESPDLD
VLLAGLRAAGGSHHHHHH*

PLZ2.2

MW = 36698.4 Da ε_{280} [$M^{-1}cm^{-1}$] = 20970

ATGGCCGACATATTGATCGTGCAAGATCTTGACCCGGATGCTAAGCTGGCTCAAGTCCGAGAACTGCGTGCGG
CTGGTGCTCGCATTATTGCGGTTGAAGACGCTGATGCCGACCTGCTGATTCGAGCAATGGATCTTGGCGCAGA
TATTCTTGTAGTACTCGGCCGGAAAGTCTCCATCAAATCCGACACGGTGGAACAGTTGTTAGCTACTGTCCGGT
TCGCCATGGAAAGAGCTCACGAGCTTGGTCTGGATATCTGGATCGGTGTAAAAGATAACAATATTTACATTTTC
TTTGCATCTGCTCCGGAGCAAGTTGCCCAATTTGTCGCGGCTCTGACGGCATTCTCGAAGGAGCAGGGATTAG
AAATCAAAGTAATTGACCAGGATCCCCTGGAAAATATCCGCCGCCTGCGAGAATATGGCGCAAAAATTATTGC
CTATGAAGACGATAACGCTGACCGTCTTATTCGCGCTCTGGAGGCAGGCGCCGACATTCTGATTGTTCAGGCA
GCCGACATTGAGGCCACCGTTGAAGCCATTAGACGTTTACGAGAAGCAGGGGCTAAGATCATAGCCGTTGAA
AGCGCTAACTTAGAACAACTGAAAGCTGCACTCGAACTGGGAGCGGATATACTGATCATACAGGGTCGCGAA
GTTGTCGTTCGTAGTGATACCTTTCAAGAAGCAATCGAAGTTGCCCTGTTCGTGGTTAAGAAAGCTTGGGAAG
CGGGGGTTACCGTTGCGCTGCGTTTACGAGAAAACACGCTGCGCGTTATATTTGCAATGACCCCGGAACAACT
GGCCGAATTAATCGCACAACTTCGCGCCCTGGCAGCTGAAAAGGGCTGGGAAATTCGGGTTTTTGACACGGAT
CCGCTTGCGGCAATGCGTGAATTACGCGAACTGGGGGCAAAAATTATTGCGTTAGAATCCCCGGATTTGGATG
TATTACTTGCTGGTTTGCGAGCAGCAGGCGGTTCACATCATCATCACCACCATTGA

MADILIVQDLDPDAKLAQVRELRAAGARIIAVEDADADLLIRAMDLGADILVVLGRKVSIKSDTVEQLLATVRFAME
RAHELGLDIWIGVKDNNIYIFFASAPEQVAQFVAALTAFSKEQGLEIKVIDQDPLENIRRLREYGAKIIAYEDDNADRLI
RALEAGADILIVQAADIEATVEAIRRLREAGAKIIAVESANLEQLKAALELGADILIIQGREVVVRSDTFQEAIEVALFVV
KKAWEAGVTVALRLRENTLRVIFAMTPEQLAELIAQLRALAAEKGWEIRVFDTDPLAAMRELRELGAKIIALESPDLD
VLLAGLRAAGGSHHHHHH*

PLZ2.3

MW = 36626.3 Da ε_{280} [$M^{-1}cm^{-1}$] = 15470

ATGGCCGACATATTGATCGTGCAAGATCTTGACCCGGATGCTAAGCTGGCTCAAGTCCGAGAACTGCGTGCGG
CTGGTGCTCGCATTATTGCGGTTGAAGACGCTGATGCCGACCTGCTGATTCGAGCAATGGATCTTGGCGCAGA
TATTCTTGTAGTACTCGGCCGGAAAGTCTCCATCAAATCCGACACGGTGGAACAGTTGTTAGCTACTGTCCGGT
TCGCCATGGAAAGAGCTCACGAGCTTGGTCTGGATATCAACATCGGCGTAAAAGATAACAATATTTACATTTTC
TTTGCGTCTGCTCCGGAGCAAGTTGCCCAATTTGTCGCGGCTCTGACGGCATTCTCGAAGGAGCAGGGATTAG
AAATCAAAGTAATTGACCAGGATCCCCTGGAAAATATCCGCCGCCTGCGAGAATATGGCGCAAAAATTATTGC
CTATGAAGACGATAACGCTGACCGTCTTATTCGCGCTCTGGAGGCAGGCGCCGACATTCTGATTGTTCAGGCA
GCCGACATTGAGGCCACCGTTGAAGCCATTAGACGTTTACGAGAAGCAGGGGCTAAGATCATAGCCGTTGAA
AGCGCTAACTTAGAACAACTGAAAGCTGCACTCGAACTGGGAGCGGATATACTGATCATACAGGGTCGCGAA
GTTGTCGTTCGTAGTGATACCTTTCAAGAAGCAATCGAAGTTGCCCTGTTCGTGGTTAAGAAAGCTTGGGAAG
CGGGGGTTACCGTTGCGCTGCGTTTACGAGAAAACACGCTGCGCGTTATATTTGCAATGACCCCGGAACAACT
GGCCGAATTAATCGCACAACTTCGCGCCCTGGCAGCTGAAAAGGGCTGGGAAATTCGGGTTTTTGACACGGAT
CCGCTTGCGGCAATGCGTGAATTACGCGAACTGGGGGCAAAAATTATTGCGTTAGAATCCCCGGATTTGGATG
TATTACTTGCTGGTTTGCGAGCAGCAGGCGGTTCACATCATCATCACCACCATTGA

MADILIVQDLDPDAKLAQVRELRAAGARIIAVEDADADLLIRAMDLGADILVVLGRKVSIKSDTVEQLLATVRFAME
RAHELGLDINIGVKDNNIYIFFASAPEQVAQFVAALTAFSKEQGLEIKVIDQDPLENIRRLREYGAKIIAYEDDNADRLI
RALEAGADILIVQAADIEATVEAIRRLREAGAKIIAVESANLEQLKAALELGADILIIQGREVVVRSDTFQEAIEVALFVV
KKAWEAGVTVALRLRENTLRVIFAMTPEQLAELIAQLRALAAEKGWEIRVFDTDPLAAMRELRELGAKIIALESPDLD
VLLAGLRAAGGSHHHHHH*

4. Computational methods

4.1. Design of PLZ2.0 using RFdiffusion and LigandMPNN

To reduce its cavity size, the photoenzyme was redesigned computationally. Starting from the Alphafold prediction[3] of PLZ1.4, lid domain was lowered 3 Å towards the TIM barrel using PyMOL's translate function.[5] To accommodate for this new configuration and enable sensible connectivity, the original inter-domain linker regions (residues 53-56, 129-135, 222-225 and 298-304) were removed. Subsequently, new linkers of 2 or 3 residues were generated using RFdiffusion.[6] In total, 22 residues were removed and then replaced by two linkers with 2 residues and two linkers with 3 residues. This resulted in a shortening of the construct by 12 residues. Since this diffusion step is trivial, the difference between results was marginal and the first result was chosen arbitrarily for further optimization. The lowering of the lid domain created a new interface between the segments of the protein. To allow for interface optimization, the entire top domain was partially diffused using 15 steps of diffusion (out of a total of 50).[6] After manual consideration, one backbone was chosen and the original docking results of substrate (*R,R*)-**4** in PLZ1.4 were superimposed with the new scaffold to get an estimation for the ligand position. Utilizing this ligand position, LigandMPNN was used to populate the amino acid sequence using a softmax temperature of 0.1, while fixing the metal-coordinating amino acids (E31, E148, E194 and E311 in PLZ1.4, corresponding to E31, E147, E193, E309 in PLZ2.0).[7] In total, 100 sequences were obtained which were predicted first using ESMfold with 3 recycles each.[8] Of these predictions, all structures exhibiting an average pLDDT of greater than 90 were resubmitted for structure prediction using Colabfold (3 models with 3 recycles each per sequence).[9] Finally, all structures with an average pLDDT of lower than 90 were once again discarded, leaving 13 final sequences for manual consideration.

Based on cavity size, shape and domain specific prediction confidence, 4 structures were chosen for further evaluation. The metal ion position was approximated by superimposition with the structurally similar PLZ1.4 and a short MD simulation was performed with the metal ion to relax the structure.[10-12] After this, diol **4** was docked into the cavity using VINA.[13, 14] The results were once again analyzed manually and a preferred structure was chosen.

To remove surface metal binding sites of this structure, it was analyzed using BioMetAll.[15] The AlphaFold2 predictions of the designed protein scaffolds were used as an input and the program was run using the standard parameters. Identified clusters of metal-coordinating residues were removed by introducing cysteine to serine, glutamate to glutamine and aspartate to asparagine mutations (E6Q, C116S, E128Q, D151N, E169Q, C223S, E227Q and E274Q in PLZ1.4, corresponding to E6Q, C115S, E127Q, D150N, E168Q, C222S, E226Q and E272Q in PLZ2.3). Finally, two loops in the ferredoxin domain exhibited lower model prediction confidence, which warranted the deletion of a single residue (A100 and A261) in each loop to ensure model confidence. This resulted in a sequence that was overall 14 amino acids shorter than PLZ1.4, as also visualized in the sequence alignment in Fig. S1. For purification purposes, a C-terminal His_6-Tag was attached after a short unspecific GGS linker to prevent interaction between the tag and the protein. The acquired sequence for PLZ2.0 was ordered as a clonal gene in a pET29b vector from Twist Bioscience.

4.2. Docking and molecular dynamics (MD) simulations

Structure modeling and preparation: A missing residue (E283) in the X-ray structure of PLZ2.3 was modelled using MODELLER.[16] The structures of PLZ2.0, PLZ2.1, and PLZ2.2 were derived from the crystal structure of PLZ2.3. Residues N85W and G87V in the X-ray structure of PLZ2.3 were mutated to the respective residues using Maestro molecular modeling program in Schrödinger (release version 2024-1, accessed *via* the Leibniz Supercomputing Center, LRZ).[17] Full length PLZ1.4 in complex with the Ce(III) metal ion and substrate **4** (*RR* or *SS*) was modeled using Boltz2.[18] All structures (PLZ1.4, 2.0, 2.1, 2.2, and 2.3) were further prepared using the protein-preparation tool in Maestro to add hydrogen atoms and perform restrained energy minimization before docking runs were conducted. The Boltz2 predicted ligand conformation of PLZ1.4 was used as a reference for all structures to generate a grid center to perform ligand docking. Utilizing the Glide docking protocol,[19] docking of both enantiomers ((*R*,*R*)-**4** and (*S*,*S*)-**4**) was conducted separately on all protein-systems. Default parameters were used in the Glide docking program and 20 poses per ligand were obtained. Finally, the docked conformation with the lowest energy was chosen for further optimization using 100 ns MD simulations. The conformations shown in Fig. 3 depict the substrate conformations adopted in the last frame of the MD simulations.

MD simulation procedure: The molecular dynamics simulations were performed using the AMBER software package.[11, 20] The latest AMBER force field for proteins ff19SB,[21] the OPC water model, and LJ1264 metal ion parameters for Ce(III) were used.[22] For system preparation, charges on proteins were neutralized by adding Na^+ ions, and the OPC water model was used to solvate the protein in a cubic box with a padding of 20 Å.

Following the preparation, gradual energy minimization was performed in 14 steps, applying restraints on the protein's heavy atoms, while no restraints were used on water, ions, and hydrogen atoms. The restraints were gradually reduced, starting from 1,000 kcal*mol^{-1}* $Å^{-2}$ to no restraints in the last step. The steepest descent followed by conjugate gradient algorithms was used for energy minimization in each step.

Heating simulations using NVT ensembles – constant number of particles (N), volume (V), and temperature (T) – were performed by a gradual increase of temperature from 10 K to 300 K in the first 30 ps. The Langevin thermostat algorithm was used to control bath temperature, keeping the collision frequency at 0.5 ps^{-1}. The integration time was set to 1.0 fs, and the SHAKE algorithm[22] was used to constrain bonds involving hydrogen atoms. Periodic boundary conditions were applied. Heating simulations were performed using weak constraints on heavy atoms (50 kcal*mol^{-1}*$Å^{-2}$), which were gradually decreased.

After heating, the equilibration simulation with constant pressure was performed, utilizing an NPT ensemble (constant number of particles (N), pressure (P), and temperature (T)). Equilibration simulations of 5 ns with constraints of 5.0 kcal*mol^{-1}*$Å^{-2}$ were performed before running a 20 ns simulation with no constraints. The pressure was maintained using the Berendsen barostat. The integration time was increased to 2.0 fs in the second NPT simulation, and trajectory frames and restart files were saved every 100 ps.

MD simulation analysis: The MD trajectories were analyzed for the overall protein and ligand dynamics by calculating root mean square deviation (RMSD) of protein and ligand to the starting position. MD trajectories were also used to perform binding pocket volume calculation using Mdpocket with default parameters.[24] The trajectories and structure files were further visualized using VMD86[25] and Pymol[5] (Schrodinger 2015).

4.3. Blind docking of larger substrates

Glide[19] was used to perform blind docking of various diol substrates with increasing size into the crystal structure of PLZ2.3. The whole protein was chosen for blind docking by increasing the internal grid XYZ dimensions to maximum (40 Å). The initial docking number was set to 5000, and 5000 poses were chosen to undergo final refinement. The pose displacement and penalty were set off to report all binding poses and to avoid default filtering criteria (penalties and distance displacement). Final docked poses to report were set to 500 poses per ligand.

Analysis: The blindly docked ligands were filtered for productive binding poses, where at least one oxygen atom of the substrate hydroxy groups is within 3 Å to the Ce(III) ion. The number of these binding poses out of total docked conformations was then plotted for all ligands.

4.4. 2D interaction representation

Selected frames of the MD simulations were chosen for 2D interaction analysis using the academic version of the Flare software[26] (Cresset, Litlington, Cambridgeshire, UK) using default parameters.

5. Enzymatic assays

5.1. Procedures for photoenzymatic reactions

5.1.1. 6-vial photoreactor setup

A custom 6-well photoreactor was designed to ensure uniform irradiation of all samples in GC vials. LEDs were purchased at *Avonec* (*Avonec*, Wesel, Germany) as high power LEDs soldered on starboards (410–420 nm 3 W). The LEDs were glued using Keratherm Bond 100 RT thermal adhesive (*KERAFOL*, Eschenbach in der Oberpfalz, Germany) onto *Fischer Elektronik* SK 42 heat sinks (100x160x25 mm, aluminum, 0.95K/W; *Fischer Elektronik*, Lüdenscheid, Germany). The heat sink was cooled by a water chilled hollow aluminum block with the temperature of the cooling water set to 18 °C, resulting in a reaction temperature of 23 °C. The MW LCM-40 LED drivers (*MEAN WELL Enterprises* Co, New Taipei City, Taiwan) were operated at a constant current of 0.7 A. The setup is depicted in Figure S26.

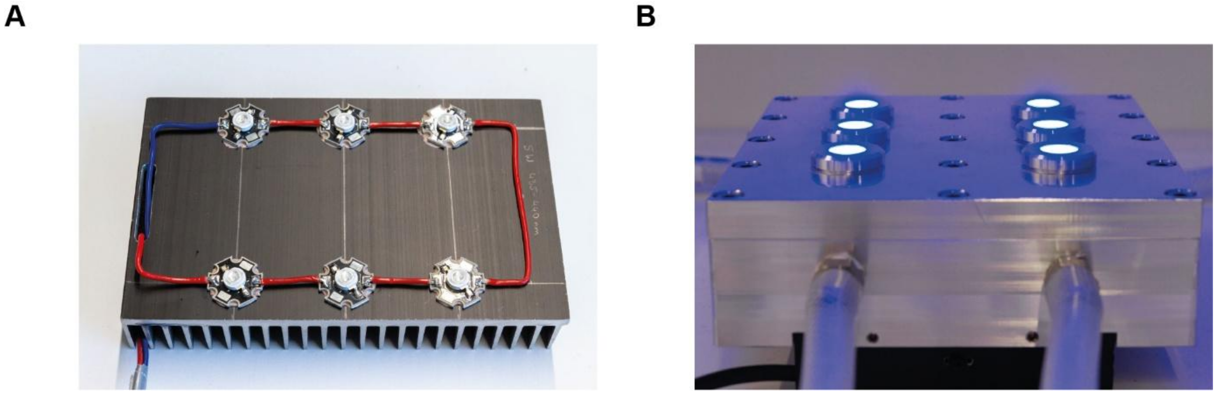

Figure S26: 6- vial photoreactor. (A) LEDs on starboards glued to the heat sink. (B) The cooled aluminum block used to position each GC vial directly above one LED.

5.1.2. Vial-based photoreaction procedure

Photoreactions were run as described previously.[3] In brief, a stock solution of 22.2 µM enzyme and 22.2 µM $CeCl_3$ (for initial experiments: 111 µM of each to reach a final catalyst loading of 5 mol%) were prepared in 25 mM HEPES, 100 mM NaCl, pH 8.5. 90 µL of this solution were transferred to a 1.5 mL GC vial and 10 µL substrate solution (20 mM in acetonitrile) were added to reach a final concentration of 20 µM metalloenzyme and 2 mM substrate with 10% acetonitrile as co-solvent (1 mol% catalyst loading). The vials were then irradiated in the 6-well photoreactor at 410-420 nm for 6 h (unless stated otherwise. For initial experiments with 5 mol% catalyst loading: 24 h).

5.1.3. Procedure for plate-based photoreactions for directed evolution

For directed evolution, the proteins were purified and loaded with $CeCl_3$ as described above. 90 µL of the protein were combined with 10 µL 20 mM diol **4** in acetonitrile in a U-bottom 96-well microtiter plate. The plates were sealed with clear silicone adhesive film (Cat No. 89134-428, VWR International, Radnor, PA, USA) and subsequently irradiated at 405 nm for 24 h on the middle shelf of a UV LED curing system (Novachem, Ireland, see Figure S27) with alternating 10 s on-time and 10 s off-time (total 12 h on-time) of the lamps. The plates were cooled to 15 °C with a water cooler attached to a custom cooling device (see Figure S27). After irradiation, the plates were shaken at 1200 rpm for 5 min to homogenize and 33 µL were taken off and stored in a separate, sealed plate for potential re-analysis

later. To the rest, 132 µL methanol (without internal standard due to a similar retention time to the substrates on the HPLC) were added to precipitate the protein. The plate was sealed again with clear adhesive silicone film and shaken for 5 min at 1200 rpm before centrifuging it at 4000 rcf for 10 min. 120 µL supernatant were transferred to a polypropylene V-bottom 96-well microtiter plate, sealed with SureSTART re-sealing tape for autosamplers (Cat No. 60180-M146, Thermo Fisher Scientific Inc., Waltham, MA, USA), and measured with HPLC method 4 (chiral column). All peaks were then manually integrated and hits were identified by comparing the area of the product peak and the ratio of the remaining substrate enantiomer peaks to the positive controls of the parent variant that were run on the same plate in quadruplicates.

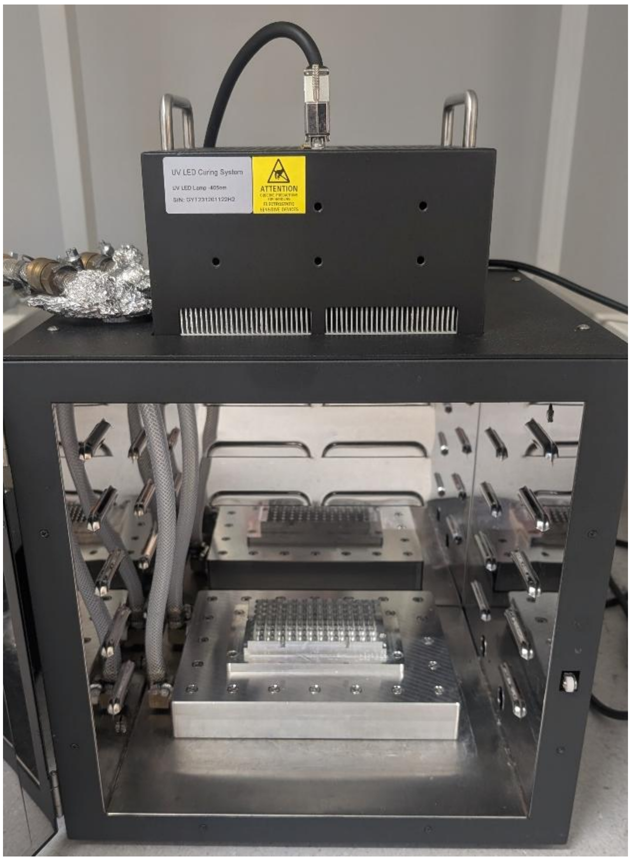

Figure S27: LED curing chamber and the custom made, water-cooled plate holder used for the irradiation of 96-well microtiter plates.

5.1.4. Determination of yields by quantification of HPLC peaks

Yields of diol cleavage reactions were determined by integrating HPLC peaks, using Method 1 (achiral column). After irradiation, the reactions were worked up by addition of 200 µL acetonitrile containing 100 µM 1,3,5-Trimethoxybenzene (TMB) as an internal standard. The precipitated protein was removed by centrifugation. 150 µL supernatant was taken off and further diluted with 100 µL water and 50 µL 200 µM TMB to have a final dilution of 1:6 and a TMB concentration of 100 µM TMB. This mixture was then analyzed using HPLC method 1 (non-chiral). Yields were quantified by the peak area ratio of benzaldehyde **2** at 254 nm and the internal standard at 205 nm using the calibration curves shown below.

5.1.5. Determination of enantioselectivity from chiral HPLC measurements

For evaluating the stereoselectivity of the photoenzymes towards the photocatalytic cleavage of **4**, triplicates of the photoreactions with the respective enzyme were set up according to the general methods and irradiated for 6 h unless specified otherwise. After irradiation, the reactions were worked up by adding 200 µL 200 µM benzophenone in acetonitrile as an internal standard. The precipitated protein was removed by centrifugation and 150 µL supernatant was further diluted by adding 150 µL water and 100 µL 200 µM benzophenone in acetonitrile. This resulted in an overall eight-fold dilution and a final concentration of internal standard of 100 µM. The analytes were separated on a chiral Chiralcel OJ-RH column at room temperature according to HPLC Method 2 (chiral column). The figures shown in the main text show the areas of the chromatogram that are relevant to quantify the unreacted substrate stereoisomers. The full chromatograms are shown below.

The assignment of the peaks to the corresponding stereoisomers was supported by stereoselective synthesis of (*R*,*R*)-**4** and (*S*,*S*)-**4** by catalytic asymmetric dihydroxylation[27] as reported in ref.[3]

When peaks were not base line separated, they were split symmetrically using the "split peaks" function of the Chromeleon software. The areas of the peaks were determined using the Chromeleon software and the average and standard deviation of the triplicates were calculated.

The enantiomeric excess *ee* was calculated as follows:

$$ee = \frac{\left|\left(\frac{A^E_{S,S}}{A^C_{S,S}}\right) - \left(\frac{A^E_{R,R}}{A^C_{R,R}}\right)\right|}{\left(\frac{A^E_{S,S}}{A^C_{S,S}}\right) + \left(\frac{A^E_{R,R}}{A^C_{R,R}}\right)}$$

where $A^E_{S,S}$ and $A^C_{S,S}$ are the areas of the peaks on the HPLC of the (*S,S*)-enantiomer for the reaction with enzyme and the control reaction (just buffer with $CeCl_3$), respectively. $A^E_{R,R}$ and $A^C_{R,R}$ are the areas of the HPLC peaks of the (*R,R*)-enantiomer for the reaction with enzyme and the control reaction, respectively. The final areas are divided by the initial areas to compensate for mistakes in the integration of the peaks, as some of the peaks are not baseline separated. The influence of these inaccuracies on the *ee* is minimized by this additional step.

The enantioselectivity value S is a better measure for the performance of a catalyst designed for kinetic resolution. S was determined according to:

$$S = \frac{ln[(1-c)(1-ee)]}{ln[(1-c)(1+ee)]}$$

where c is the conversion of the reaction. c was calculated by subtracting the areas of each enantiomer after the reaction from the respective areas in the control with no enzyme (buffer + $CeCl_3$). The sum of these corrected areas of remaining substrates was then divided by the sum of the substrate areas in the control.

5.1.6. Time course of the cleavage of (*R,R*)-4 and (*S,S*)-4

To determine the performance of PLZ2.3 in a kinetic resolution setting, a time course of the consumption of (*R,R*)-**4** and (*S,S*)-**4** was measured. 111 µM PLZ2.3 were prepared in FPLC buffer (25 mM HEPES, pH 8.5, 100 mM NaCl) supplied with 111 µM $CeCl_3$. 90 µL of this mixture were combined with 10 µL 20 mM (*R,R*)- **4** and (*S,S*)-**4** (without *meso*-**4**, see Fig. S13 for the separation) to

achieve a final concentration of 100 µM PLZ2.3 and 2 mM substrate in 100 µL. Five of these samples were then irradiated according to the standard protocol in a 6-vial photoreactor and one reaction was stopped after 30 min, 60 min, 90 min, 2 h, and 4 h, respectively. The samples were then worked up by adding 200 µL 200 µM benzophenone in acetonitrile as an internal standard. The precipitated protein was removed by centrifugation and 150 µL supernatant was further diluted by adding 150 µL water and 100 µL 200 µM benzophenone in acetonitrile. This resulted in an overall eight-fold dilution and a final concentration of internal standard of 100 µM. The mixture was analyzed using HPLC method 2 (chiral column). The peaks corresponding to the substrate enantiomers were then manually integrated and their integrals were used to determine the enantiomeric excess. A control that was not irradiated was used as the t_0 sample. The chromatograms are shown in Figure S28. As seen in the full chromatogram, the reaction produced several side products additionally to main product benzaldehyde **2**. As postulated previously,[3] the cleavage of substrate **4** produces mainly benzaldehyde **2** due to the higher stability of the benzyl radical intermediate. The side products presumably form due to alternative oxidation reaction routes of the produced radical intermediates after β-scission of the diol substrate.

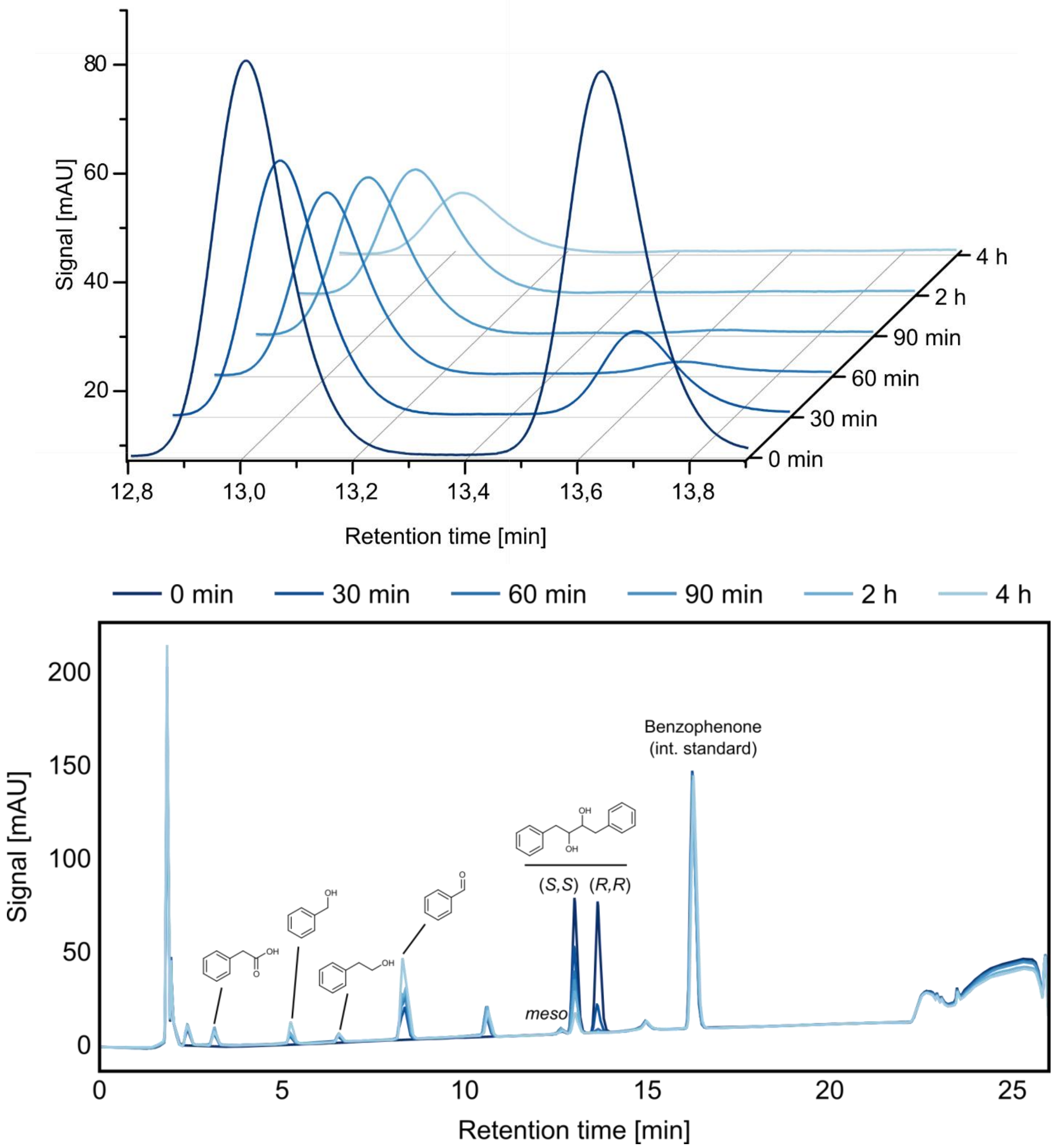

Reaction time	Area int. std., 205 nm [mAU*min]	Area **2**, 254 nm [mAU*min]	Area (*S,S*)-**4**, 205 nm [mAU*min]	Area (*R,R*)-**4**, 205 nm [mAU*min]
0 min	22,632	0,024	11,618	11,629
30 min	22,978	1,264	7,515	2,516
60 min	22,739	1,971	5,435	0,407
90 min	22,726	2,154	4,777	0,119
2 h	22,513	2,606	3,706	-
4 h	23,083	4,613	1,794	-

Figure S28: Chromatograms of the time course experiment for kinetic resolution. Top: zoom to the relevant portion. Middle: Full chromatograms with assigned molecules

5.1.7. Michaelis-Menten kinetics for (*R*,*R*)-1

5.5 µM enzyme were incubated with one equivalent $CeCl_3$ for 2.5 h at 40 °C. After addition of 10 µL substrate solution (0–120 mM in acetonitrile) to 90 µL of metalloenzyme, the photoreactions were carried out in 6-vial photoreactors at 410-420 nm (3 W) at approx. 23 °C for 1 h with a final concentration of 5 µM metalloenzyme, 0–12 mM substrate and 10% (*v*/*v*) acetonitrile as co-solvent. The initial velocities of benzaldehyde (**2**) formation were quantified in triplicates, whereby a formation of two benzaldehyde (**2**) molecules was assumed for the conversion of one substrate molecule (*R*,*R*)-**1**. The kinetic parameters k_{cat} and K_M were determined by fitting the data with the Michaelis Menten equation, with $[E]_0$ = enzyme concentration and $[S]_0$ = substrate concentration:

$$k_{obs} = \frac{v}{[E]_0} = \frac{k_{cat}\ [S]_0}{K_M + [S]_0}$$

k_{obs} was determined using the following formula:

$$k_{obs}[h^{-1}] = \frac{\frac{\frac{c(product)[\mu M]}{2\ molecules\ product\ per\ substrate}}{irradiation\ time\ [h]}}{c(enzyme)[\mu M]} * dilution\ factor\ for\ HPLC$$

5.1.8. Michaelis-Menten kinetics for the individual enantiomers of diol 4

Due to the range of side products generated upon cleavage of substrate **4**, the kinetics of its cleavage were determined from the substrate consumption. For this reason, the reactions were prepared with a volume of 200 µL (twice as large as required) and split before the irradiation of the reaction. The non-irradiated sample was worked up and analyzed identically to the irradiated samples to determine $[S]_0$ independently of small pipetting inaccuracies.

11.1 µM photoenzyme was prepared in 25 mM HEPES, 100 mM NaCl, pH 8.5, and $CeCl_3$ was added to a final concentration of 11.1 µM. 180 µL of this mixture were combined with 20 µL of a 10x stock of substrate **4** in acetonitrile and the reaction was split in two parts as described above. One of these parts was then irradiated at 410-420 nm in the 6-well photoreactor for 20 min. Each data point was measured in triplicates. The irradiated and non-irradiated samples were then worked up by taking 90 µL of the mixture and precipitating the protein by adding 180 µL 150 µM benzophenone as internal standard. This resulted in a three-fold dilution of the samples and a final concentration of internal standard of 100 µM. The precipitated protein was removed by centrifugation and the supernatant was analyzed on chiral HPLC (method 2).

The amount of consumed substrate was determined by quantifying the remaining substrate peaks according to the calibration curves shown below and subtracting the obtained values from $[S]_0$ (which was determined for every point individually from the non-irradiated samples). From these values, k_{obs} was determined according to:

$$k_{obs}[h^{-1}] = \frac{\frac{c(consumed\ substrate)[\mu M]}{irradiation\ time\ [h]}}{c(enzyme)[\mu M]} * dilution\ factor\ for\ HPLC$$

Since $[S]_0$ was determined for each point individually, the values varied slightly between triplicates. To be able to average the triplicates, they were therefore normalized for the intended $[S]_0$ according to:

$$k_{obs,corrected}[h^{-1}] = k_{obs,determined}[h^{-1}] * \frac{[S]_{0,intended}\ [\mu M]}{[S]_{0,determined}\ [\mu M]}$$

The corrected values for k_{obs} were then used to determine the kinetic parameters k_{cat} and K_M by fitting the data with the Michaelis Menten equation, with $[E]_0$ = enzyme concentration and $[S]_0$ = substrate concentration:

$$k_{obs,corrected} = \frac{v}{[E]_0} = \frac{k_{cat}\ [S]_0}{K_M + [S]_0}$$

5.1.9. Double-mutant cycle analysis

To quantify the epistasis of the two individual point mutations, a double-mutant cycle analysis was performed. For this, the individual k_{cat}/K_M parameters for (*R,R*)-**4** were determined in Michaelis-Menten kinetics experiments and the interaction free energy $\Delta\Delta G^{\ddagger}_{int}$ was calculated according to:

$$\Delta\Delta G^{\ddagger}_{int} = \Delta G^{\ddagger}_{PLZ2.3} - \Delta G^{\ddagger}_{PLZ2.1} - \Delta G^{\ddagger}_{PLZ2.2} + \Delta G^{\ddagger}_{PLZ2.0}$$

Where

$$\Delta G^{\ddagger}_{enzyme} = -RTln\left(\frac{k_{cat,enzyme,(R,R)\text{-}\mathbf{4}}}{K_{M,enzyme,(R,R)\text{-}\mathbf{4}}}\right)$$

Using

$$R = 1.987\frac{cal}{mol * K}$$

$$T = 298\ K$$

If $\Delta\Delta G^{\ddagger}_{int} = 0$: Both mutations act independently. If $\Delta\Delta G^{\ddagger}_{int} > 0$: The double mutant performs worse than expected for a simple additive interaction, indicating antagonistic epistasis. If $\Delta\Delta G^{\ddagger}_{int} < 0$: The double mutant performs better than expected for a simple additive interaction, indicating synergistic epistasis.

5.1.10. Calibration curves for the quantification of substrates and products from HPLC peaks

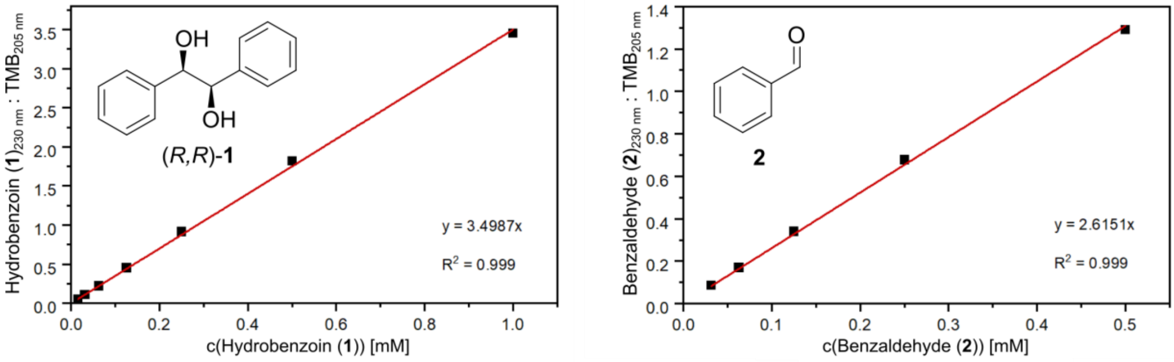

Figure S29: Calibration curves for the quantification of substrates and products via UV absorption, using TMB as the internal standard in HPLC runs. All data points were measured as triplicates. TMB: 1,2,3-Trimethoxybenzene; BA: Benzaldehyde.

The substrate concentrations were measured relative to the area of 100 µM benzophenone as the internal standard. To determine the relative content of (*R*,*R*)- and (*S*,*S*)-**4** in relation to *meso*-**4**, the NMR spectrum of the mixture of **4** was integrated (Figure S30).

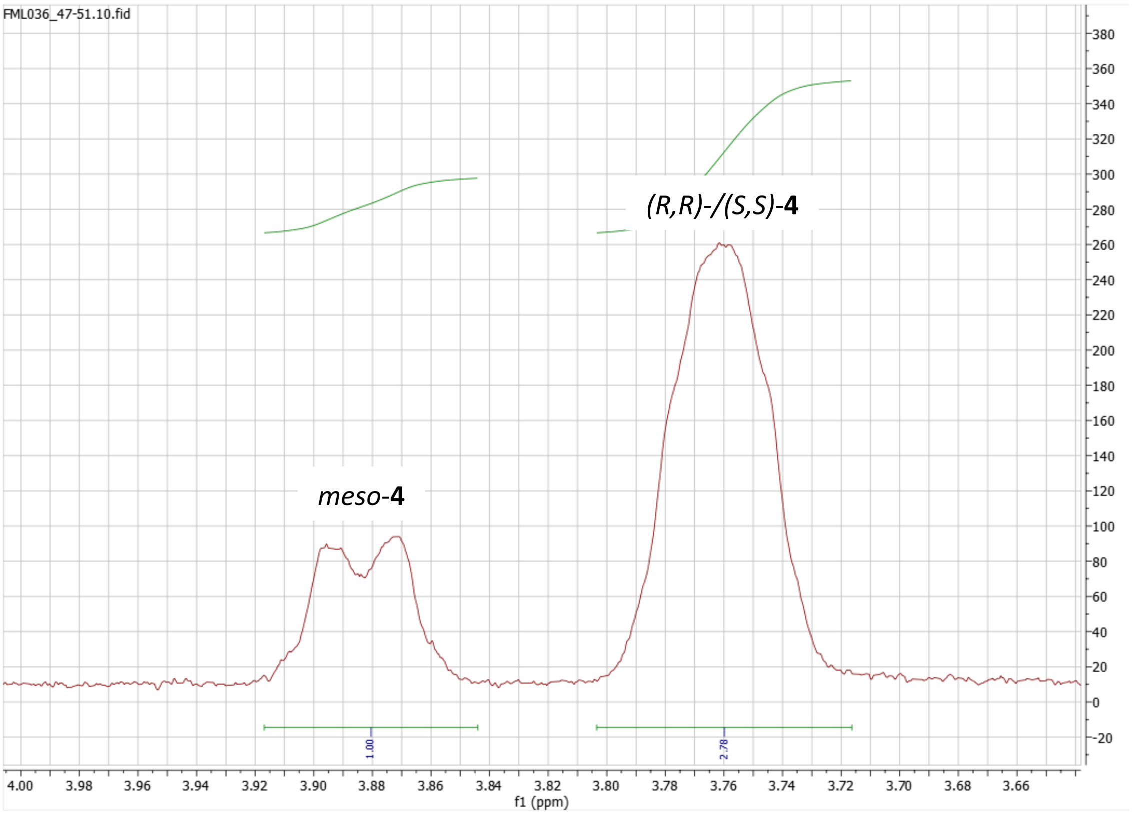

Figure S30: Zoom into the NMR spectrum of substrate **4**. The peak of (*R*,*R*)- **4** and (*S*,*S*)-**4** in this region is nicely separated from the peak of *meso*-**4**, allowing their individual integration to determine the amount of *meso*-**4** in the mixture of all stereoisomers.

The NMR spectrum indicated a ratio of 1 : 2.78 between *meso*-**4** and the combined (*R*,*R*)- and (*S*,*S*)-**4**. The mixture of all stereoisomers therefore contains 26.5% *meso*-**4**, 36.8% (*S*,*S*)-**4**, and 36.8% (*R*,*R*)-**4**. The integrals of the peaks on chiral HPLC (method 2) of the mixture of all stereoisomers of **4** was adjusted accordingly (e.g. 1 mM of the substrate mixture only contains 368 µM (*R*,*R*)-**4**). Following this method, the calibration curves seen in Figure S31 were obtained.

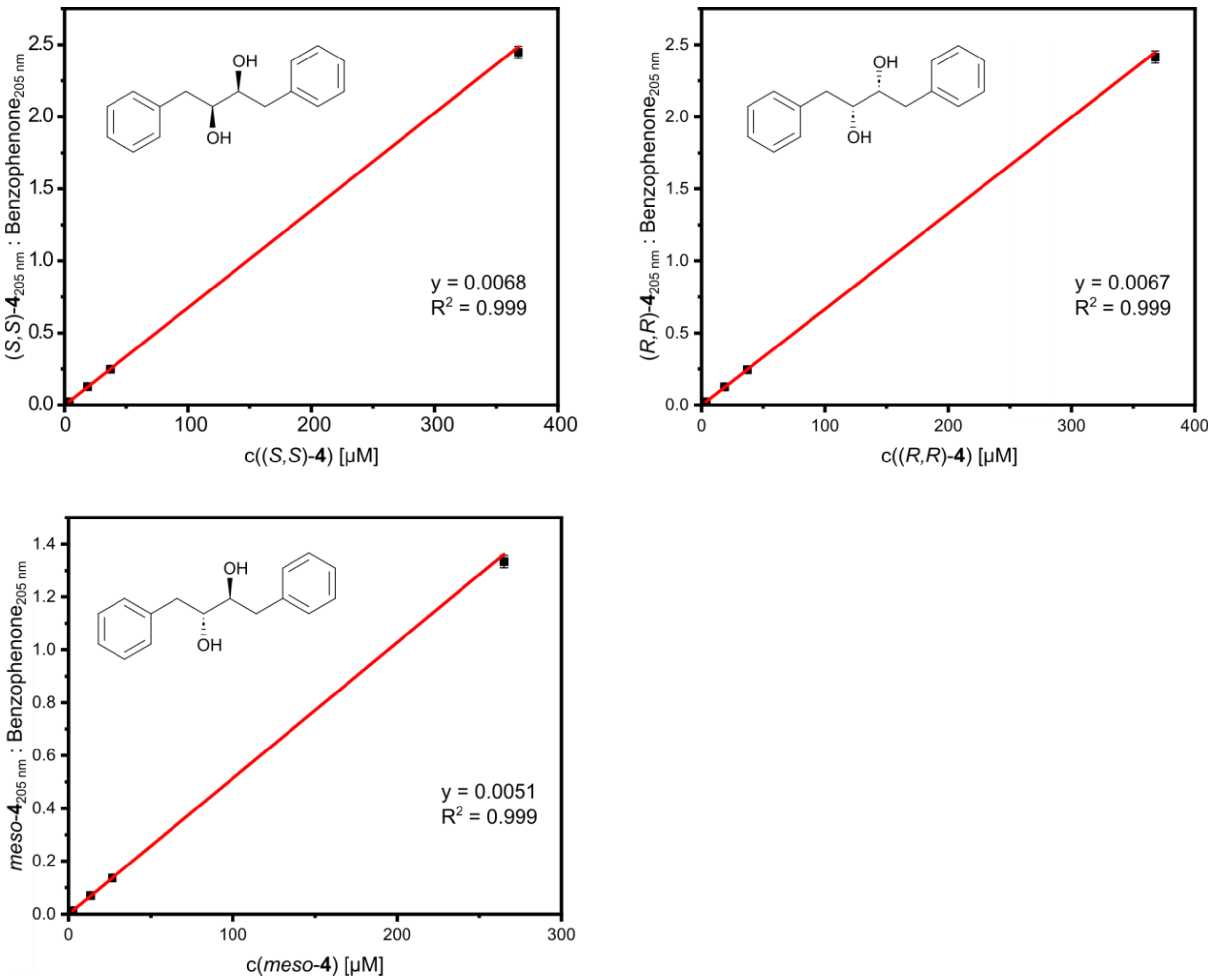

Figure S31: Calibration curves for the individual enantiomers of **4**.

6. Crystallization and X-ray structure determination of PLZ2.3

Ce(III)-bound PLZ2.3 (12 mg/mL) was mixed with 660 µM substrate *rac*-**4** (20 mM stock solution in acetonitrile) and incubated at 4 °C for 3 days. The protein was then pipetted by the Crystal Gryphon protein crystallization pipettor (Art Robbins Instruments) and crystallized within 10 days in sitting drop vapor diffusion crystallization experiments at 20 °C. Crystallization drops had a volume of 0.4 µL with a 3:1 ratio of protein to reservoir solution (0.1 M sodium acetate pH 4.6, 2.0 M sodium chloride), and were equilibrated against 50 µL of reservoir solution. The crystals were soaked in 1 µL of a 1:1 mixture of mother liquor and 2 M lithium sulfate and flash frozen in liquid nitrogen.

Diffraction images were recorded at using synchrotron radiation of λ = 1.060 Å at the P13 beamline of PETRAIII at DESY (Deutsches Elektronen-Synchrotron, EMBL, Hamburg, Germany). Reflections were processed by the XDS suite and data was reduced by XSCALE.[28, 29] The initial model was phased using PHASER[30] and a PLZ2.3 model predicted by AlphaFold3.[31] The model was then built by iterative cycles of restrained refinement using REFMAC5[32] and PHENIX (v. 1.21.2)[33, 34] and model building in COOT (v. 0.9).[35] Water molecules were placed by ARP/wARP 8.0[36] and the structure was completed by TLS refinement with REFMAC5.[31] The geometry of the final structure was analyzed by the MOLPROBITY[37] tool, and the structure was deposited in the RCSB Protein Data Bank (Table S3).

Table S3: Crystallographic data collection and refinement statistics.

	**PLZ2.3 + Ce(III)**
**Crystal parameters**	
Space group	P43
Cell constants	a = 93.2 Å
	b = 93.2 Å
	c = 51.2 Å
**Data collection**	
Beam line	P13, PETRAIII, DESY
Wavelength (Å)	1.060
Resolution range (Å)[b]	30-2.1
	(2.2-2.1)
No. observations	70,360
No. unique reflections[c]	24,843
Completeness (%)[b]	95.7 (97.9)
R_{merge} (%)[b,d]	7.8 (78.2)
I/σ (I)[b]	7.7 (1.6)
**Refinement (REFMAC5)**	
Resolution range (Å)	30-2.1
No. refl. working set	23592
No. refl. test set	1242
No. non hydrogen	2568
No. of ligand atoms	1
Solvent	84
R_{work}/R_{free} (%)[e]	24.1 / 17.9
r.m.s.d. bond (Å) / angle (°)[f]	0.004 / 1.24
Average B-factor (Å^2)	49.6
Ramachandran Plot (%)[g]	99.1 / 0.9 / 0
PDB accession code	**9SZU**

[a] Asymmetric unit

[b] The values in parentheses for resolution range, completeness, R_{merge} and I/σ (I) correspond to the highest resolution shell

[c] Data reduction was carried out with XDS and from a single crystal. Friedel pairs were treated as identical reflections

[d] $R_{merge}(I) = \Sigma_{hkl}\Sigma_j \mid I(hkl)_j - \langle I(hkl)\rangle \mid / \Sigma_{hkl} \Sigma_j I(hkl)_j$, where $I(hkl)_j$ is the jth measurement of the intensity of reflection hkl and <I(hkl)> is the average intensity

[e] $R = \Sigma_{hkl} \mid |F_{obs}| - |F_{calc}| \mid / \Sigma_{hkl} |Fobs|$, where R_{free} is calculated without a sigma cut off for a randomly chosen 5% of reflections, which were not used for structure refinement, and R_{work} is calculated for the remaining reflections

[f] Deviations from ideal bond lengths/angles

[g] Percentage of residues in favored region / allowed region / outlier region

7. Chemical methods

7.1. Separation of (*R,R*)-/(*S,S*)-4 from *meso*-4

(*R,R*)-**4** and (*S,S*)-**4** were separated from *meso*-**4** by flash chromatography on a Biotage Sfär C18 6 g column (Biotage, Uppsala, Sweden) on a Biotage Selekt automated flash chromatography system (Biotage, Uppsala, Sweden). The diastereomers were separated by running an isocratic elution with 25% solvent A (0.1% TFA in H_2O) and 75% solvent B (0.1% TFA in acetonitrile). The eluted fractions were analyzed using HPLC method 2 (chiral column) and the fractions that mostly contained (*R,R*)- and (*S,S*)-**4** were pooled. The solvent was evaporated and the diol was stored at -20 °C. The separation is shown in Figure S13.

7.2. Chemical syntheses

7.2.1. Synthesis of 1,4-diphenylbutane-2,3-diol (4)

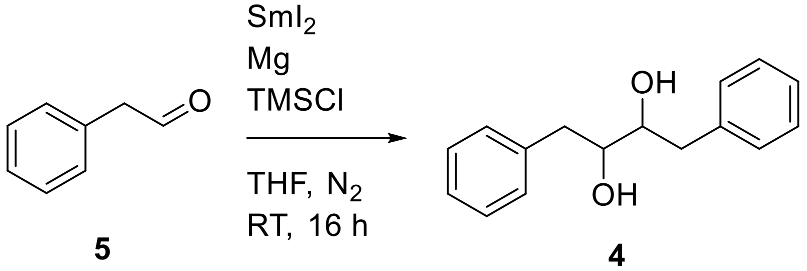

5 **4**

1,4-Diphenylbutane-2,3-diol (**4**) was synthesized following a protocol by *Nomura et al.* (1996).[38] In a *Schlenk* flask under nitrogen atmosphere Mg turnings (8 equiv, 0.78 g, 32 mmol) were stirred for 1 h to activate. 0.1 equiv of SmI_2 (0.1 M in THF, 4 mL, 0.4 mmol) and 0.5 equiv TMSCl (250 µL, 2 mmol) were added. 0.48 g (4 mmol) 2-phenylacetaldehyde (**5**) was mixed with 1 equiv TMSCl (510 µL, 4 mmol) and added dropwise. Upon addition, the SmI_2 mixture turned from blue to grey. The next drop of aldehyde was added when the solution had returned to blue. Due to the increasingly slow regeneration of SmI_2, a syringe pump was used to slowly add the aldehyde at a rate of ca. 0.1 mL/h.

After 16 h 1 M HCl (30 mL) was added. The mixture was extracted with ethyl acetate twice (40 mL), washed with brine and dried over Na_2SO_4. The combined organic phases were concentrated under reduced pressure. 1,4-diphenylbutane-2,3-diol (**4**) was obtained after column chromatography on silica gel (petrol ether/ethyl acetate, 85:15 to 70:30) as a white solid (155 mg, 0.64 mmol, 32%) as a mixture of all possible stereoisomers.

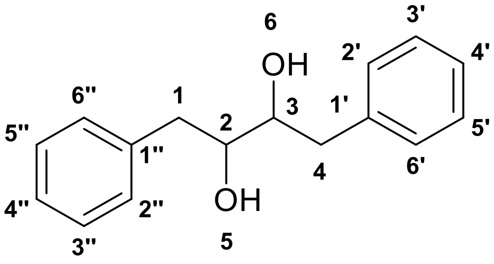

4

^{1}H-NMR (300 MHz, $CDCl_3$): δ [ppm] = 7.40–7.16 (m, 10H, 2′-, 2″-, 3′-, 3″-, 4′-, 4″-, 5′-, 5″-, 6′-, 6″-H), 3.94–3.58 (m, 2H, 2-, 3-H), 3.08–2.73 (m, 4H, 1-, 4-H), 2.09 (s, 2H, 5-OH, 6-OH); **^{13}C-NMR** (101 MHz, $CDCl_3$): δ [ppm] = 138.1 (C-1′, -1″), 129.4 + 128.6 + 126.6 (C-2′, -2″, -3′, -3″, -4′, -4″, -5′, -5″, -6′, -6″), 74.8 and 74.0 (C-2, -3), 40.4 and 38.6 (C-1, -4).

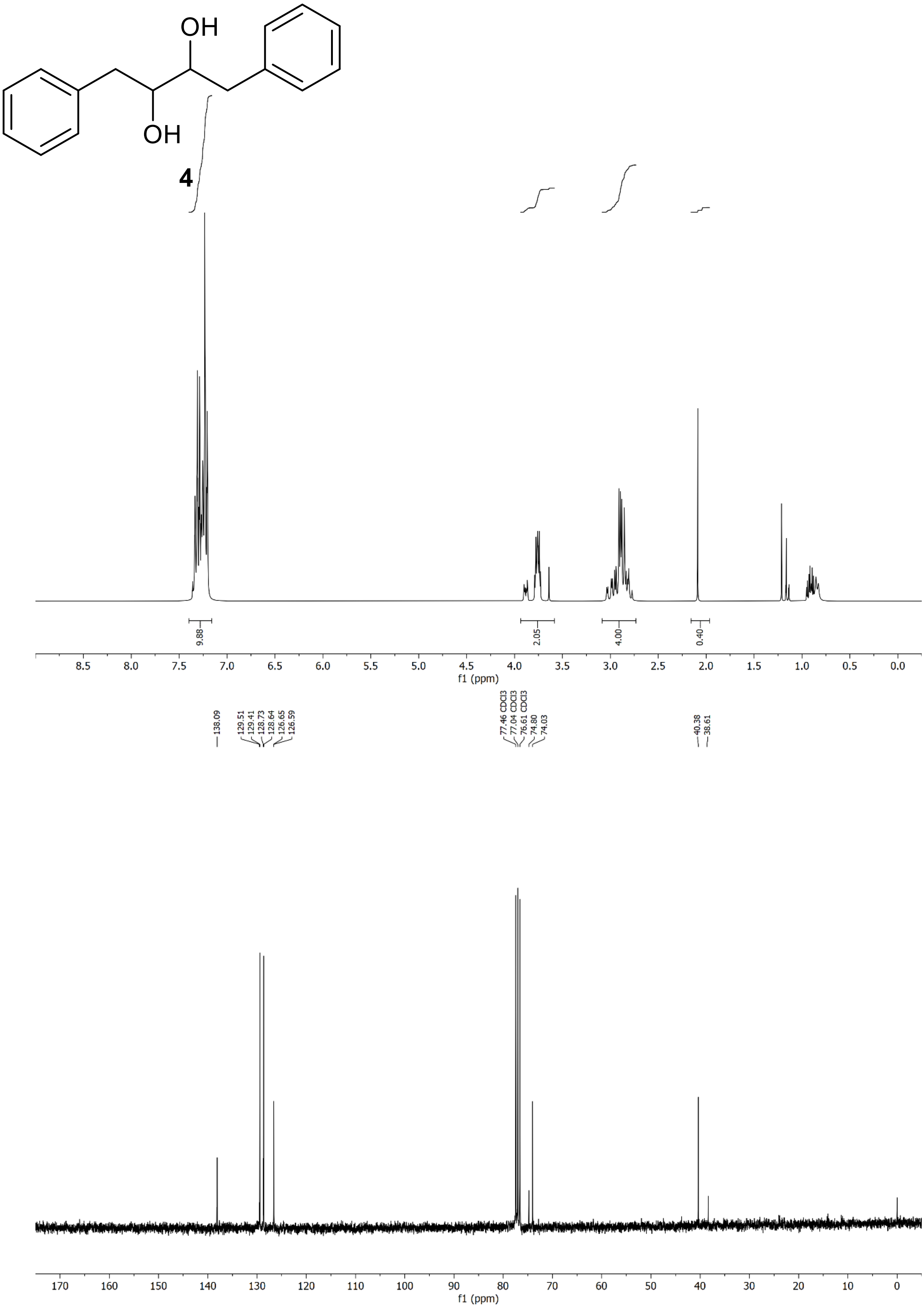

Figure S32: ^{1}H- and ^{13}C-NMR-spectra of diol **4** (mixture of stereoisomers) in $CDCl_3$ (300 MHz/75 MHz).

8. HPLC standards of used compounds

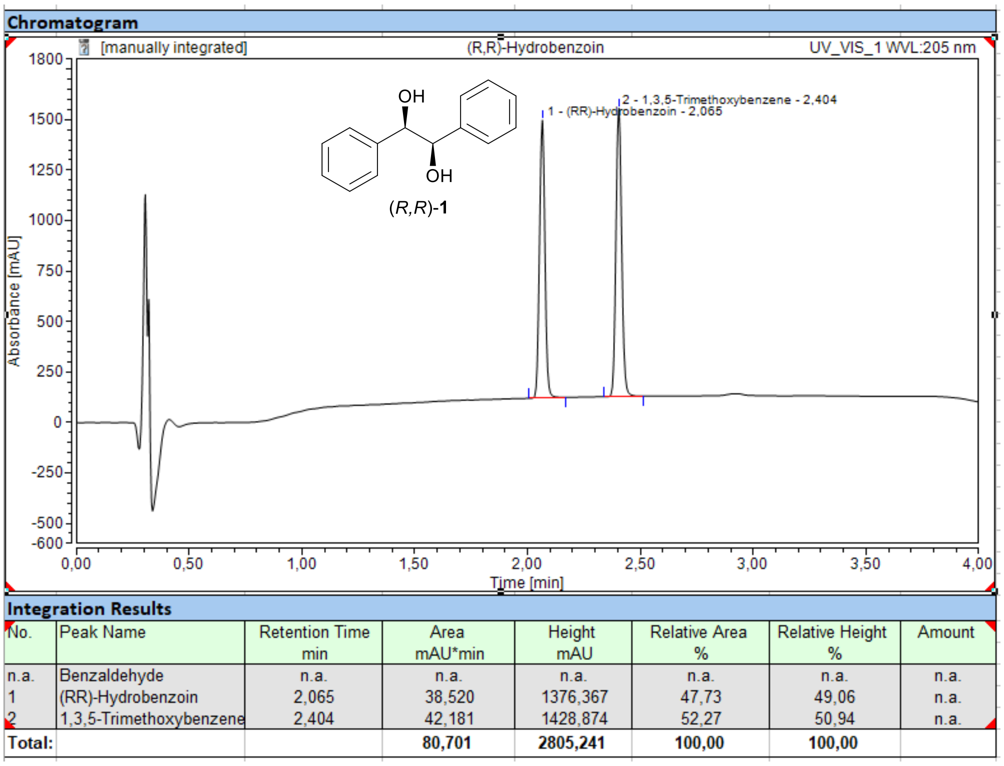

Integration Results

No.	Peak Name	Retention Time min	Area mAU*min	Height mAU	Relative Area %	Relative Height %	Amount
n.a.	Benzaldehyde	n.a.	n.a.	n.a.	n.a.	n.a.	n.a.
1	(RR)-Hydrobenzoin	2,065	38,520	1376,367	47,73	49,06	n.a.
2	1,3,5-Trimethoxybenzene	2,404	42,181	1428,874	52,27	50,94	n.a.
**Total:**			**80,701**	**2805,241**	**100,00**	**100,00**	

Figure S33: HPLC chromatogram of (*R,R*)-hydrobenzoin, (*R,R*)-**1** at 205 nm; Method 1 (achiral, Hypersil Gold C18)

Chromatogram

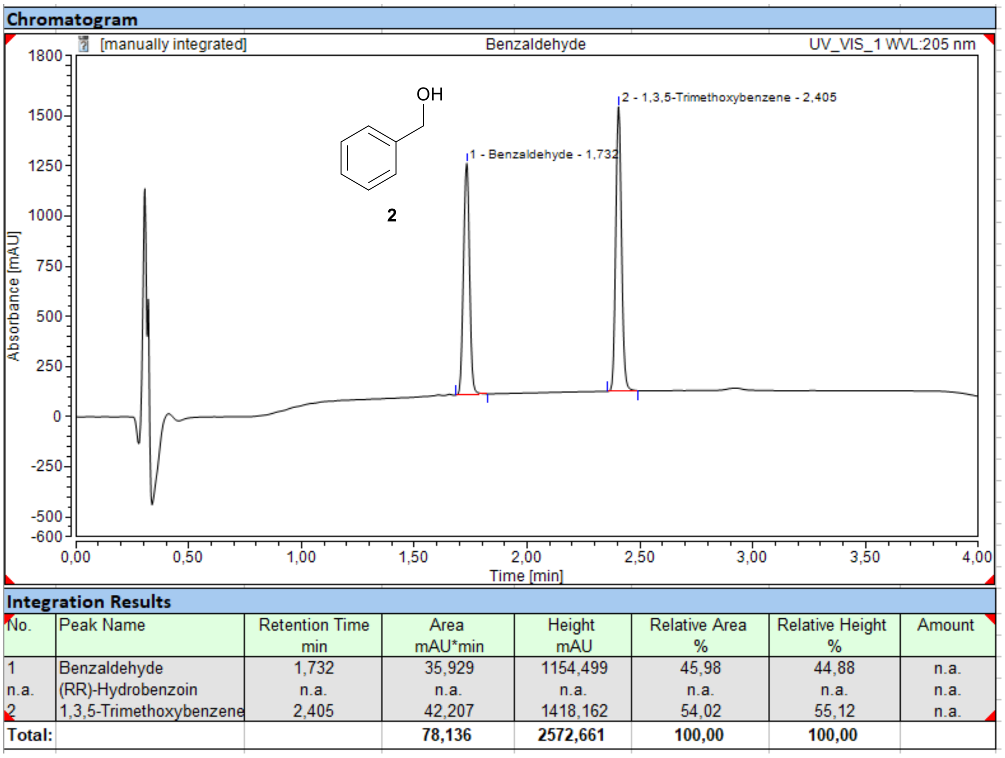

Integration Results

No.	Peak Name	Retention Time min	Area mAU*min	Height mAU	Relative Area %	Relative Height %	Amount
1	Benzaldehyde	1,732	35,929	1154,499	45,98	44,88	n.a.
n.a.	(RR)-Hydrobenzoin	n.a.	n.a.	n.a.	n.a.	n.a.	n.a.
2	1,3,5-Trimethoxybenzene	2,405	42,207	1418,162	54,02	55,12	n.a.
**Total:**			**78,136**	**2572,661**	**100,00**	**100,00**	

Chromatogram

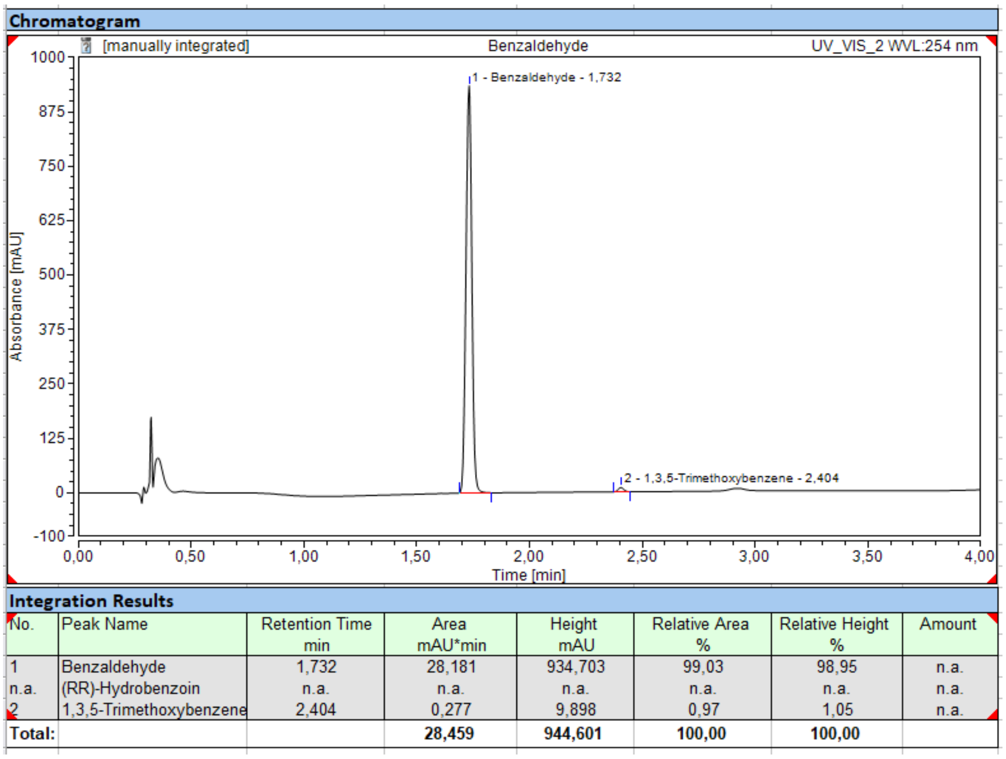

Integration Results

No.	Peak Name	Retention Time min	Area mAU*min	Height mAU	Relative Area %	Relative Height %	Amount
1	Benzaldehyde	1,732	28,181	934,703	99,03	98,95	n.a.
n.a.	(RR)-Hydrobenzoin	n.a.	n.a.	n.a.	n.a.	n.a.	n.a.
2	1,3,5-Trimethoxybenzene	2,404	0,277	9,898	0,97	1,05	n.a.
**Total:**			**28,459**	**944,601**	**100,00**	**100,00**	

Figure S34: HPLC chromatograms of benzaldehyde **2** at 205 nm (top) and 254 nm (bottom) ; Method 1 (achiral, Hypersil Gold C18)

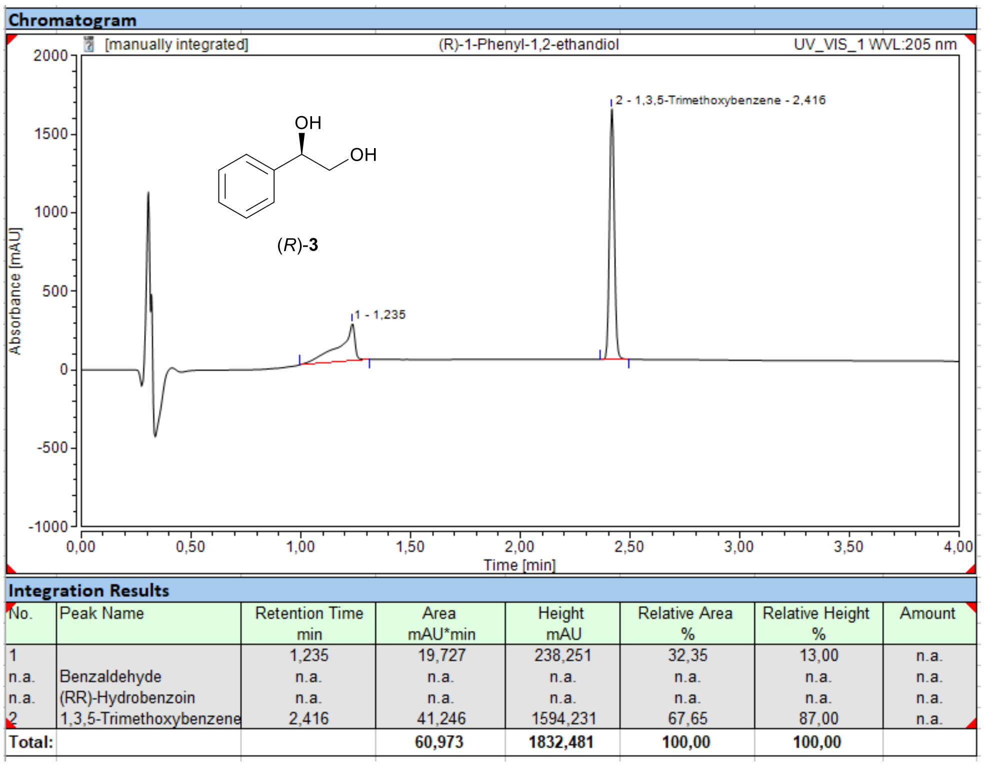

Integration Results

No.	Peak Name	Retention Time min	Area mAU*min	Height mAU	Relative Area %	Relative Height %	Amount
1		1,235	19,727	238,251	32,35	13,00	n.a.
n.a.	Benzaldehyde	n.a.	n.a.	n.a.	n.a.	n.a.	n.a.
n.a.	(RR)-Hydrobenzoin	n.a.	n.a.	n.a.	n.a.	n.a.	n.a.
2	1,3,5-Trimethoxybenzene	2,416	41,246	1594,231	67,65	87,00	n.a.
**Total:**			**60,973**	**1832,481**	**100,00**	**100,00**	

Figure S35: HPLC chromatogram of substrate (*R*)-**3** at 205 nm; Method 1 (achiral, Hypersil Gold C18)

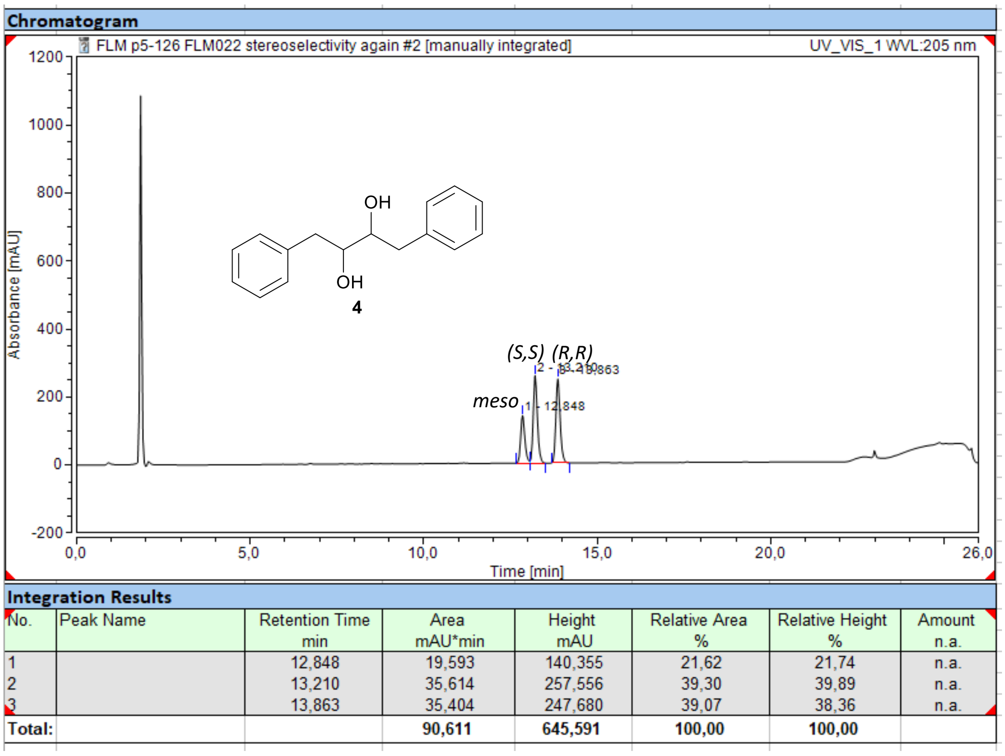

Integration Results

No.	Peak Name	Retention Time min	Area mAU*min	Height mAU	Relative Area %	Relative Height %	Amount n.a.
1		12,848	19,593	140,355	21,62	21,74	n.a.
2		13,210	35,614	257,556	39,30	39,89	n.a.
3		13,863	35,404	247,680	39,07	38,36	n.a.
**Total:**			**90,611**	**645,591**	**100,00**	**100,00**	

Figure S36: HPLC chromatogram of substrate rac-**4** at 205 nm; Method 2 (chiral, Chiralcel OJ-RH)

9. HPLC chromatograms of the substrate scope / yield determination

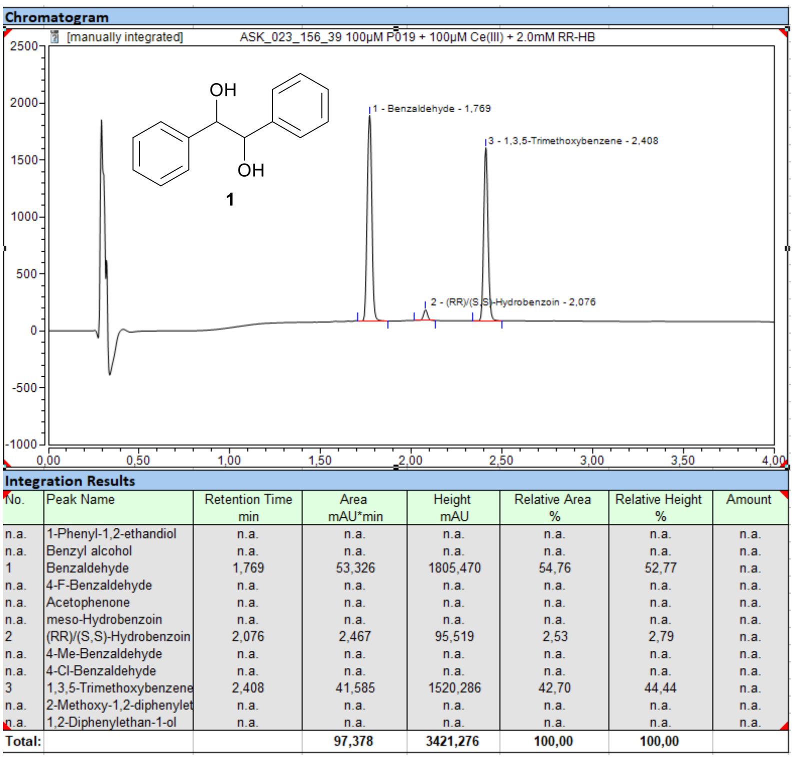

Integration Results

No.	Peak Name	Retention Time min	Area mAU*min	Height mAU	Relative Area %	Relative Height %	Amount
n.a.	1-Phenyl-1,2-ethandiol	n.a.	n.a.	n.a.	n.a.	n.a.	n.a.
n.a.	Benzyl alcohol	n.a.	n.a.	n.a.	n.a.	n.a.	n.a.
1	Benzaldehyde	1,769	53,326	1805,470	54,76	52,77	n.a.
n.a.	4-F-Benzaldehyde	n.a.	n.a.	n.a.	n.a.	n.a.	n.a.
n.a.	Acetophenone	n.a.	n.a.	n.a.	n.a.	n.a.	n.a.
n.a.	meso-Hydrobenzoin	n.a.	n.a.	n.a.	n.a.	n.a.	n.a.
2	(RR)/(S,S)-Hydrobenzoin	2,076	2,467	95,519	2,53	2,79	n.a.
n.a.	4-Me-Benzaldehyde	n.a.	n.a.	n.a.	n.a.	n.a.	n.a.
n.a.	4-Cl-Benzaldehyde	n.a.	n.a.	n.a.	n.a.	n.a.	n.a.
3	1,3,5-Trimethoxybenzene	2,408	41,585	1520,286	42,70	44,44	n.a.
n.a.	2-Methoxy-1,2-diphenylet	n.a.	n.a.	n.a.	n.a.	n.a.	n.a.
n.a.	1,2-Diphenylethan-1-ol	n.a.	n.a.	n.a.	n.a.	n.a.	n.a.
**Total:**			**97,378**	**3421,276**	**100,00**	**100,00**	

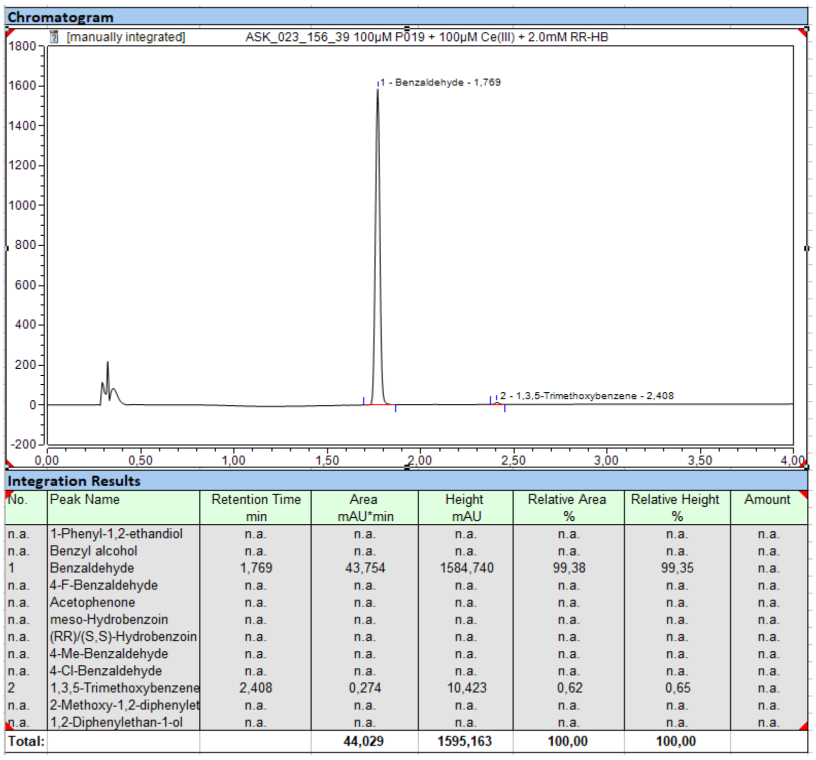

Integration Results

No.	Peak Name	Retention Time min	Area mAU*min	Height mAU	Relative Area %	Relative Height %	Amount
n.a.	1-Phenyl-1,2-ethandiol	n.a.	n.a.	n.a.	n.a.	n.a.	n.a.
n.a.	Benzyl alcohol	n.a.	n.a.	n.a.	n.a.	n.a.	n.a.
1	Benzaldehyde	1,769	43,754	1584,740	99,38	99,35	n.a.
n.a.	4-F-Benzaldehyde	n.a.	n.a.	n.a.	n.a.	n.a.	n.a.
n.a.	Acetophenone	n.a.	n.a.	n.a.	n.a.	n.a.	n.a.
n.a.	meso-Hydrobenzoin	n.a.	n.a.	n.a.	n.a.	n.a.	n.a.
n.a.	(RR)/(S,S)-Hydrobenzoin	n.a.	n.a.	n.a.	n.a.	n.a.	n.a.
n.a.	4-Me-Benzaldehyde	n.a.	n.a.	n.a.	n.a.	n.a.	n.a.
n.a.	4-Cl-Benzaldehyde	n.a.	n.a.	n.a.	n.a.	n.a.	n.a.
2	1,3,5-Trimethoxybenzene	2,408	0,274	10,423	0,62	0,65	n.a.
n.a.	2-Methoxy-1,2-diphenylet	n.a.	n.a.	n.a.	n.a.	n.a.	n.a.
n.a.	1,2-Diphenylethan-1-ol	n.a.	n.a.	n.a.	n.a.	n.a.	n.a.
**Total:**			**44,029**	**1595,163**	**100,00**	**100,00**	

Figure S37: HPLC chromatograms after the reaction of PLZ1.4 with substrate **1** at 205 nm (top) and 254 nm (bottom); Method 1 (achiral, Hypersil Gold C18); dilution factor: 8x

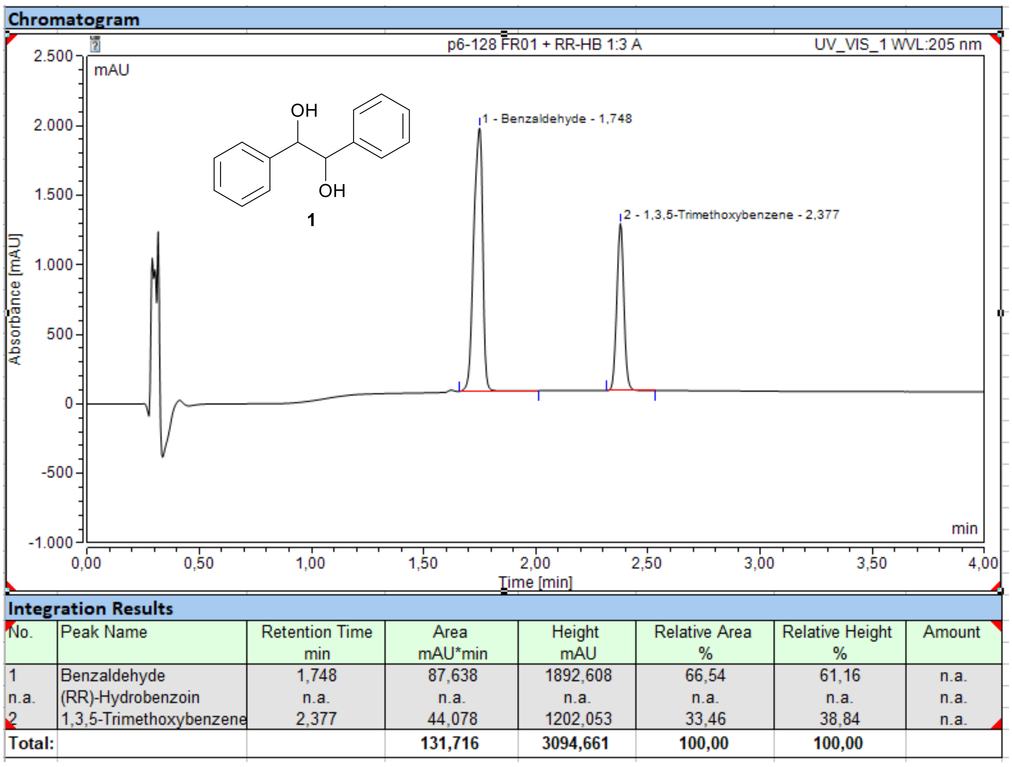

Integration Results

No.	Peak Name	Retention Time<br>min	Area<br>mAU*min	Height<br>mAU	Relative Area<br>%	Relative Height<br>%	Amount
1	Benzaldehyde	1,748	87,638	1892,608	66,54	61,16	n.a.
n.a.	(RR)-Hydrobenzoin	n.a.	n.a.	n.a.	n.a.	n.a.	n.a.
2	1,3,5-Trimethoxybenzene	2,377	44,078	1202,053	33,46	38,84	n.a.
**Total:**			**131,716**	**3094,661**	**100,00**	**100,00**	

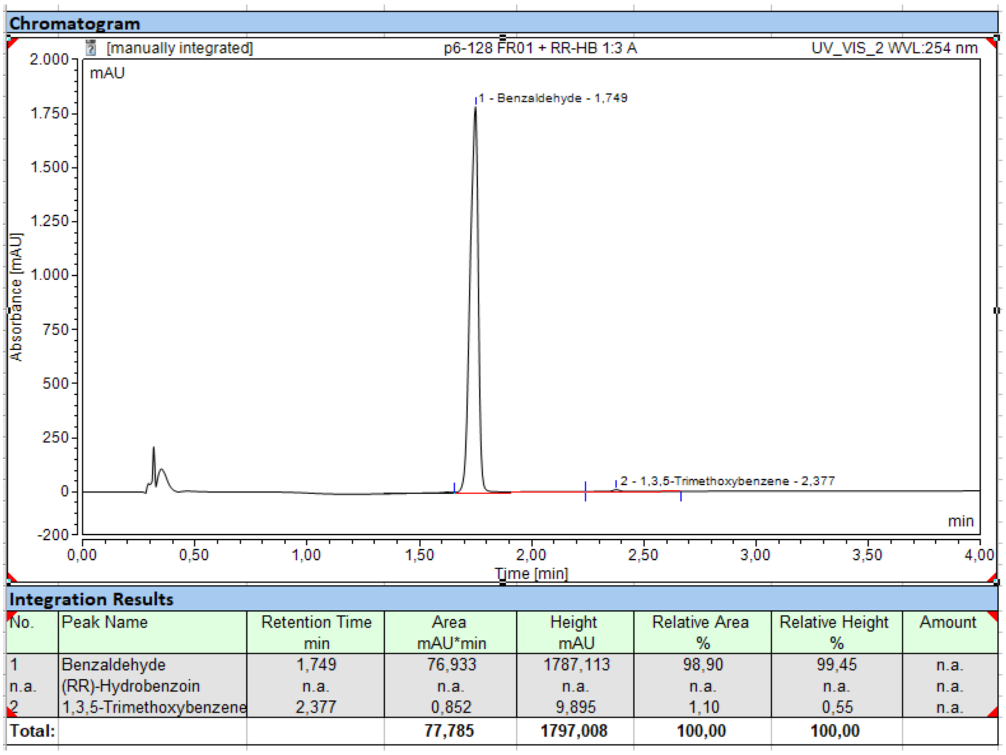

Integration Results

No.	Peak Name	Retention Time<br>min	Area<br>mAU*min	Height<br>mAU	Relative Area<br>%	Relative Height<br>%	Amount
1	Benzaldehyde	1,749	76,933	1787,113	98,90	99,45	n.a.
n.a.	(RR)-Hydrobenzoin	n.a.	n.a.	n.a.	n.a.	n.a.	n.a.
2	1,3,5-Trimethoxybenzene	2,377	0,852	9,895	1,10	0,55	n.a.
**Total:**			**77,785**	**1797,008**	**100,00**	**100,00**	

Figure S38: HPLC chromatograms after the reaction of PLZ2.0 with substrate **1** at 205 nm (top) and 254 nm (bottom); Method 1 (achiral, Hypersil Gold C18); dilution factor: 6x

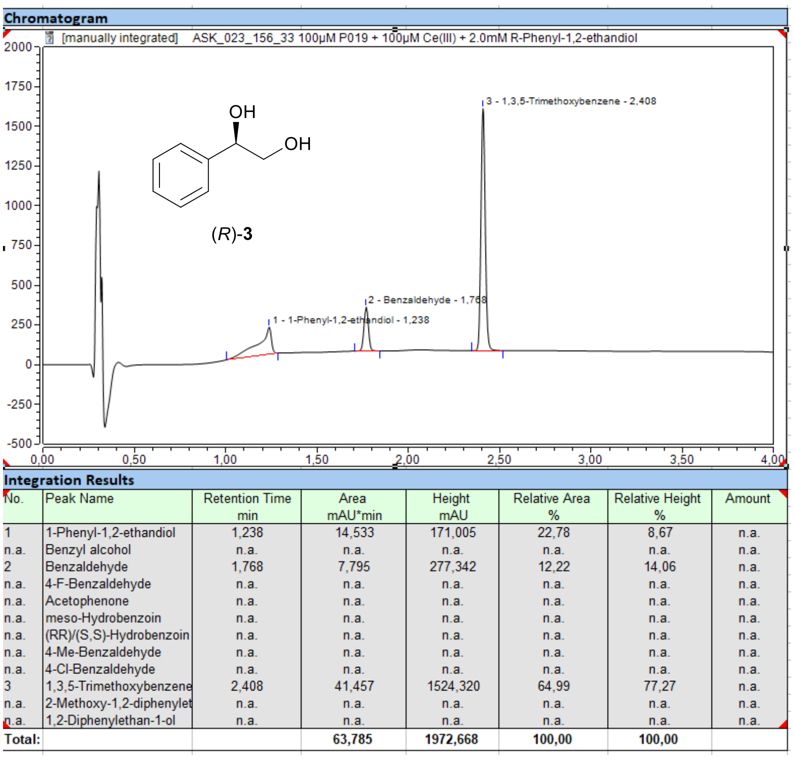

Integration Results

No.	Peak Name	Retention Time min	Area mAU*min	Height mAU	Relative Area %	Relative Height %	Amount
1	1-Phenyl-1,2-ethandiol	1,238	14,533	171,005	22,78	8,67	n.a.
n.a.	Benzyl alcohol	n.a.	n.a.	n.a.	n.a.	n.a.	n.a.
2	Benzaldehyde	1,768	7,795	277,342	12,22	14,06	n.a.
n.a.	4-F-Benzaldehyde	n.a.	n.a.	n.a.	n.a.	n.a.	n.a.
n.a.	Acetophenone	n.a.	n.a.	n.a.	n.a.	n.a.	n.a.
n.a.	meso-Hydrobenzoin	n.a.	n.a.	n.a.	n.a.	n.a.	n.a.
n.a.	(RR)/(S,S)-Hydrobenzoin	n.a.	n.a.	n.a.	n.a.	n.a.	n.a.
n.a.	4-Me-Benzaldehyde	n.a.	n.a.	n.a.	n.a.	n.a.	n.a.
n.a.	4-Cl-Benzaldehyde	n.a.	n.a.	n.a.	n.a.	n.a.	n.a.
3	1,3,5-Trimethoxybenzene	2,408	41,457	1524,320	64,99	77,27	n.a.
n.a.	2-Methoxy-1,2-diphenylet	n.a.	n.a.	n.a.	n.a.	n.a.	n.a.
n.a.	1,2-Diphenylethan-1-ol	n.a.	n.a.	n.a.	n.a.	n.a.	n.a.
**Total:**			**63,785**	**1972,668**	**100,00**	**100,00**	

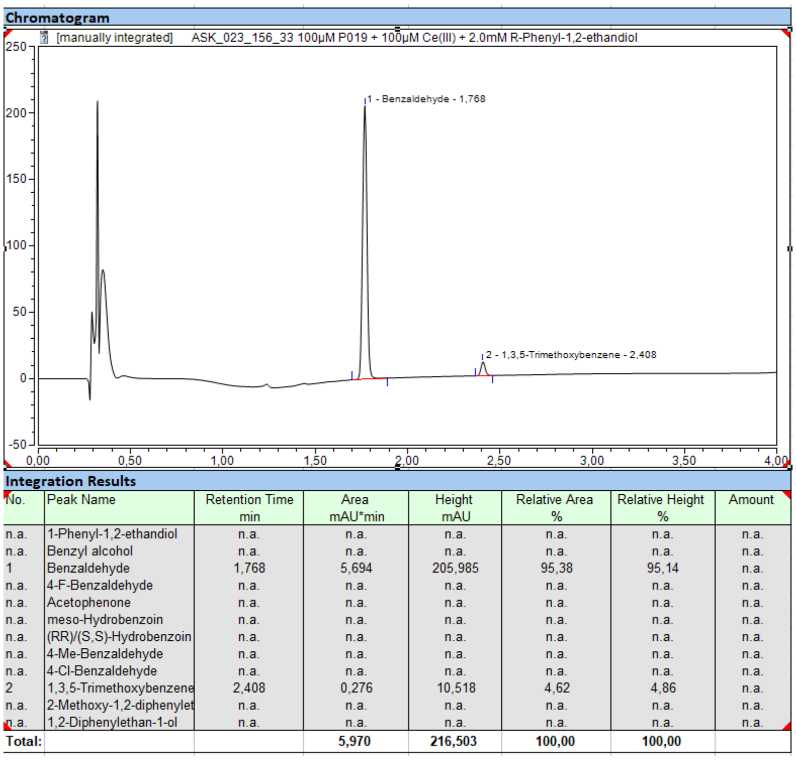

Integration Results

No.	Peak Name	Retention Time min	Area mAU*min	Height mAU	Relative Area %	Relative Height %	Amount
n.a.	1-Phenyl-1,2-ethandiol	n.a.	n.a.	n.a.	n.a.	n.a.	n.a.
n.a.	Benzyl alcohol	n.a.	n.a.	n.a.	n.a.	n.a.	n.a.
1	Benzaldehyde	1,768	5,694	205,985	95,38	95,14	n.a.
n.a.	4-F-Benzaldehyde	n.a.	n.a.	n.a.	n.a.	n.a.	n.a.
n.a.	Acetophenone	n.a.	n.a.	n.a.	n.a.	n.a.	n.a.
n.a.	meso-Hydrobenzoin	n.a.	n.a.	n.a.	n.a.	n.a.	n.a.
n.a.	(RR)/(S,S)-Hydrobenzoin	n.a.	n.a.	n.a.	n.a.	n.a.	n.a.
n.a.	4-Me-Benzaldehyde	n.a.	n.a.	n.a.	n.a.	n.a.	n.a.
n.a.	4-Cl-Benzaldehyde	n.a.	n.a.	n.a.	n.a.	n.a.	n.a.
2	1,3,5-Trimethoxybenzene	2,408	0,276	10,518	4,62	4,86	n.a.
n.a.	2-Methoxy-1,2-diphenylet	n.a.	n.a.	n.a.	n.a.	n.a.	n.a.
n.a.	1,2-Diphenylethan-1-ol	n.a.	n.a.	n.a.	n.a.	n.a.	n.a.
**Total:**			**5,970**	**216,503**	**100,00**	**100,00**	

Figure S39: HPLC chromatograms after the reaction of PLZ1.4 with substrate **3** at 205 nm (top) and 254 nm (bottom); Method 1 (achiral, Hypersil Gold C18); dilution factor: 8x

Chromatogram

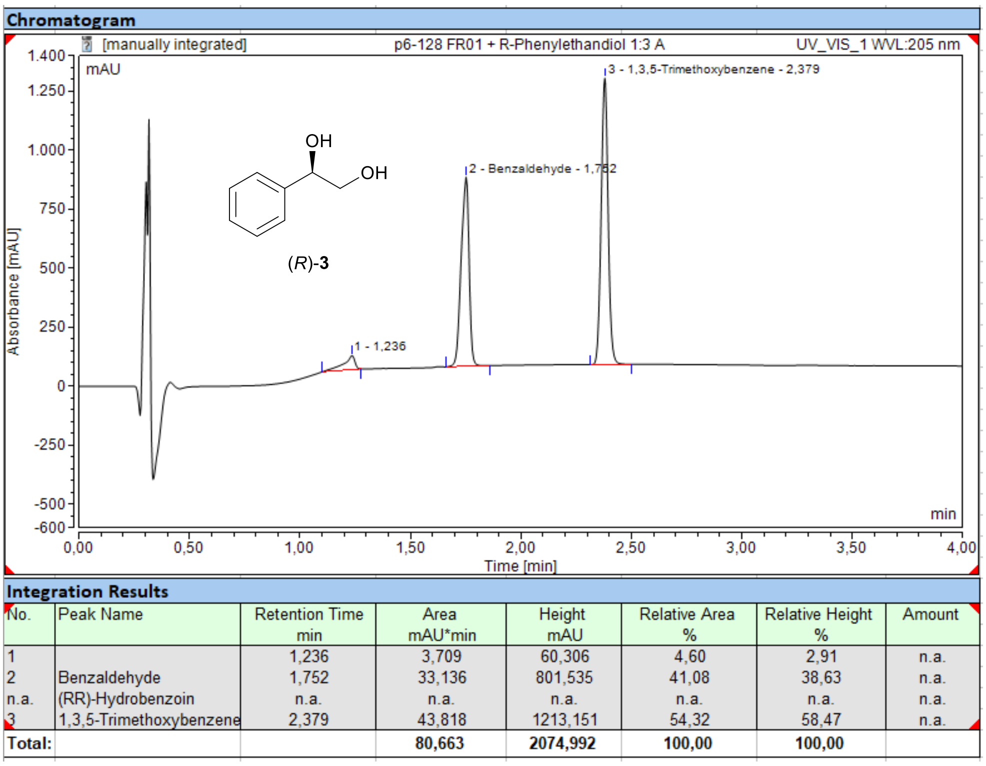

Integration Results

No.	Peak Name	Retention Time<br>min	Area<br>mAU*min	Height<br>mAU	Relative Area<br>%	Relative Height<br>%	Amount
1		1,236	3,709	60,306	4,60	2,91	n.a.
2	Benzaldehyde	1,752	33,136	801,535	41,08	38,63	n.a.
n.a.	(RR)-Hydrobenzoin	n.a.	n.a.	n.a.	n.a.	n.a.	n.a.
3	1,3,5-Trimethoxybenzene	2,379	43,818	1213,151	54,32	58,47	n.a.
**Total:**			**80,663**	**2074,992**	**100,00**	**100,00**	

Chromatogram

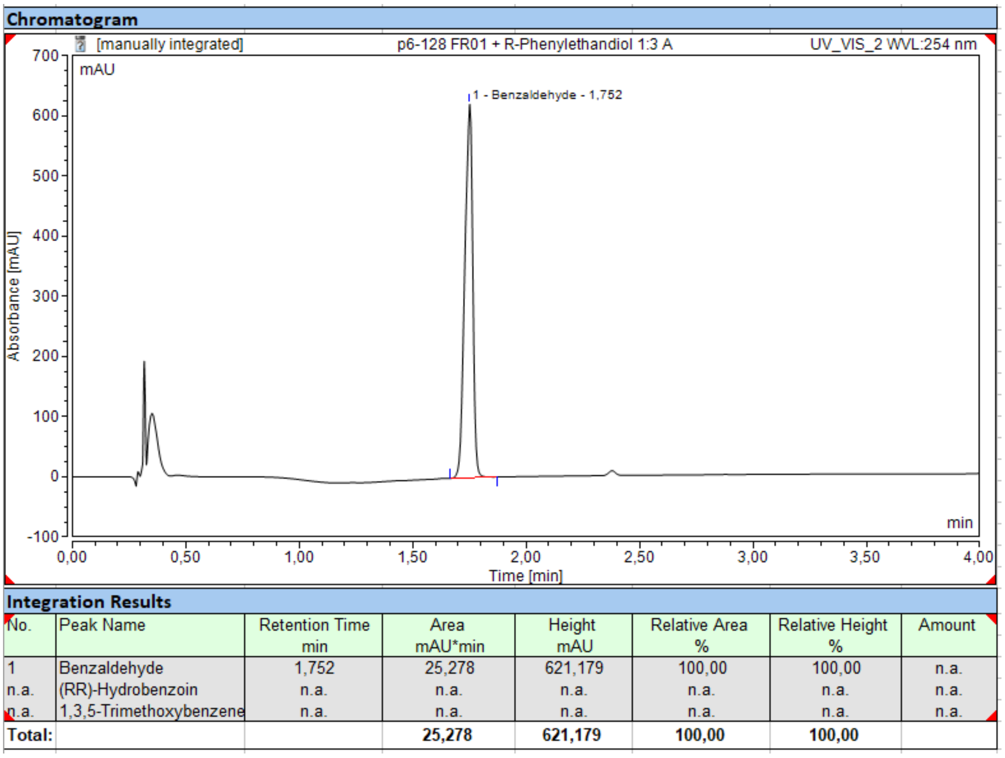

Integration Results

No.	Peak Name	Retention Time<br>min	Area<br>mAU*min	Height<br>mAU	Relative Area<br>%	Relative Height<br>%	Amount
1	Benzaldehyde	1,752	25,278	621,179	100,00	100,00	n.a.
n.a.	(RR)-Hydrobenzoin	n.a.	n.a.	n.a.	n.a.	n.a.	n.a.
n.a.	1,3,5-Trimethoxybenzene	n.a.	n.a.	n.a.	n.a.	n.a.	n.a.
**Total:**			**25,278**	**621,179**	**100,00**	**100,00**	

Figure S40: HPLC chromatograms after the reaction of PLZ2.0 with substrate **3** at 205 nm (top) and 254 nm (bottom); Method 1 (achiral, Hypersil Gold C18); dilution factor: 6x

10. HPLC chromatograms of the selectivity for substrate 1

Chromatogram

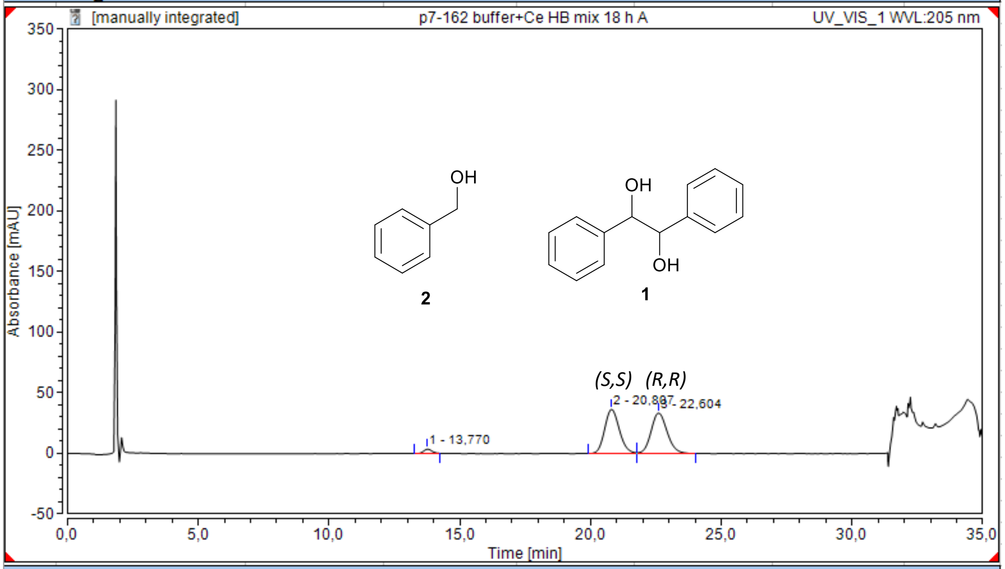

Integration Results

No.	Peak Name	Retention Time min	Area mAU*min	Height mAU	Relative Area %	Relative Height %	Amount n.a.
1		13,770	1,265	3,535	2,59	4,82	n.a.
2		20,807	23,744	36,475	48,72	49,71	n.a.
3		22,604	23,728	33,367	48,69	45,47	n.a.
**Total:**			**48,736**	**73,377**	**100,00**	**100,00**	

Figure S41: HPLC chromatogram after the reaction of substrate rac-**1** with buffer + $CeCl_3$ at 205 nm; Method 3 (chiral, Chiralcel OJ-RH)

Chromatogram

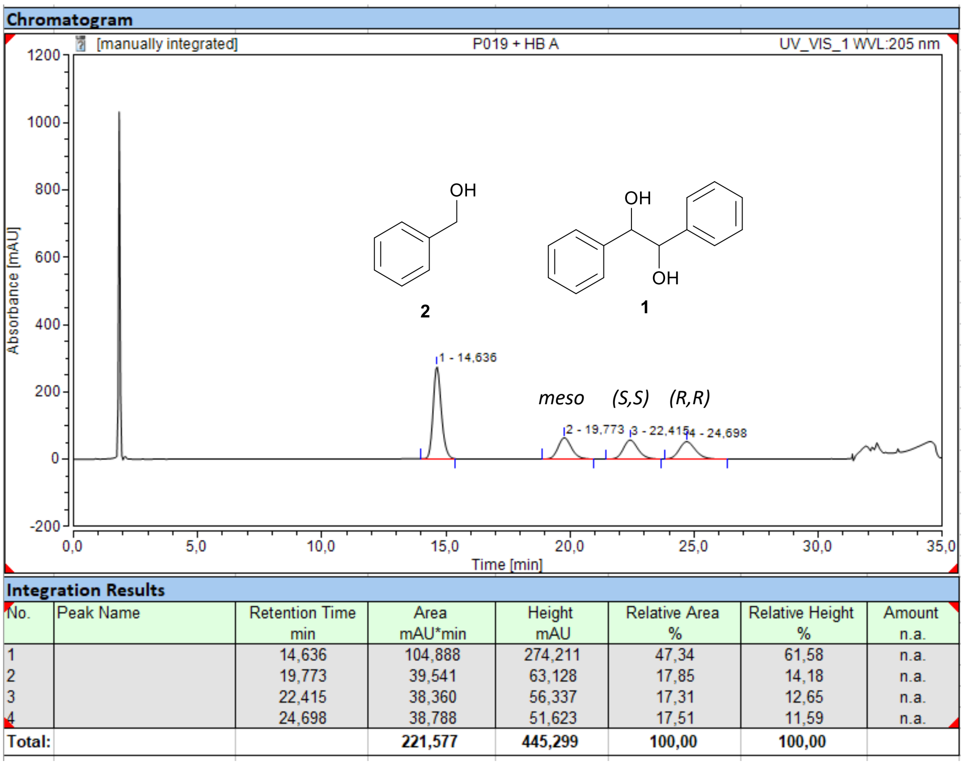

Integration Results

No.	Peak Name	Retention Time min	Area mAU*min	Height mAU	Relative Area %	Relative Height %	Amount n.a.
1		14,636	104,888	274,211	47,34	61,58	n.a.
2		19,773	39,541	63,128	17,85	14,18	n.a.
3		22,415	38,360	56,337	17,31	12,65	n.a.
4		24,698	38,788	51,623	17,51	11,59	n.a.
**Total:**			**221,577**	**445,299**	**100,00**	**100,00**	

Figure S42: HPLC chromatogram after the reaction of substrate rac-**1** with PLZ1.4 at 205 nm; Method 3 (chiral, Chiralcel OJ-RH)

Chromatogram

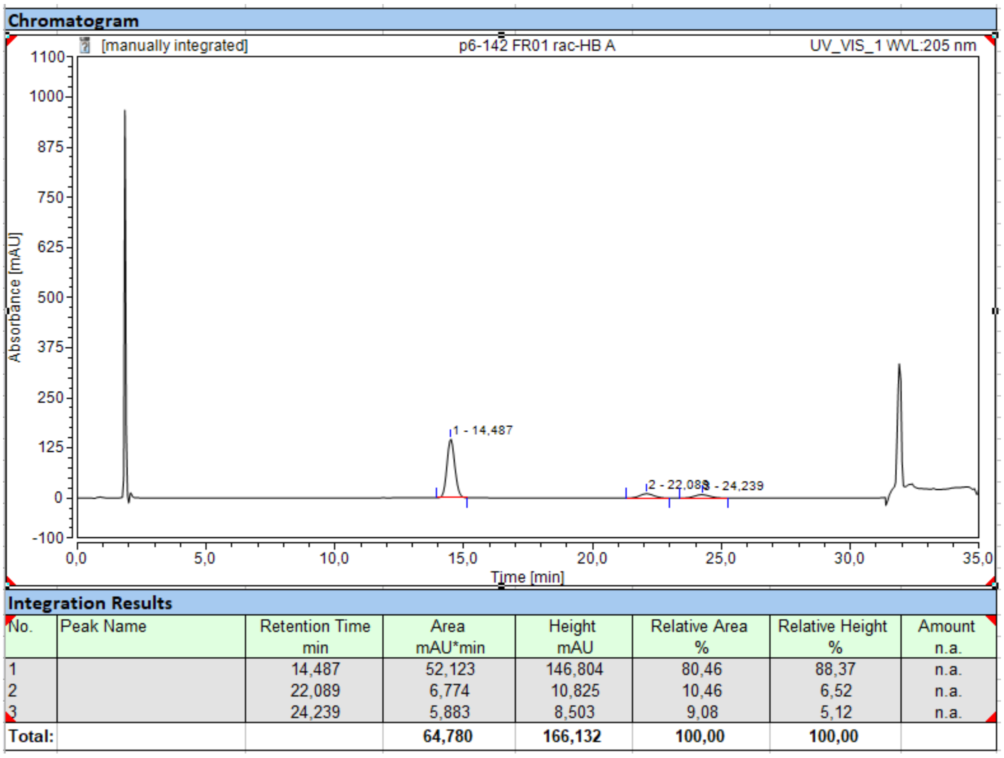

Integration Results

No.	Peak Name	Retention Time min	Area mAU*min	Height mAU	Relative Area %	Relative Height %	Amount n.a.
1		14,487	52,123	146,804	80,46	88,37	n.a.
2		22,089	6,774	10,825	10,46	6,52	n.a.
3		24,239	5,883	8,503	9,08	5,12	n.a.
**Total:**			**64,780**	**166,132**	**100,00**	**100,00**	

Figure S43: HPLC chromatogram after the reaction of substrate rac-**1** with PLZ2.0 at 205 nm; Method 3 (chiral, Chiralcel OJ-RH)

Chromatogram

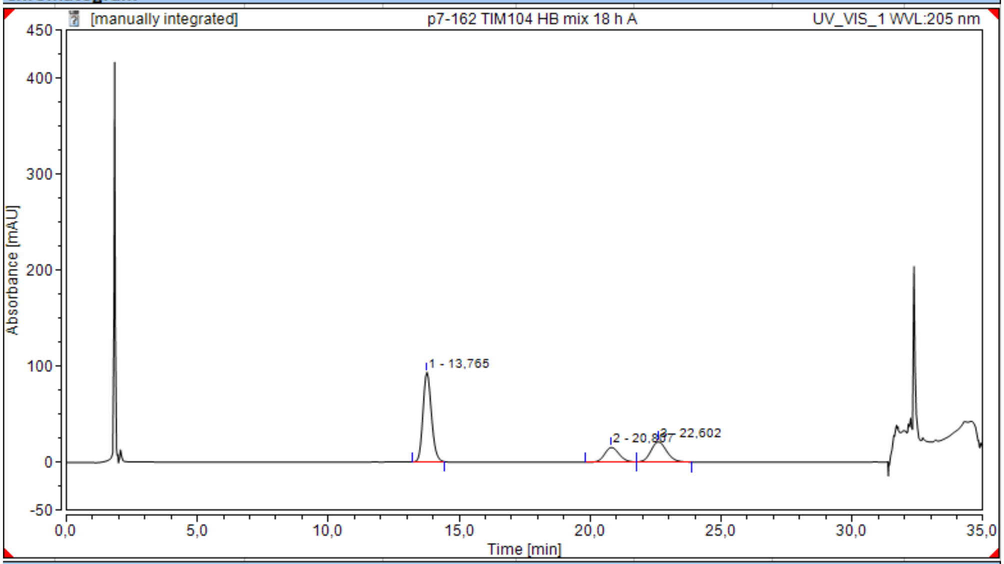

Integration Results

No.	Peak Name	Retention Time min	Area mAU*min	Height mAU	Relative Area %	Relative Height %	Amount n.a.
1		13,765	34,792	93,430	58,41	71,92	n.a.
2		20,807	9,931	15,404	16,67	11,86	n.a.
3		22,602	14,843	21,071	24,92	16,22	n.a.
**Total:**			**59,566**	**129,905**	**100,00**	**100,00**	

Figure S44: HPLC chromatogram after the reaction of substrate rac-**1** with PLZ2.3 at 205 nm; Method 3 (chiral, Chiralcel OJ-RH)

11. HPLC chromatograms of the selectivity for substrate 4

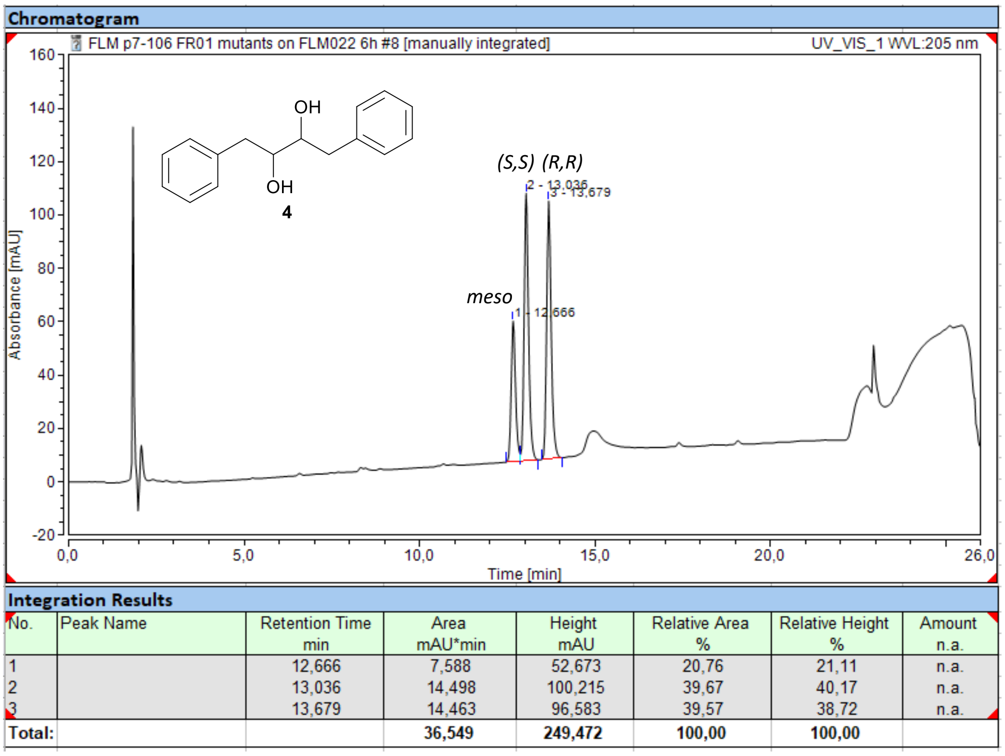

Integration Results

No.	Peak Name	Retention Time min	Area mAU*min	Height mAU	Relative Area %	Relative Height %	Amount n.a.
1		12,666	7,588	52,673	20,76	21,11	n.a.
2		13,036	14,498	100,215	39,67	40,17	n.a.
3		13,679	14,463	96,583	39,57	38,72	n.a.
**Total:**			**36,549**	**249,472**	**100,00**	**100,00**	

Figure S45: HPLC chromatogram after the reaction of substrate rac-**4** with buffer + $CeCl_3$ at 205 nm; Method 2 (chiral, Chiralcel OJ-RH)

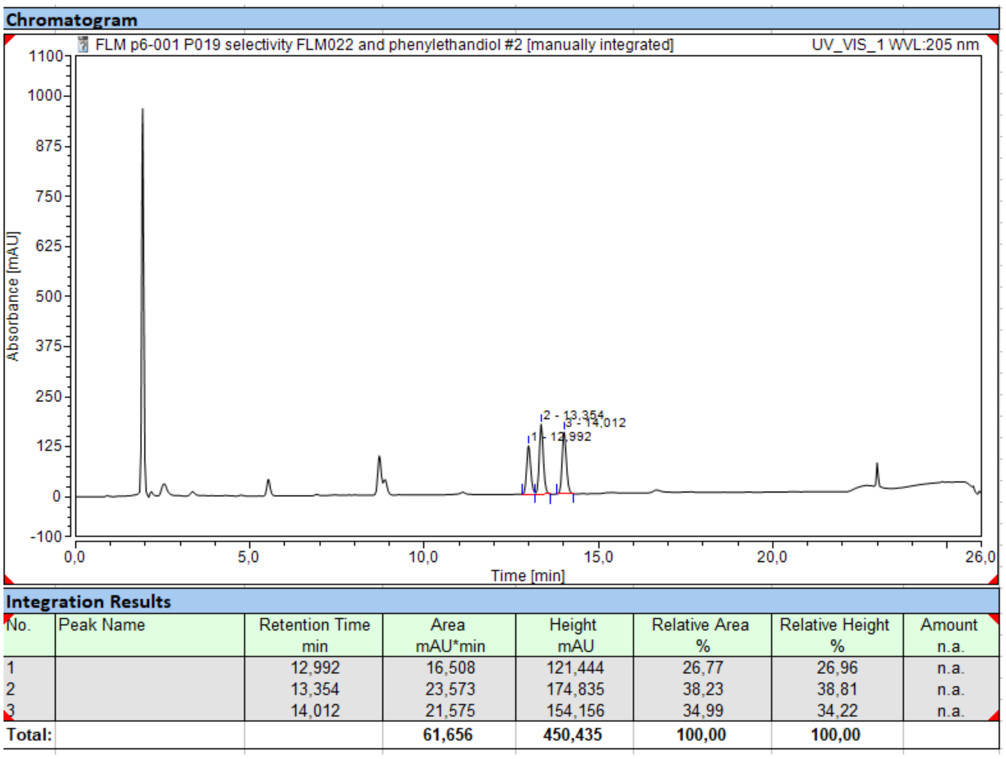

Integration Results

No.	Peak Name	Retention Time min	Area mAU*min	Height mAU	Relative Area %	Relative Height %	Amount n.a.
1		12,992	16,508	121,444	26,77	26,96	n.a.
2		13,354	23,573	174,835	38,23	38,81	n.a.
3		14,012	21,575	154,156	34,99	34,22	n.a.
**Total:**			**61,656**	**450,435**	**100,00**	**100,00**	

Figure S46: HPLC chromatogram after the reaction of substrate rac-**4** with PLZ1.4 at 205 nm; Method 2 (chiral, Chiralcel OJ-RH)

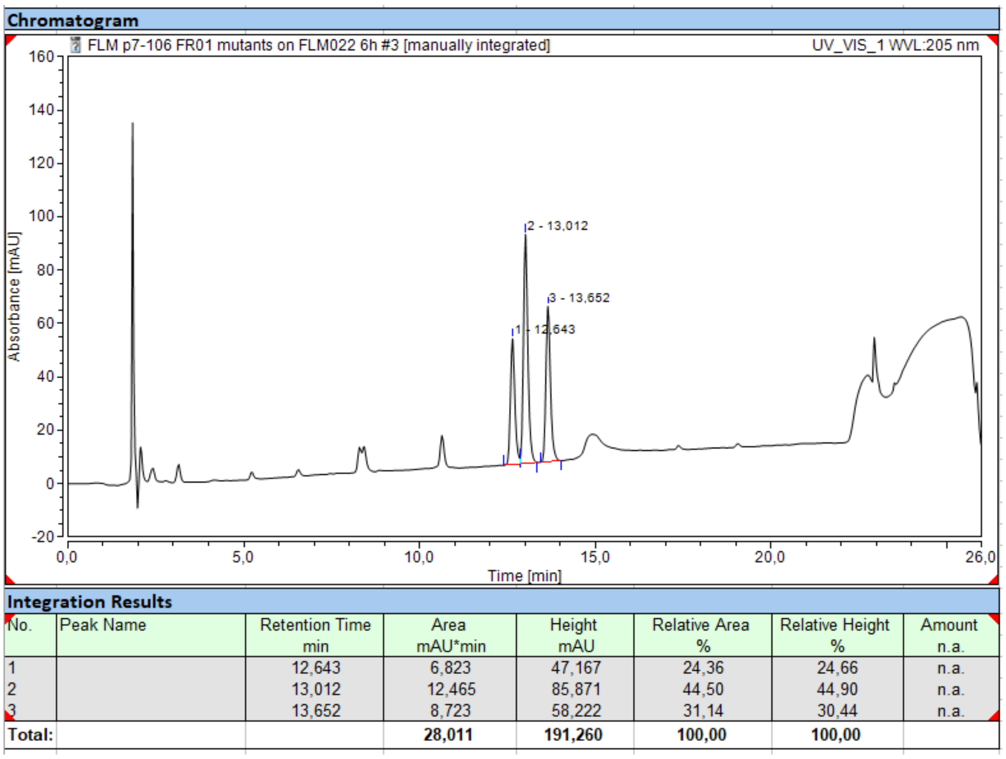

Integration Results

No.	Peak Name	Retention Time min	Area mAU*min	Height mAU	Relative Area %	Relative Height %	Amount n.a.
1		12,643	6,823	47,167	24,36	24,66	n.a.
2		13,012	12,465	85,871	44,50	44,90	n.a.
3		13,652	8,723	58,222	31,14	30,44	n.a.
**Total:**			**28,011**	**191,260**	**100,00**	**100,00**	

Figure S47: HPLC chromatogram after the reaction of substrate rac-**4** with PLZ2.0 at 205 nm; Method 2 (chiral, Chiralcel OJ-RH)

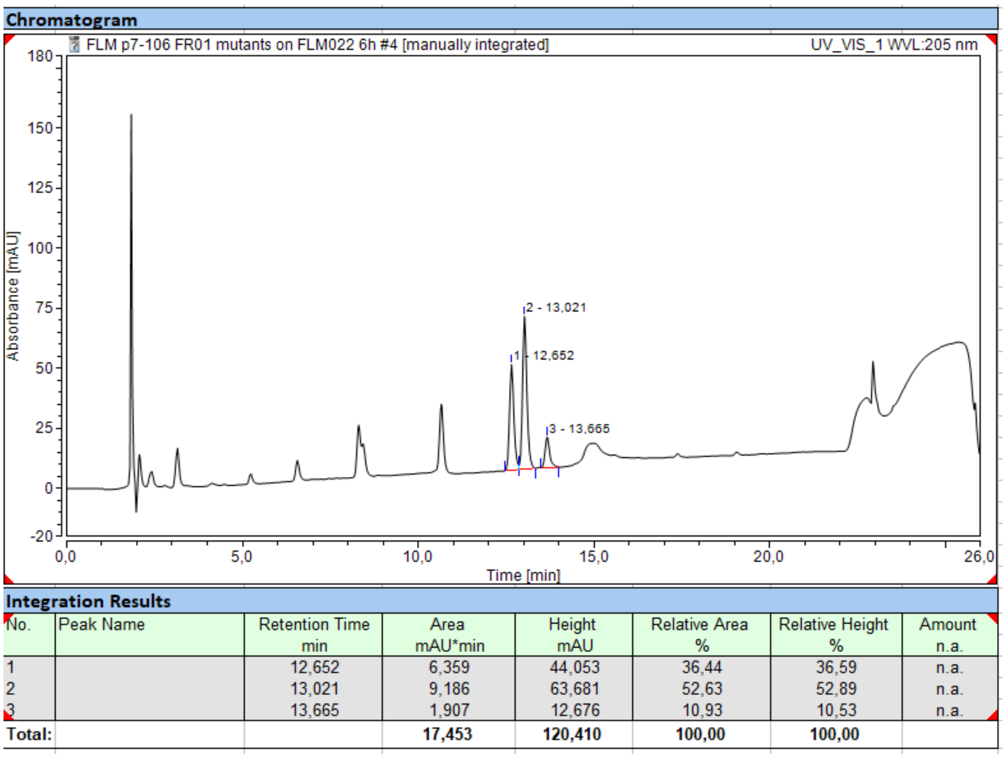

Integration Results

No.	Peak Name	Retention Time min	Area mAU*min	Height mAU	Relative Area %	Relative Height %	Amount n.a.
1		12,652	6,359	44,053	36,44	36,59	n.a.
2		13,021	9,186	63,681	52,63	52,89	n.a.
3		13,665	1,907	12,676	10,93	10,53	n.a.
**Total:**			**17,453**	**120,410**	**100,00**	**100,00**	

Figure S48: HPLC chromatogram after the reaction of substrate rac-**4** with PLZ2.1 at 205 nm; Method 2 (chiral, Chiralcel OJ-RH)

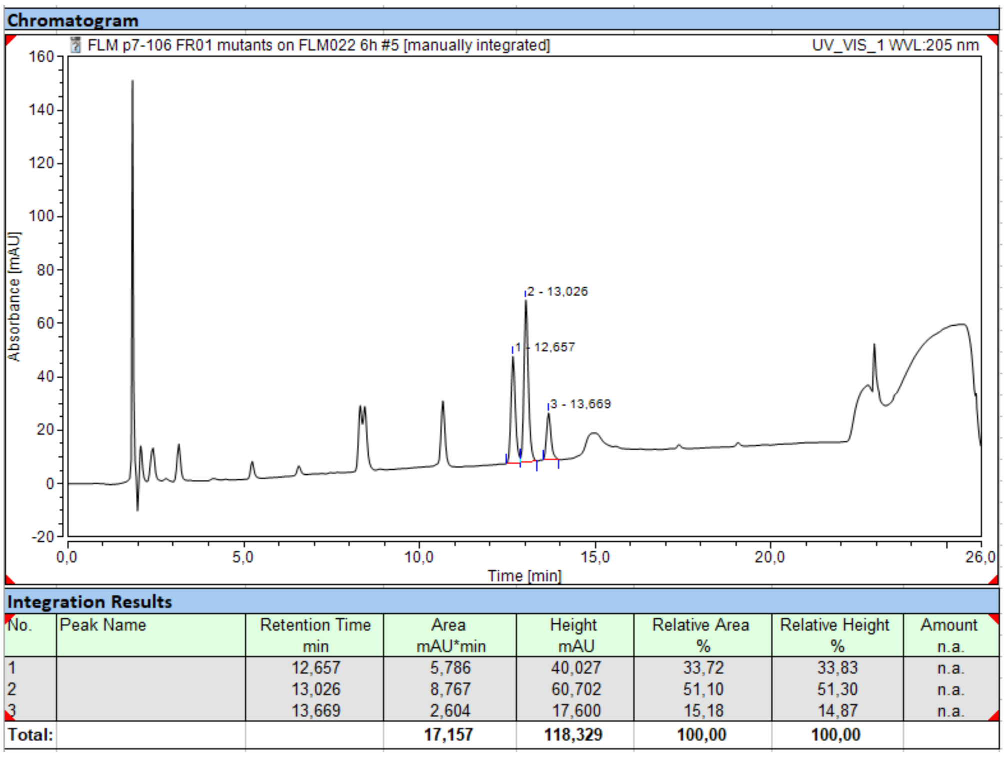

Integration Results

No.	Peak Name	Retention Time min	Area mAU*min	Height mAU	Relative Area %	Relative Height %	Amount n.a.
1		12,657	5,786	40,027	33,72	33,83	n.a.
2		13,026	8,767	60,702	51,10	51,30	n.a.
3		13,669	2,604	17,600	15,18	14,87	n.a.
**Total:**			**17,157**	**118,329**	**100,00**	**100,00**	

Figure S49: HPLC chromatogram after the reaction of substrate rac-**4** with PLZ2.2 at 205 nm; Method 2 (chiral, Chiralcel OJ-RH)

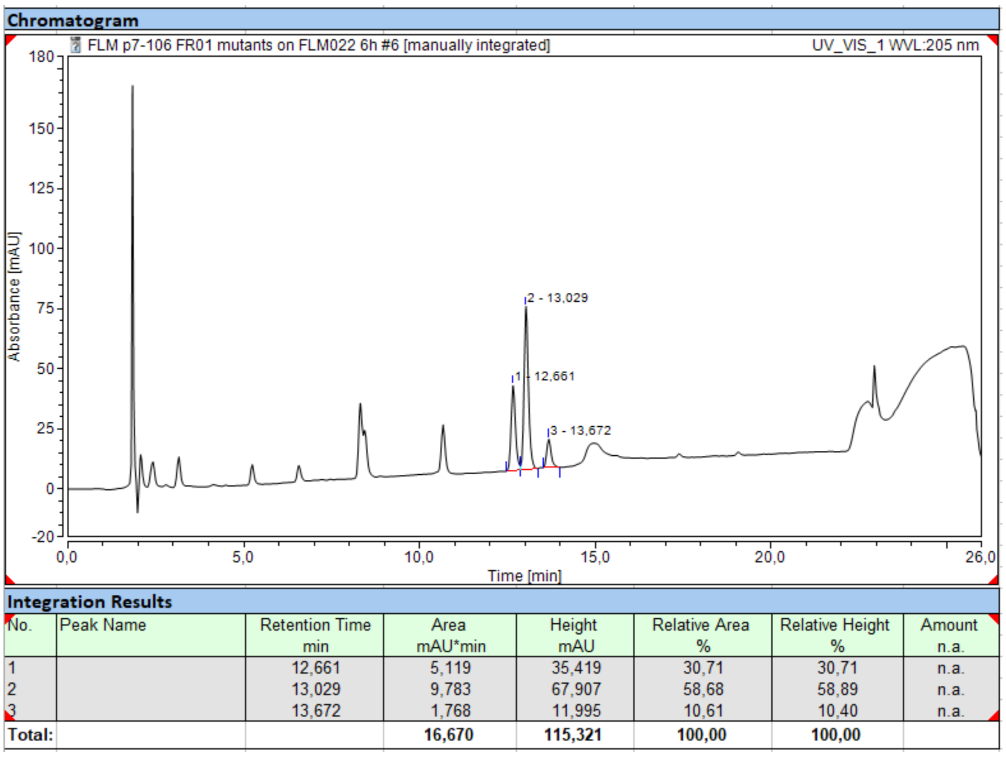

Integration Results

No.	Peak Name	Retention Time min	Area mAU*min	Height mAU	Relative Area %	Relative Height %	Amount n.a.
1		12,661	5,119	35,419	30,71	30,71	n.a.
2		13,029	9,783	67,907	58,68	58,89	n.a.
3		13,672	1,768	11,995	10,61	10,40	n.a.
**Total:**			**16,670**	**115,321**	**100,00**	**100,00**	

Figure S50: HPLC chromatogram after the reaction of substrate rac-**4** with PLZ2.3 at 205 nm; Method 2 (chiral, Chiralcel OJ-RH)

12. References

1. D. G. Gibson, L. Young, R.-Y. Chuang, J. C. Venter, C. A. Hutchison and H. O. Smith, Enzymatic assembly of DNA molecules up to several hundred kilobases, *Nat. Methods*, 2009, **6**, 343-345.
2. J. Chiu, D. Tillett, I. W. Dawes and P. E. March, Site-directed, Ligase-Independent Mutagenesis (SLIM) for highly efficient mutagenesis of plasmids greater than 8kb, *J. Microbiol. Methods*, 2008, **73**, 195-198.
3. A. S. Klein, F. Leiss-Maier, R. Mühlhofer, B. Boesen, G. Mustafa, H. Kugler and C. Zeymer, A De Novo Metalloenzyme for Cerium Photoredox Catalysis, *J. Am. Chem. Soc.*, 2024, **146**, 25976-25985.
4. C. L. Moore, L. J. Papa, III and M. D. Shoulders, A Processive Protein Chimera Introduces Mutations across Defined DNA Regions In Vivo, *J. Am. Chem. Soc.*, 2018, **140**, 11560-11564.
5. Schrodinger, LLC, The PyMOL Molecular Graphics System, Version 1.8, 2015.
6. J. L. Watson, D. Juergens, N. R. Bennett, B. L. Trippe, J. Yim, H. E. Eisenach, W. Ahern, A. J. Borst, R. J. Ragotte, L. F. Milles, B. I. M. Wicky, N. Hanikel, S. J. Pellock, A. Courbet, W. Sheffler, J. Wang, P. Venkatesh, I. Sappington, S. V. Torres, A. Lauko, V. De Bortoli, E. Mathieu, S. Ovchinnikov, R. Barzilay, T. S. Jaakkola, F. Dimaio, M. Baek and D. Baker, De novo design of protein structure and function with RFdiffusion, *Nature*, 2023, **620**, 1089-1100.
7. J. Dauparas, G. R. Lee, R. Pecoraro, L. An, I. Anishchenko, C. Glasscock and D. Baker, Atomic context-conditioned protein sequence design using LigandMPNN, *Nat. Methods*, 2025, **22**, 717-723.
8. Z. Lin, H. Akin, R. Rao, B. Hie, Z. Zhu, W. Lu, N. Smetanin, R. Verkuil, O. Kabeli, Y. Shmueli, A. Dos Santos Costa, M. Fazel-Zarandi, T. Sercu, S. Candido and A. Rives, Evolutionary-scale prediction of atomic-level protein structure with a language model, *Science*, 2023, **379**, 1123-1130.
9. M. Mirdita, K. Schütze, Y. Moriwaki, L. Heo, S. Ovchinnikov and M. Steinegger, ColabFold: making protein folding accessible to all, *Nat. Methods*, 2022, **19**, 679-682.
10. D.A. Case, H.M. Aktulga, K. Belfon, I.Y. Ben-Shalom, J.T. Berryman, S.R. Brozell, F.S. Carvahol, D.S. Cerutti, T.E. Cheatham, III, G.A. Cisneros, V.W.D. Cruzeiro, T.A. Darden, N. Forouzesh, M. Ghazimirsaeed, G. Giambaşu, T. Giese, M.K. Gilson, H. Gohlke, A.W. Goetz, J. Harris, Z. Huang, S. Izadi, S.A. Izmailov, K. Kasavajhala, M.C. Kaymak, I. Kolossv\'a ry, A. Kovalenko, T. Kurtzman, T.S. Lee, P. Li, Z. Li, C. Lin, J. Liu, T. Luchko, R. Luo, M. Machado, M. Manathunga, K.M. Merz, Y. Miao, O. Mikhailovskii, G. Monard, H. Nguyen, K.A. O'Hearn, A. Onufriev, F. Pan, S. Pantano, A. Rahnamoun, D.R. Roe, A. Roitberg, C. Sagui, S. Schott-Verdugo, A. Shajan, J. Shen, C.L. Simmerling, N.R. Skrynnikov, J. Smith, J. Swails, R.C. Walker, J. Wang, J. Wang, X. Wu, Y. Wu, Y. Xiong, Y. Xue, D.M. York, C. Zhao, Q. Zhu, and P.A. Kollman (2025), Amber 2025, University of California, San Francisco
11. D. A. Case, H. M. Aktulga, K. Belfon, D. S. Cerutti, G. A. Cisneros, V. W. D. Cruzeiro, N. Forouzesh, T. J. Giese, A. W. Götz, H. Gohlke, S. Izadi, K. Kasavajhala, M. C. Kaymak, E. King, T. Kurtzman, T.-S. Lee, P. Li, J. Liu, T. Luchko, R. Luo, M. Manathunga, M. R. Machado, H. M. Nguyen, K. A. O'Hearn, A. V. Onufriev, F. Pan, S. Pantano, R. Qi, A. Rahnamoun, A. Risheh, S. Schott-Verdugo, A. Shajan, J. Swails, J. Wang, H. Wei, X. Wu, Y. Wu, S. Zhang, S. Zhao, Q. Zhu, T. E. Cheatham, III, D. R. Roe, A. Roitberg, C. Simmerling, D. M. York, M. C. Nagan and K. M. Merz, Jr., AmberTools, *J. Chem. Inf. Model.*, 2023, **63**, 6183-6191.
12. D. A. Case, D. S. Cerutti, V. W. D. Cruzeiro, T. A. Darden, R. E. Duke, M. Ghazimirsaeed, G. M. Giambaşu, T. J. Giese, A. W. Götz, J. A. Harris, K. Kasavajhala, T.-S. Lee, Z. Li, C. Lin, J. Liu, Y. Miao, R. Salomon-Ferrrer, J. Shen, R. Snyder, J. Swails, R. C. Walker, J. Wang, X. Wu, J. Zeng, T. E. Cheatham Iii, D. R. Roe, A. Roitberg, C. Simmerling, D. M. York, M. C. Nagan and K. M. Merz, Jr., Recent Developments in Amber Biomolecular Simulations, *J. Chem. Inf. Model.*, 2025, **65**, 7835-7843.

13. J. Eberhardt, D. Santos-Martins, A. F. Tillack and S. Forli, AutoDock Vina 1.2.0: New Docking Methods, Expanded Force Field, and Python Bindings, *J. Chem. Inf. Model.*, 2021, **61**, 3891-3898.
14. O. Trott and A. J. Olson, AutoDock Vina: Improving the speed and accuracy of docking with a new scoring function, efficient optimization, and multithreading, *J. Comput. Chem.*, 2010, **31**, 455-461.
15. J.-E. Sánchez-Aparicio, L. Tiessler-Sala, L. Velasco-Carneros, L. Roldán-Martín, G. Sciortino and J.-D. Maréchal, BioMetAll: Identifying Metal-Binding Sites in Proteins from Backbone Preorganization, *J. Chem. Inf. Model.*, 2021, **61**, 311-323.
16. B. Webb and A. Sali, Comparative Protein Structure Modeling Using MODELLER, *Curr. Protoc. Bioinf.*, 2016, **54**, 5.6.1-5.6.37.
17. T. A. Halgren, R. B. Murphy, R. A. Friesner, H. S. Beard, L. L. Frye, W. T. Pollard and J. L. Banks, Glide: A New Approach for Rapid, Accurate Docking and Scoring. 2. Enrichment Factors in Database Screening, *J. Med. Chem.*, 2004, **47**, 1750-1759.
18. S. Passaro, G. Corso, J. Wohlwend, M. Reveiz, S. Thaler, V. R. Somnath, N. Getz, T. Portnoi, J. Roy, H. Stark, D. Kwabi-Addo, D. Beaini, T. Jaakkola and R. Barzilay, Boltz-2: Towards Accurate and Efficient Binding Affinity Prediction, *bioRxiv*, 2025, DOI: 10.1101/2025.06.14.659707, 2025.2006.2014.659707.
19. R. A. Friesner, J. L. Banks, R. B. Murphy, T. A. Halgren, J. J. Klicic, D. T. Mainz, M. P. Repasky, E. H. Knoll, M. Shelley, J. K. Perry, D. E. Shaw, P. Francis and P. S. Shenkin, Glide: A New Approach for Rapid, Accurate Docking and Scoring. 1. Method and Assessment of Docking Accuracy, *J. Med. Chem.*, 2004, **47**, 1739-1749.
20. R. Salomon-Ferrer, D. A. Case and R. C. Walker, An overview of the Amber biomolecular simulation package, *WIREs Comput. Mol. Sci.*, 2013, **3**, 198-210.
21. C. Tian, K. Kasavajhala, K. A. A. Belfon, L. Raguette, H. Huang, A. N. Migues, J. Bickel, Y. Wang, J. Pincay, Q. Wu and C. Simmerling, ff19SB: Amino-Acid-Specific Protein Backbone Parameters Trained against Quantum Mechanics Energy Surfaces in Solution, *J. Chem. Theory Comput.*, 2020, **16**, 528-552.
22. Z. Li, L. F. Song, P. Li and K. M. Merz, Jr., Parametrization of Trivalent and Tetravalent Metal Ions for the OPC3, OPC, TIP3P-FB, and TIP4P-FB Water Models, *J. Chem. Theory Comput.*, 2021, **17**, 2342-2354.
23. J.-P. Ryckaert, G. Ciccotti and H. J. C. Berendsen, Numerical integration of the cartesian equations of motion of a system with constraints: molecular dynamics of n-alkanes, *J.Comput. Phys.*, 1977, **23**, 327-341.
24. P. Schmidtke, A. Bidon-Chanal, F. J. Luque and X. Barril, MDpocket: open-source cavity detection and characterization on molecular dynamics trajectories, *Bioinformatics*, 2011, **27**, 3276-3285.
25. W. Humphrey, A. Dalke and K. Schulten, VMD: Visual molecular dynamics, *J. Mol. Graphics*, 1996, **14**, 33-38.
26. Flare™, version 3.0, Cresset®, Litlington, Cambridgeshire, UK; https://cresset-group.com/flare/
27. H. C. Kolb, M. S. VanNieuwenhze and K. B. Sharpless, Catalytic Asymmetric Dihydroxylation, *Chem. Rev.*, 1994, **94**, 2483-2547.
28. W. Kabsch, XDS, *Acta Crystallogr D Biol Crystallogr*, 2010, **66**, 125-132.
29. W. Kabsch, Automatic processing of rotation diffraction data from crystals of initially unknown symmetry and cell constants, *J. Appl. Crystallogr.*, 1993, **26**, 795-800.
30. A. J. McCoy, R. W. Grosse-Kunstleve, P. D. Adams, M. D. Winn, L. C. Storoni and R. J. Read, Phaser crystallographic software, *J. Appl. Crystallogr.*, 2007, **40**, 658-674.
31. J. Abramson, J. Adler, J. Dunger, R. Evans, T. Green, A. Pritzel, O. Ronneberger, L. Willmore, A. J. Ballard, J. Bambrick, S. W. Bodenstein, D. A. Evans, C.-C. Hung, M. O'Neill, D. Reiman, K. Tunyasuvunakool, Z. Wu, A. Žemgulytė, E. Arvaniti, C. Beattie, O. Bertolli, A. Bridgland, A. Cherepanov, M. Congreve, A. I. Cowen-Rivers, A. Cowie, M. Figurnov, F. B. Fuchs, H. Gladman, R. Jain, Y. A. Khan, C. M. R. Low, K. Perlin, A. Potapenko, P. Savy, S. Singh, A. Stecula, A.

Thillaisundaram, C. Tong, S. Yakneen, E. D. Zhong, M. Zielinski, A. Žídek, V. Bapst, P. Kohli, M. Jaderberg, D. Hassabis and J. M. Jumper, Accurate structure prediction of biomolecular interactions with AlphaFold 3, *Nature*, 2024, **630**, 493-500.
32. G. N. Murshudov, P. Skubák, A. A. Lebedev, N. S. Pannu, R. A. Steiner, R. A. Nicholls, M. D. Winn, F. Long and A. A. Vagin, REFMAC5 for the refinement of macromolecular crystal structures, *Acta Crystallogr D Biol Crystallogr*, 2011, **67**, 355-367.
33. P. V. Afonine, R. W. Grosse-Kunstleve, N. Echols, J. J. Headd, N. W. Moriarty, M. Mustyakimov, T. C. Terwilliger, A. Urzhumtsev, P. H. Zwart and P. D. Adams, Towards automated crystallographic structure refinement with phenix.refine, *Acta Crystallogr D Biol Crystallogr*, 2012, **68**, 352-367.
34. J. J. Headd, N. Echols, P. V. Afonine, R. W. Grosse-Kunstleve, V. B. Chen, N. W. Moriarty, D. C. Richardson, J. S. Richardson and P. D. Adams, Use of knowledge-based restraints in phenix.refine to improve macromolecular refinement at low resolution, *Acta Crystallogr D Biol Crystallogr*, 2012, **68**, 381-390.
35. P. Emsley, B. Lohkamp, W. G. Scott and K. Cowtan, Features and development of Coot, *Acta Crystallogr D Biol Crystallogr*, 2010, **66**, 486-501.
36. R. J. Morris, A. Perrakis and V. S. Lamzin, ARP/wARP and automatic interpretation of protein electron density maps, *Methods Enzymol.*, 2003, **374**, 229-244.
37. C. J. Williams, J. J. Headd, N. W. Moriarty, M. G. Prisant, L. L. Videau, L. N. Deis, V. Verma, D. A. Keedy, B. J. Hintze, V. B. Chen, S. Jain, S. M. Lewis, W. B. Arendall, 3rd, J. Snoeyink, P. D. Adams, S. C. Lovell, J. S. Richardson and D. C. Richardson, MolProbity: More and better reference data for improved all-atom structure validation, *Protein Sci.*, 2018, **27**, 293-315.
38. R. Nomura, T. Matsuno and T. Endo, Samarium Iodide-Catalyzed Pinacol Coupling of Carbonyl Compounds, *J. Am. Chem. Soc.*, 1996, **118**, 11666-11667.
